# Diphenyl Diselenide and SARS-CoV-2: *in silico* Exploration of the Mechanisms of Inhibition of Main Protease (M^pro^) and Papain-like Protease (PL^pro^)

**DOI:** 10.1021/acs.jcim.3c00168

**Published:** 2023-03-23

**Authors:** Folorunsho Bright Omage, Andrea Madabeni, Amanda Resende Tucci, Pablo Andrei Nogara, Marco Bortoli, Alice dos Santos Rosa, Vivian Neuza dos Santos Ferreira, João Batista Teixeira Rocha, Milene Dias Miranda, Laura Orian

**Affiliations:** † Departamento de Bioquímica e Biologia Molecular, Universidade Federal de Santa Maria, Santa Maria, Rio Grande do Sul 97105-900, Brazil; ‡ Dipartimento di Scienze Chimiche, Università Degli Studi di Padova, Via Marzolo 1, Padova 35131, Italy; § Laboratório de Vírus Respiratórios e Do Sarampo, Instituto Oswaldo Cruz, Fundação Oswaldo Cruz, Manguinhos, Rio de Janeiro 21041-210, Brazil; ∥ Laboratório de Morfologia e Morfogênese Viral, Instituto Oswaldo Cruz, Fundação Oswaldo Cruz, Manguinhos, Rio de Janeiro 21041-210, Brazil; ⊥ Institute of Computational Chemistry and Catalysis (IQCC) and Department of Chemistry, Faculty of Sciences, University of Girona, C/M. A. Capmany 69, Girona 17003, Spain

## Abstract

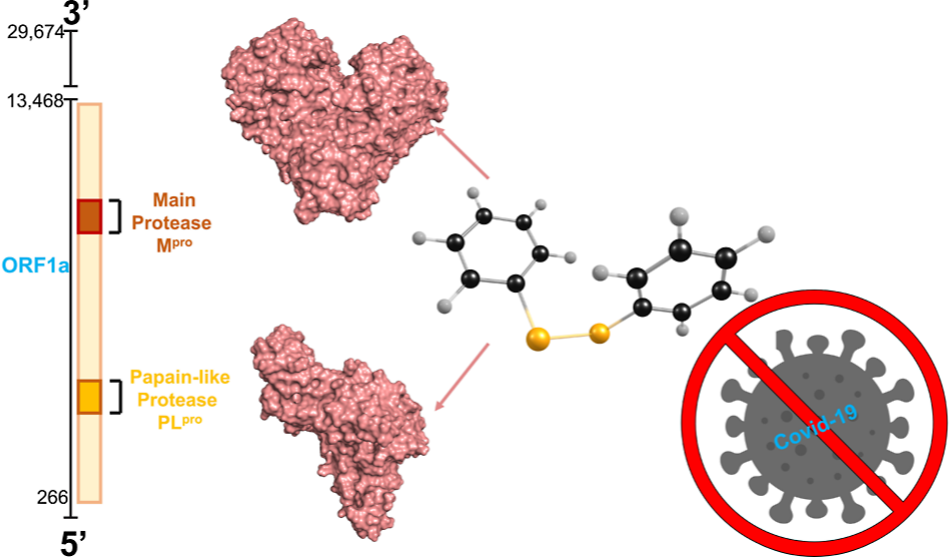

The SARS-CoV-2 pandemic has prompted global efforts to develop therapeutics. The main protease of SARS-CoV-2 (M^pro^) and the papain-like protease (PL^pro^) are essential for viral replication and are key targets for therapeutic development. In this work, we investigate the mechanisms of SARS-CoV-2 inhibition by diphenyl diselenide (PhSe)_2_ which is an archetypal model of diselenides and a renowned potential therapeutic agent. The **in vitro** inhibitory concentration of (PhSe)_2_ against SARS-CoV-2 in Vero E6 cells falls in the low micromolar range. Molecular dynamics (MD) simulations and density functional theory (DFT) calculations [level of theory: SMD-B3LYP-D3(BJ)/6-311G(d,p), cc-pVTZ] are used to inspect non-covalent inhibition modes of both proteases via π-stacking and the mechanism of covalent (PhSe)_2_ + M^pro^ product formation involving the catalytic residue C145, respectively. The **in vitro** CC_50_ (24.61 μM) and EC_50_ (2.39 μM) data indicate that (PhSe)_2_ is a good inhibitor of the SARS-CoV-2 virus replication in a cell culture model. The *in silico* findings indicate potential mechanisms of proteases’ inhibition by (PhSe)_2_; in particular, the results of the covalent inhibition here discussed for M^pro^, whose thermodynamics is approximatively isoergonic, prompt further investigation in the design of antiviral organodiselenides.

## 1 Introduction

The SARS-CoV-2 main and papain-like proteases, M^pro^ and PL^pro^, are essential targets in the fight against this virus because they play a key role for its replication. These proteases have no equivalent enzymatic analogues in humans and thus no similar cleavage specificity, implying that their inhibition will likely have low toxicity.^1−3^

Recent events have shown a sudden increase in the number of variants with Omicron bearing the largest number of mutations.^4^ Interestingly, no mutations have been observed in the conserved catalytic dyad/triad of M^pro^/PL^pro^, suggesting that an effective antiviral drug against SARS-CoV-2 targeting these proteases is a promising pharmacological strategy.^5^

Based on the recent literature, the inhibitory effects of SARS-CoV-2 M^pro^ and PL^pro^ by the popular organoselenide ebselen are explained by the formation of a selenylsulfide (Se–S) involving C145.^2,5−11^ Besides the direct interaction with the catalytic C145, Menéndez et al. suggested that the inhibition of M^pro^ by ebselen may also be related to the interaction between protein domains II and III, a region which is essential for the dimerization of the protease.^12^ Other aspects that remain almost unexplored are the potential interaction of organoselenium compounds with other residues, including cysteinyl residues not located in the active site, and with metabolites of ebselen.^13^

**Scheme 1:**
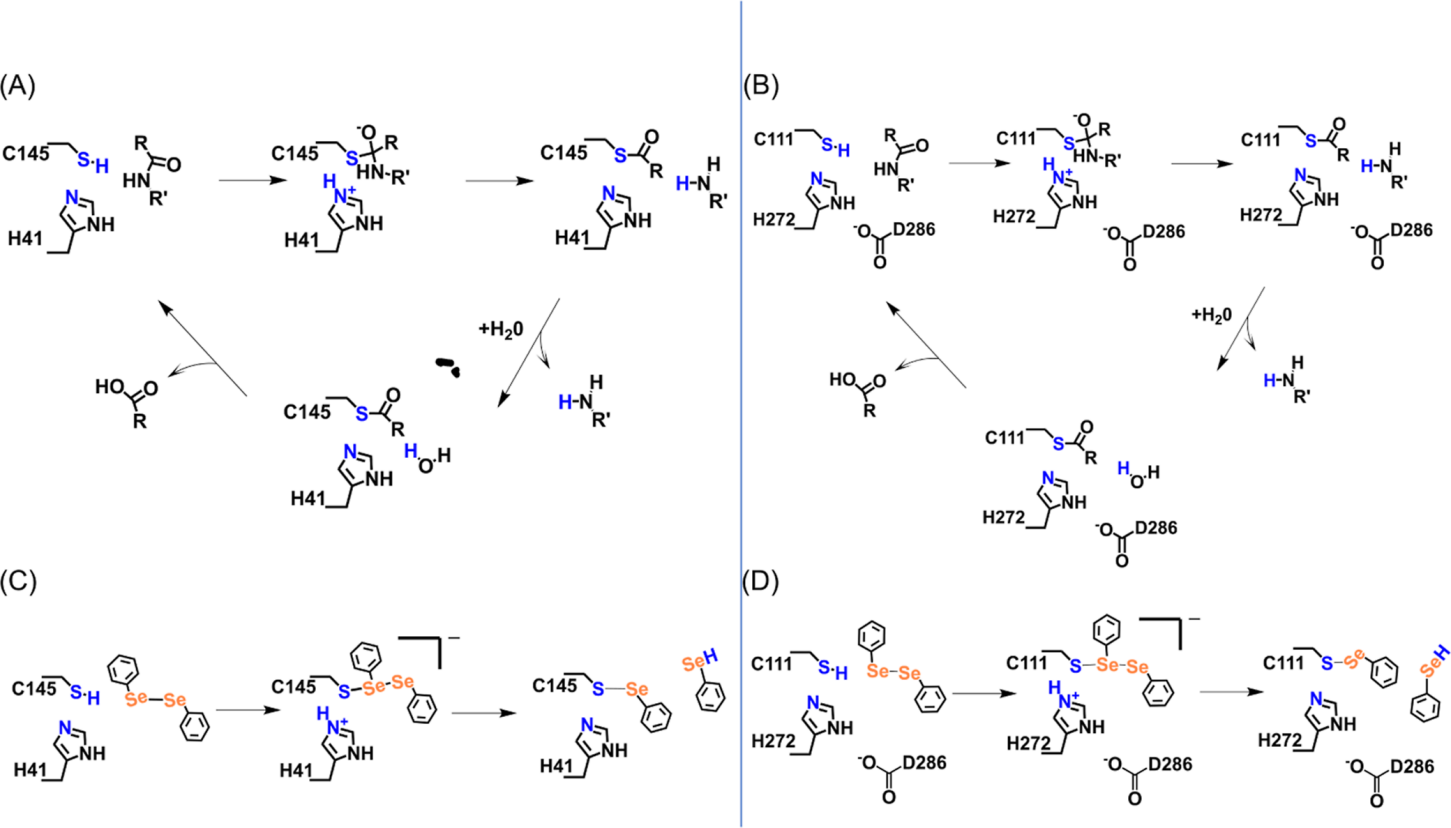
(A) M^pro^ and (B) PL^pro^ Acylation and Deacylation Steps Involving Hydrolysis and Recovery of Reactant Complexes. (C) Proposed Mechanism of Covalent Inhibition of SARS-CoV-2 M^pro^ and (D) PL^pro^ by (PhSe)_2_

The catalytic sites of M^pro^ and PL^pro^ show strong similarity: the former protease has a catalytic dyad (H41 and C145), while the latter has a catalytic triad (C111, H272, and D286).^11^ The protonation state of cysteine and histidine residues before nucleophilic attack on substrates was investigated for M^pro^ using different computational approaches (cluster-DFT, QM/MM, and a thorough MD study), which revealed that the couple of residues is more stable in the neutral state than in the zwitterionic form.^6,14−16^ The activation of the catalytic dyad/triad (through a proton transfer from the C to the H residue) promotes the nucleophilic attack on the carbonyl carbon atom of a peptide bond of the substrate by the sulfur atom of C145(S-)/C111(S-) (Scheme 1A,B), thus leading to a tetrahedral thiohemiketal (THA) intermediate.^17^ The cleavage of the peptide bond occurs via a back proton transfer from the protonated H41/H272 to the nitrogen atom of the substrate. The peptide is released from the active site as a water molecule that attacks the carbonyl carbon atom of the peptide, together with the proton transfer to H41/H272 (Scheme 1A,B). The covalent bond between C145 and the peptide of this protonated intermediate is then broken to release the second product of the reaction in the deacylation phase, and the zwitterion is finally neutralized.

In the presence of an organoselenium ligand, the formation of a covalent S–Se bond with C145/C111 interrupts the normal C-protease activity (Scheme 1C,D),^17^ as the inhibitor–enzyme complex formed by covalently bonded molecules such as ebselen and analogues hampers the subsequent steps in the cycle.^6,14,18^

The PL^pro^ active site has a canonical Cys protease catalytic triad (C111, H272, and D286), while M^pro^ has a catalytic dyad (C145 and H41 residues).^19,20^ In PL^pro^, C111 acts as a nucleophile by cleaving the substrate peptide bond, and H272 and D286 residues act as an acid–base pair promoting the thiol (Cys) deprotonation and thus enhancing its nucleophilicity.^21,22^ Therefore, D286 is essential for PL^pro^ catalytic activity. On the other hand, M^pro^ has a catalytic dyad, and the proton transfer occurs between C145 and H41. H41 presents a hydrogen bond with a water molecule, which also has a hydrogen bond with the side chain of D187. However, D187 interacts with the R40 residue by a strong salt bridge.^20^ Thus, in M^pro^, the role of D187 is more structural than catalytic.

Diselenides are an important class of organoselenium compounds that have been studied mainly for their antioxidant and anti-inflammatory potential.^23−26^ (PhSe)_2_ is the parent compound of diaryl diselenides and displays weak electrophilic potential.^27,28^ Conversely, in terms of electrophilicity, ebselen, which is a selenenylamide, possesses strong electrophilic potential.^29^ The toxicological and pharmacological interaction of (PhSe)_2_ with different targets has been studied *in silico*(13,24,30−33) and *in vitro*,^34^ and antiviral^9,35^ and antifungal activity^36^ was reported. Thus, (PhSe)_2_ is interesting because it represents an archetypal model or scaffold of diselenide compounds and because it is a renowned potential therapeutic agent with inhibitory potency against M^pro^ in the low micromolar range and, consequently, has pharmacological significance.^23^ Of pharmacological significance, the plasmatic concentration of (PhSe)_2_ after oral administration of relatively high doses to rodents is in the micromolar range.^7,23,37^ In this work, after verifying the *in vitro* (PhSe)_2_ effect against SARS-CoV-2 replication, we sought a possible mechanism of action through an *in silico* approach. For this purpose, we thoroughly analyze *in silico* the structural and chemical mechanism of SARS-CoV-2 proteases’ inhibition by (PhSe)_2_, employing molecular docking, molecular dynamics (MD), and density functional theory (DFT) protocols. After docking the ligand, the non-covalent complex of (PhSe)_2_ with M^pro^ and PL^pro^ is generated by MD simulation of the equilibrated system. DFT calculations are used to investigate the plausible mechanistic reaction steps taking place in the active site cluster; this approach has been carried out only for (PhSe)_2_ + M^pro^.

## 2 Materials and Methods

### 2.1 *in vitro* Assays

All the tested compounds were resuspended in 100% dimethyl sulfoxide (DMSO) for the *in vitro* assays, aliquoted, and stored at −20 °C. A maximum of three freezing and thawing cycles were performed to maintain chemical stability and avoid compound degradation.^38^ In fact, (PhSe)_2_ is very stable in DMSO solution when kept at 8 °C for more than 1 month (unpublished results). In the assays, the DMSO final concentrations were equal to or lower than 0.1% (v/v) when diluted in Dulbecco’s modified Eagle’s medium (DMEM-Gibco), not affecting the growth of the cells.^39,40^

Vero E6 cells (*Cercopithecus aethiops*; kidney epithelial) were infected with the SARS-CoV-2 isolate (GISAID EPI ISL #414045) in multiplicity of infection (MOI) 0.01 using DMEM with 0.01% N-2-hydroxyethylpiperazine-N’-2-ethanesulfonic acid (Gibco). After 1 h, the supernatants were harvested, and the cells were incubated with (PhSe)_2_ at different concentrations (from 0.78 to 12 μM). After 24 h of infection, the supernatants were removed and the virus titrated by plaque forming unit assay (PFU/mL).^41,42^

For PFU assay, monolayers of Vero E6 cells (2 × 10^4^ cells/well) in 96-well plates were exposed to 50 μL of supernatant dilutions for 1 h at 37 °C in 5% CO_2_. After this, 50 μL of semi-solid high-glucose DMEM containing 2% fetal bovine serum and 2.4% carboxymethylcellulose (CMC) was added, and cultures were incubated for 3 days at 37 °C. Then, the cells were fixed with 100 μL of 10% formalin for 3 h at room temperature. The cell monolayer was stained with 0.04% solution of crystal violet in 20% ethanol for 1 h, and PFUs were counted. The virus titers were determined by PFU/mL.^42^

All procedures related to virus culture were handled at a biosafety level 3 (BSL3) multiuser facility, according to WHO guidelines.^43^

For cytotoxicity analysis, monolayers of Vero E6 (10^4^ cells/well) in 96-well plates were treated for 72 h with different concentrations of all compounds tested. Then, 5 mg/mL 3-(4,5-dimethylthiazol-2-yl)-2,5-diphenyltetrazolium bromide (MTT—Sigma) in 1X phosphate buffered saline (PBS) was added to the cells, according to manufacturer’s instructions. After 2 h at 37 °C, 10% sodium dodecyl sulfate was added. After incubating for 2 h at 37 °C, the plates were read in a spectrophotometer at 570 nm.

All experiments were carried out at least three independent times, including a minimum of two technical replicates in each assay. The dose–response curves used to calculate the EC_50_ and CC_50_ values were generated by a variable slope plot from Prism GraphPad software 8.0. The equations used to fit the best curve were generated based on *R*^2^ values ≥0.9.

### 2.2 *in silico* Investigation

#### 2.2.1 System Preparation and Molecular Docking

AutoDock Vina software^44,45^ was used to simulate the binding pose of (PhSe)_2_ with M^pro^ (PDB ID 6LU7(2)) and PL^pro^ (PDB ID 7JN2). The water of crystallization, ions, and ligands were removed during preparation, while the hydrogen atoms were added using the CHIMERA program; 100 steps of energy minimization (amberff99SB) followed.^46^ Both the catalytic dyad and triad were considered neutral, as previously reported.^47,48^ The M^pro^’s grid box was centered at xyz coordinates of −14.04, 17.44, and 66.22, with box sizes of 25, 35, and 25 Å. PL^pro^ was located in a 20 × 20 × 20 Å cubic box centered at xyz coordinates of 39.64, 30.68, and 1.66. For each ligand–receptor complex, 20 (PhSe)_2_ binding poses were generated. The lowest binding free energies for M^pro^ and PL^pro^ were found to be −6.2 and −4.9 kcal mol^–1^, respectively. The best Se–S(C) orientation was chosen as the binding pose model and used as the starting point in the MD simulations.

#### 2.2.2 Molecular Dynamics (MD) Simulations

The AMBER 20^49^ pmemd.cuda engine was used to perform all MD simulations in explicit water. The residue numbering used in our study is identical to that used in the 6LU7 PDB file for M^pro^; for PL^pro^, numbering begins with residue 4 as obtained from the 7JN2 PDB file, which was reassigned as 1 during file preparation for simulation using pdb4amber. The AMBER ff14SB force field^50^ was used for the proteases, while the general amber force field (GAFF) parameters for (PhSe)_2_ were taken from the work by Torsello et al.^51^

For completeness, the set of parameters for this ligand is reported in the Supporting Information (Tables 1S1). For the zinc finger of PL^pro^, the zinc AMBER force field (ZAFF) was used.^52^ For PL^pro^, the tetravalent binding site for Zn^2+^ is formed by C189, C192, C224, and C226 and was thus treated as Zn-CCCC using the ZAFF.

**Table 1:** (PhSe)_2_ Inhibition Activity in Vero E6 Cells

	Vero E6		
molecules	CC_50_^a^ (μM)	EC_50_^b^ (μM)	SI^c^(CC_50_/EC_50_)
(PhSe)_2_	24.61 ± 4.55	2.39 ± 1.51	10.30

a CC_50_, the concentration required to reduce normal, non-infected cell viability by 50%. Values represent the mean ± SEM of duplicate samples from three independent experiments.

b EC_50_, the concentration required to reduce inhibition of viral infection-induced cytopathogenicity by 50%. Values represent the mean ± SEM of duplicate samples from three independent experiments.

c SI, selectivity index determined by the ratio between CC50 and EC50.

Each protease with the diselenide ligand was solvated in a truncated octahedral water box, using the TIP3P water model^53^ requiring a minimum distance of 12 Å between the solute and the box border. The total number of water molecules was 18605 (Apo M^pro^), 23453 (Apo PL^pro^), 18555 [(PhSe)_2_ + M^pro^], and 29325 [(PhSe)_2_ + PL^pro^]. The simulated systems have a total number of atoms equal to 60504 (Apo M^pro^), 75257 (Apo PL^pro^), 60378 [(PhSe)_2_ + M^pro^], and 92899 [(PhSe)_2_ + PL^pro^]. A NaCl concentration of 0.15 M was used, with four Na^+^ ions added to neutralize the net charge of the M^pro^ complex; no addition of extra ions was necessary to neutralize the PL^pro^ complex.

The systems’ initial gradient minimization stage was completed in two rounds. A strong harmonic constraint of 100 kcal mol^–1^ Å^2^ was applied to the solute atoms in the first round of minimization, and 6,000 steps of minimization (1000 steps of steepest descent minimization + 5000 steps of conjugate gradient minimization) were performed. The second minimization consisted of 4000 steps with no constraints (1000 steps of steepest descent minimization + 3000 steps of conjugate gradient minimization).

After the minimization, each system was gradually heated to 298 K over 50 ps. Finally, a 50 ns simulation under isothermal–isobaric (*NPT*) conditions was performed before proceeding to production simulation. After a 50 ns equilibration (Figure S5 in the Supporting Information), the system was subjected to a 200 ns simulation for (PhSe)_2_ + M^pro^, a 200 ns simulation for the Apo M^pro^ structure, a 100 ns simulation for PL^pro^ + (PhSe)_2_, and a 100 ns simulation for Apo PL^pro^ using a 2 fs integration time step. Throughout the simulation, the temperature was controlled by a Langevin thermostat,^54^ and the pressure was maintained at 1 bar by a Berendsen barostat.^55^ During the production runs, coordinates were saved every 20 ps, yielding a total of 10,000 structures for a 200 ns long MD trajectory. The trajectories were visualized using VMD 1.9.363^56^ and Discovery Studio.^57^ The trajectories of the (PhSe)_2_ + M^pro^/PL^pro^ systems were analyzed through CPPTRAJ^58^ to generate the root mean square deviation (RMSD) plots. The number of (PhSe)_2_ – M^pro^ residue contacts was determined with a threshold of 4.5 Å. Using the covariance matrix obtained from our 3D data, for a principal component analysis, the 2D projections with respect to selected eigenvector components were plotted.

The last 50 ns interval of the MD trajectories of each system was used to compute the binding free energy between (PhSe)_2_ and M^pro^/PL^pro^. To this end, we initially used the molecular mechanics with generalized Born and surface area solvation (MM-GBSA) method,^59^ which is commonly used for proteins.^60,61^ The snapshots were sampled at 200 ps intervals, yielding a total of 251 frames for calculating the MM-GBSA energies. The binding free energy Δ*G*_bind_ was calculated using the MM-GBSA as follows
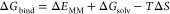
1

In this case, Δ*E*_MM_ is the sum of non-bonded and bonded interaction energies.^62^ Δ*G*_solv_ is the sum of the polar and non-polar solvation contributions, where the polar terms are calculated using a generalized Born model solver and the non-polar terms are computed based on the size of the solvent-accessible surface area in (PhSe)_2_ and M^pro^/PL^pro^. The last term, which corresponds to conformational entropy *T*Δ*S*, is computationally expensive, and previous studies^63,64^ demonstrated that including the entropic contribution in the Δ*G*_bind_ calculations does not guarantee a better agreement of the calculated binding free energy with experimental data due to the inherent approximate nature of the calculation of the entropic term. All MM-GBSA calculations in this study were performed with the AmberTools-20^58^ MMPBSA.py script.^65^ The per residue decomposition analyses (idecomp = 1) using the MD trajectories were also performed to identify the key (PhSe)_2_–residue energetic contributors to the binding free energy.^66^

In order to better quantify the binding energy in the case of the M^pro^ system, we calculated the binding energy also with molecular mechanics Poisson–Boltzmann surface area (MM-PBSA) which is an analogous method to estimate ligand binding affinities and is implemented in AmberTools-20 software.

#### 2.2.3 DFT Calculations

Two enzymatic clusters were extracted from the MD simulations. In both cases, the M^pro^ residues within a cutoff of 4.0 Å from (PhSe)_2_, i.e., H41, M49, C145, and M165, were chosen and removed from the catalytic pocket; the CH_3_CO and CH_3_NH groups were added to the N- and C-terminal regions, respectively, to mimic the backbone peptide bonds. The total number of atoms was 134. Gaussian16^67^ was used to perform all DFT calculations. The B3LYP hybrid functional^68,69^ was employed and combined with the Grimme D3 dispersion correction and the Becke–Johnson damping function.^70,71^ The 6-311G(d,p) basis set was used to describe all first and second period atoms, while Dunning’s correlation-consistent cc-pVTZ basis set was used to describe sulfur and selenium atoms. All structures were optimized in the gas phase and in a solvent (water) using the SMD solvation model^72^ [level of theory: (SMD-)B3LYP-D3(BJ)/6-311G(d,p), cc-pVTZ] keeping frozen the backbone atoms (see Tables S11–S13 in the Supporting Information for a complete list of the frozen atoms). Solvent effects were included using a continuum model as done also in previous studies on enzymatic clusters.^73−75^ Thermodynamic corrections were calculated using standard statistical mechanics relations based on electronic energies and gas phase frequency calculations at 298.15 K and 1 atm, as implemented in Gaussian software. All energies described in the main text are relative Gibbs free (Δ*G*) energies. To assess the nature of the optimized geometries, frequency calculations were performed: each transition state has one imaginary frequency that is related to the normal mode connecting the preceding and the following intermediate. All minima have no imaginary frequencies. For validation purposes, starting from the transition states, an intrinsic reaction coordinate (IRC) calculation was performed, to ensure that the proper transition state was located.

## 3 Results and Discussion

### 3.1 Experimental Results

**Figure 1:**
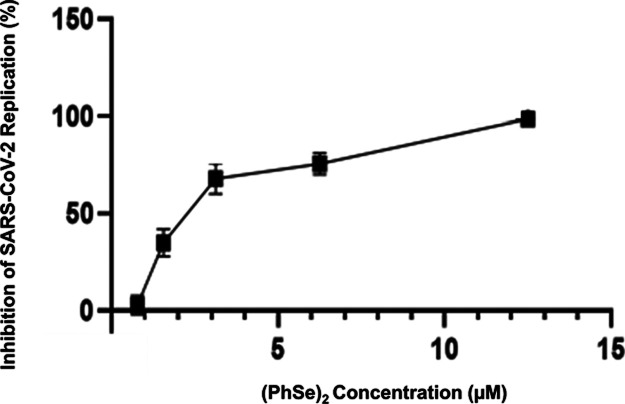
(PhSe)_2_ inhibits SARS-CoV-2 replication in Vero E6 infected cells. Data points are expressed as mean ± SEM, and when the error is not visible, the bars are hidden by the symbols.

To assess the efficacy of (PhSe)_2_ in SARS-CoV-2 replication inhibition, we performed *in vitro* assays using the Vero E6 model of cells. Cells infected with SARS-CoV-2 were treated with different concentrations of (PhSe)_2_ (Figure 1). The EC_50_ and CC_50_ values are shown in Table 1. We analyzed the cell toxicity by MTT assay. The CC_50_ for Vero E6 cells was about 25 μM, and the EC_50_ for SARS-CoV-2 in this cell model was 2.4 μM. The EC_50_ value is comparable to that of observed FDA-approved drugs repurposed for the treatment of COVID-19 during 2020.^76^ Moreover, the (PhSe)_2_ EC_50_ is lower than that of other M^pro^ inhibitors, including ebselen.^77,78^ In addition, it is important to highlight that the beneficial effect of organoselenium compounds is not restricted only for inhibition of virus replication but also has potential beneficial effects in COVID-19 with a number of targets critical to pathogenesis, such as attenuation of inflammatory oxidants and cytokines, as already observed for ebselen, being interesting to also be evaluated for (PhSe)_2_.^23,27−29,31,78^

### 3.2 Non-Covalent (PhSe)_2_ + M^pro^/PL^pro^ Systems

The MD simulations shed light on the dynamic evolution in water of both M^pro^ and PL^pro^ in the presence of the ligand. Upon analyzing the MD trajectories of the (PhSe)_2_ + M^pro^ and (PhSe)_2_ + PL^pro^ systems (details are in Materials and Methods Section 2.2.2), analogies among the (PhSe)_2_ interactions with the proteases were observed: (i) stacking of the (PhSe)_2_ phenyl rings with H41 in M^pro^ and H272 in PL^pro^ and (ii) hydrophobic interactions of the rings with M49 and M165 in M^pro^ and W106, Y112, Y264, Y268, and L162 in PL^pro^.

**Figure 2:**
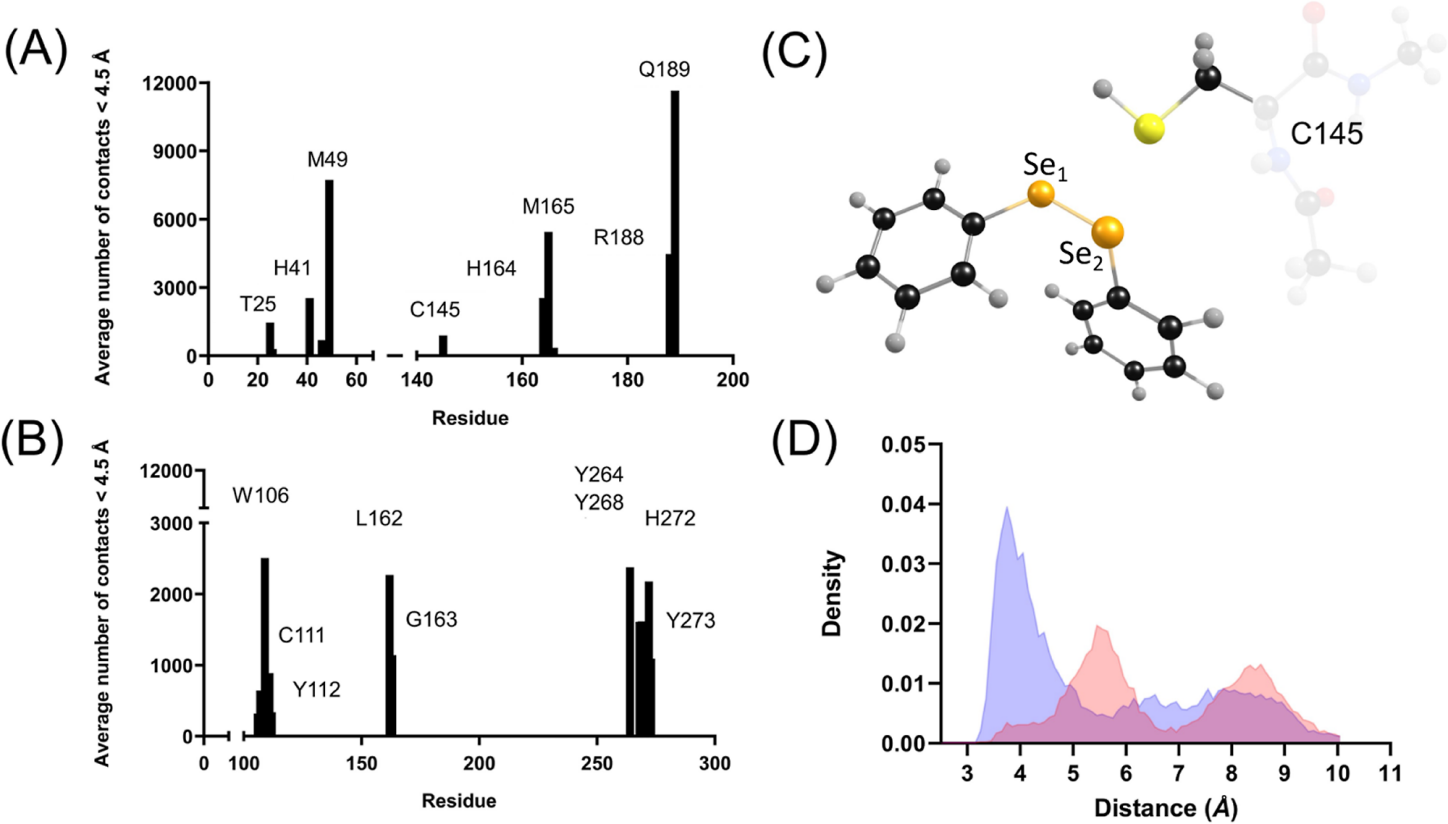
Average number of contacts between (PhSe)_2_ and (A) SARS-CoV-2 M^pro^ and (B) PL^pro^. Cut-off distance: 4.5 Å. (C) Structural details of (PhSe)_2_ in the active site of M^pro^, showing the mutual arrangement of Se atoms (Se_1_ and Se_2_) and S of C145. (D) Probability densities in M^pro^ of the distances between C145–S and Se_1_ of (PhSe)_2_, in blue, and Se_2_ of (PhSe)_2_, in red. The probability densities were obtained using the radial distribution function, which is the probability of finding a pair of atoms a distance *r* apart relative to the probability for a completely uniform distribution.

Using a distance threshold of 4.5 Å, a total of 38,132 contacts with (PhSe)_2_ were recorded throughout the simulation of (PhSe)_2_ + M^pro^ (Figure 2A). T25 accounts for approximately 4% of total contacts, M165 accounts for approximately 14% of total contacts, M49 accounts for approximately 20% of total contacts, and Q189 accounts for approximately 31% of total contacts. The two dyad residues, H41 and C145, account for about 9% of the total contacts. In (PhSe)_2_ + M^pro^, contacts with the conserved residues are continuously maintained, thus establishing a favorable binding region for (PhSe)_2_ in the M^pro^ binding site.

In PL^pro^ (Figure 2B), (PhSe)_2_ maintains contacts with the triad’s C111, H272, and D286 residues. The distance of D286 from (PhSe)_2_ exceeds 4.5 Å, and it acts as a stabilizer for the H272 proton exchange function in catalysis. Apart from the triad’s residues, (PhSe)_2_ makes several contacts with W106, Y112, L162, G163, Y264, Y268, and Y273 at less than 4.5 Å.

**Figure 3:**
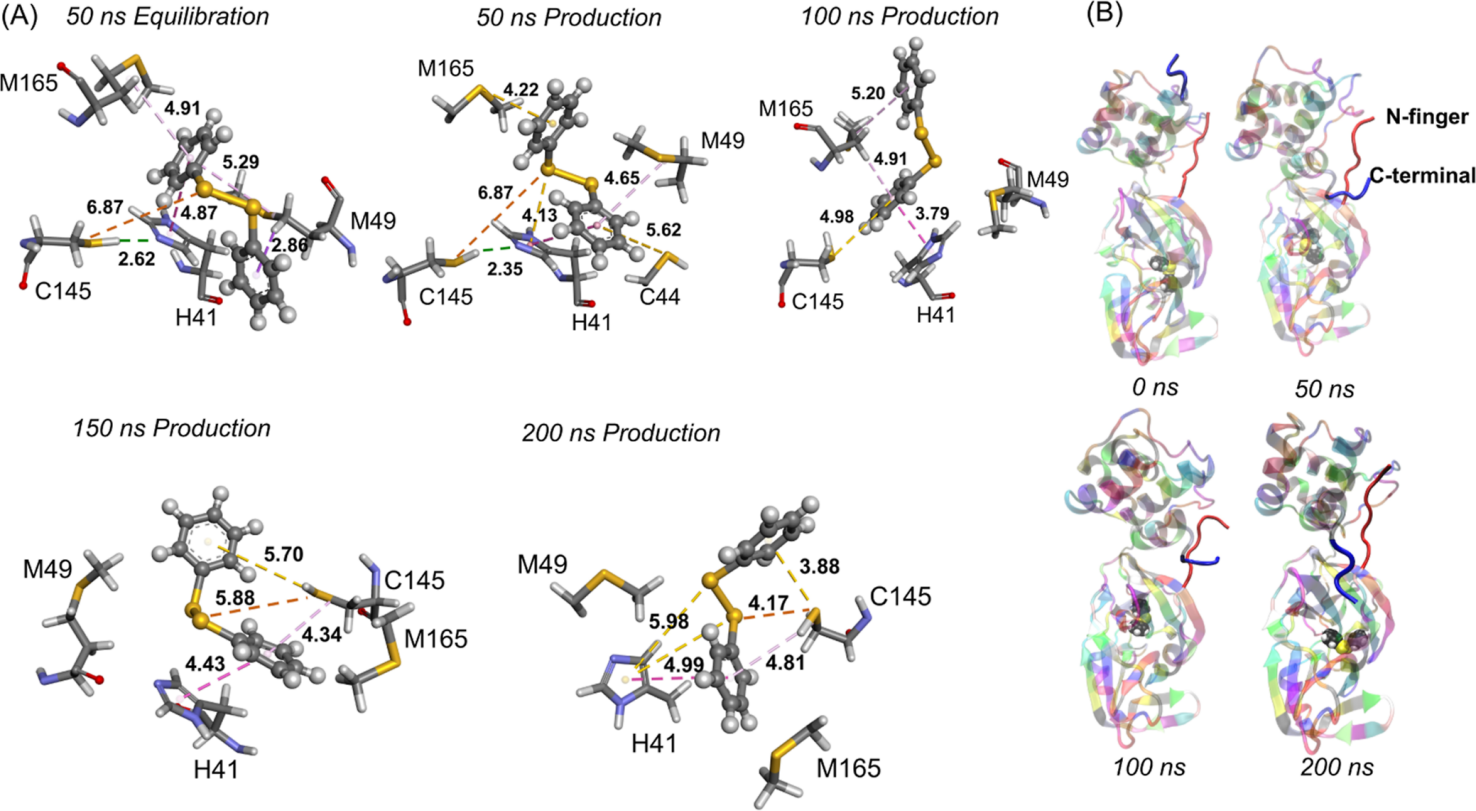
(A) (PhSe)_2_ + M^pro^ snapshots taken at different times during the simulation. The Se–S distance is represented in brown; the π–π stacking interaction is represented in pink; π–sulfur interaction with M49, M165, C44, and C145 is shown in gold and the C145-Sγ to Nε atom of H41 in green. (B) N-terminal (red) and C-terminal (blue) of the (PhSe)_2_ + M^pro^ system.

In terms of proximity to the S atom of C145, (PhSe)_2_ poses in the active site of SARS-CoV-2 M^pro^ remain very similar to the starting docking pose and differ only in their stacking conformation during the MD simulations. The S atom of C145 remains close to both Se atoms of (PhSe)_2_ (Figure 2C,D). According to the distributions shown in Figure 2D, the most probable values of the distances S–Se_1_ are 3.7 Å and S–Se_2_ are 5.4 Å. The larger distances observed in the second peak are configurations that correspond to a conformational change of the side chain of C145 from trans to gauche to accommodate the stabilizing H41 π-stacking interaction (Figure 3A). Snapshots taken at different times during the simulation are shown in Figure 3. The analysis of RMSD of the protein with the ligand shows that this system configuration is stable over time (200 ns) (see the next section). In (PhSe)_2_ + M^pro^, the catalytic dyad remains hydrogen-bonded with the thiol proton pointing toward the Nε atom of H41 up to 50 ns of simulation time before C145 assumes gauche conformation.

On the C-terminal of both the (PhSe)_2_ + M^pro^ system and the Apo (or ligand-free) structure, which has been simulated too, a significant conformational change is observed during the simulation. This can be observed in Figure 3B, where the original direction of the C-terminal at 0 ns is maintained until about 30 ns; then, it flips to a conformation which is conserved for the rest of the simulation. This latter appears to be the stable conformation of the protease (Figure 3B). Upon analyzing the interaction of the diselenide with the closest residues, it is seen that M49, M165, and Q189 interact via hydrophobic, π–alkyl, and π–sulfur interactions. Y54 favors a π–π stacking interaction (4.13 Å at 75 ns) in combination with the π–sulfur interaction involving C44. (PhSe)_2_ conformation changes continuously, but one of its phenyl rings remains in a π–π stacking position with H41.

**Figure 4:**
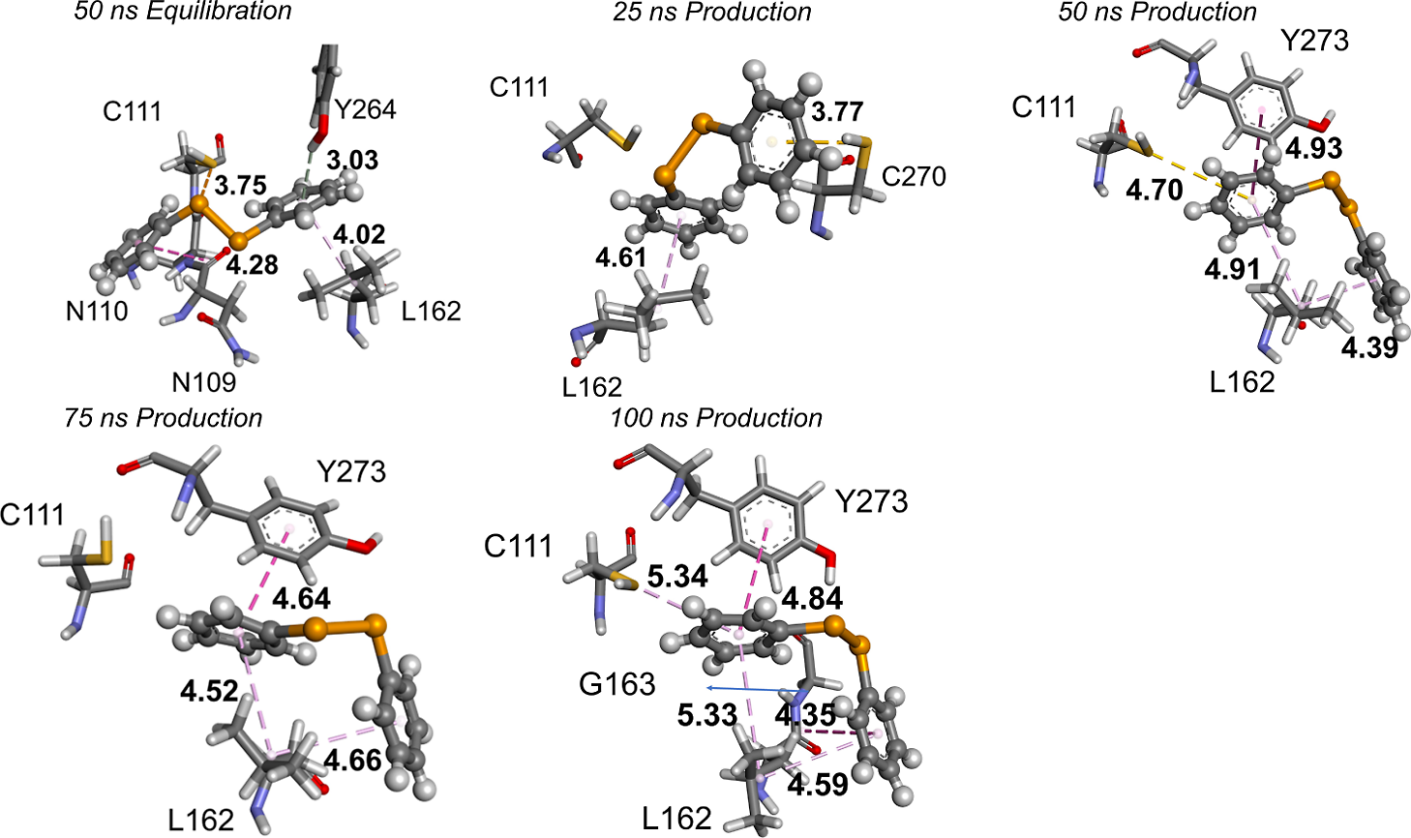
(PhSe)_2_ + PL^pro^ snapshots. The S–Se distance is represented in brown; the π–π stacking interaction is represented in pink; π–sulfur interaction with C111 is shown in gold.

Considering (PhSe)_2_ + PL^pro^ at 50 ns, i.e., at the end of the equilibration, the protease has several π–alkyl and π–hydrogen interactions with one of the phenyl rings of the diselenide (Figure 4). These interactions change over time to more stabilizing π–stacking interactions with Y273. In addition, the phenyl rings of (PhSe)_2_ are involved in π–interactions with the propyl group of the L162 side chain. Due to their mobility, the phenyl rings also establish interactions with N109, N110, C111, G163, and C270.

We found evidence that the ligand well fits in both proteases and remains close to the catalytic key residues, without any constraint. Its non-covalent inhibitory capacity was further explored by quantifying the stability of the systems and the interaction energies, as described in the next sections.

#### 3.2.1 Stability of (PhSe)_2_ + M^pro^/PL^pro^ Systems during MD Simulations

**Figure 5:**
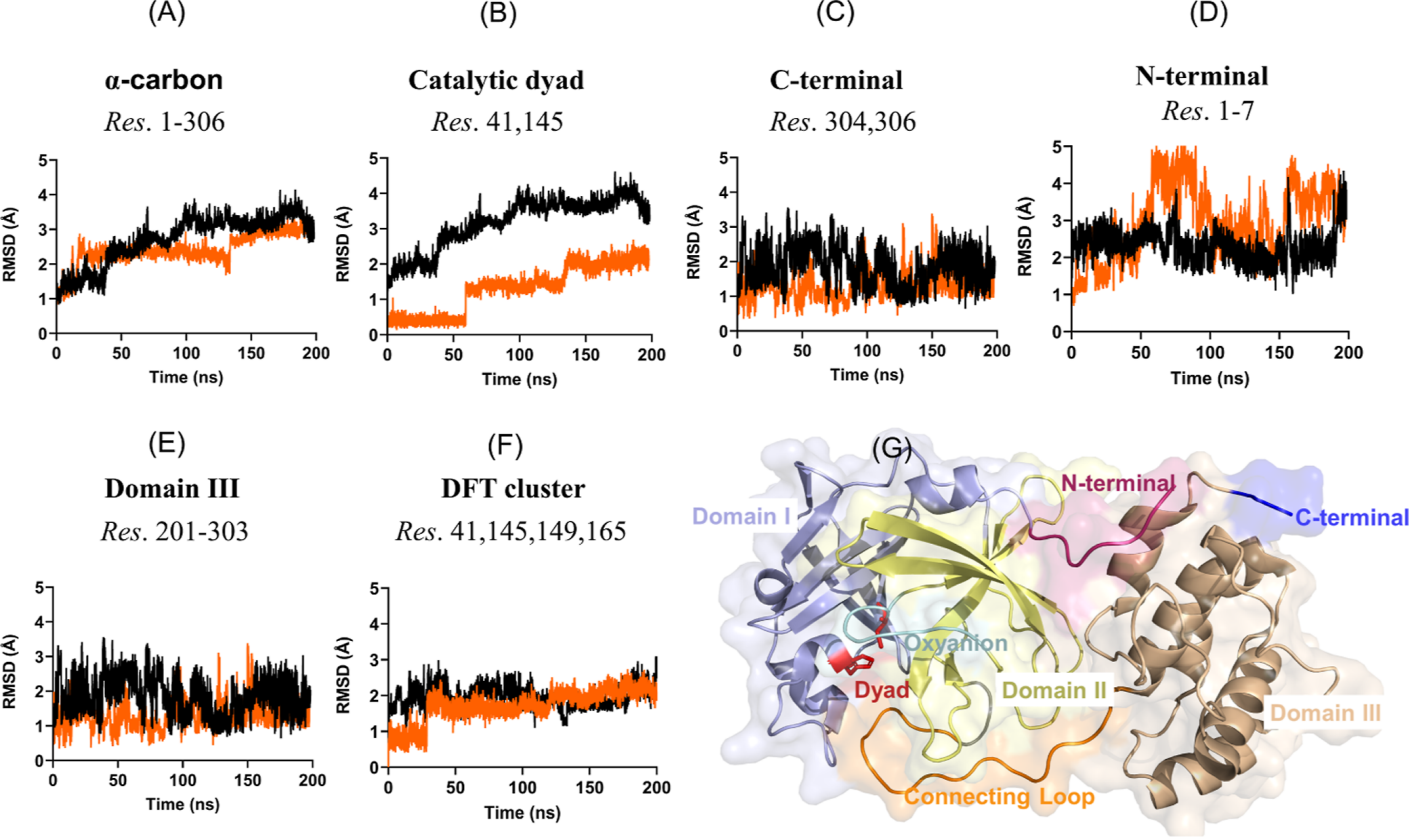
RMSD plots of the (A) α-carbon backbone, (B) catalytic dyad, (C) C-terminal, (D) N-terminal, (E) domain III, and (F) residues used to extract the DFT cluster (vide infra). The average RMSD of (PhSe)_2_ + M^pro^ is represented in orange and the Apo structure in black. (G) The catalytic dyad is colored red, the N-terminal magenta, the C-terminal blue, domain I light blue, domain II pale-yellow, domain III wheat, the oxyanion loop cyan, and the connecting loop orange.

The stability of the (PhSe)_2_ + M^pro^ non-covalent system was analyzed referring to fluctuations of different regions (see Figures 5 and S3 in the Supporting Information). As shown in Figure 5A, the α-carbon RMSD plots of the Apo and the (PhSe)_2_ + M^pro^ system show the effect of the ligand binding on the protein dynamics. The average backbone RMSD variation of (PhSe)_2_ + M^pro^ is in the range 1.0–2.9 Å, when compared to that in the range 1.0–3.5 Å for the Apo M^pro^ structure, clearly suggesting that the binding of the diselenide has only a limited impact on the structural dynamics of the enzyme. Then, we analyzed the stability of (PhSe)_2_ in the C145–H41 catalytic binding site, which exhibits an average RMSD in the range of 0.2–2.1 Å (Figure 5B). These low RMSD values, i.e., <3 Å, indicate that (PhSe)_2_ dynamics does not affect the compactness of the catalytic site, which was further ascertained by computing the average radius of gyration (*R*_g_) resulting in 22.3 Å for the Apo structure and 22.2 Å for (PhSe)_2_ + M^pro^ (Figure S2). These results indicate that the influence of (PhSe)_2_ binding on the overall M^pro^ structure is negligible. Small RMSD fluctuations are observed also in the oxyanion loop (Figure S3B in the Supporting Information) and in the long loop connecting domains II and III (Figure S3A in the Supporting Information) and C and N-terminals (Figure 5C,D). We found that domains I and II remain quite stable throughout the simulation with backbone RMSD changes being within ∼0.5–0.9 Å (Figure S3C,D in the Supporting Information). Conversely, domain III, which features five helices, exhibits higher fluctuations exceeding 3.0 Å (Figure 5E). These findings are fully consistent with previous stability reported behavior for ligand + M^pro^ systems.^48,79^ Finally, we also analyzed the changes displayed by the residues that compose the active site and used them in the cluster for the DFT calculations to model the covalent inhibition mechanism (vide infra). Both the Apo and the M^pro^-(PhSe)_2_ complex showed very similar stable rmsds at approximately 2 Å, a sign that the active site does not undergo strong modification during the simulation, and the presence of (PhSe)_2_ does not drastically change the active site conformation.

**Figure 6:**
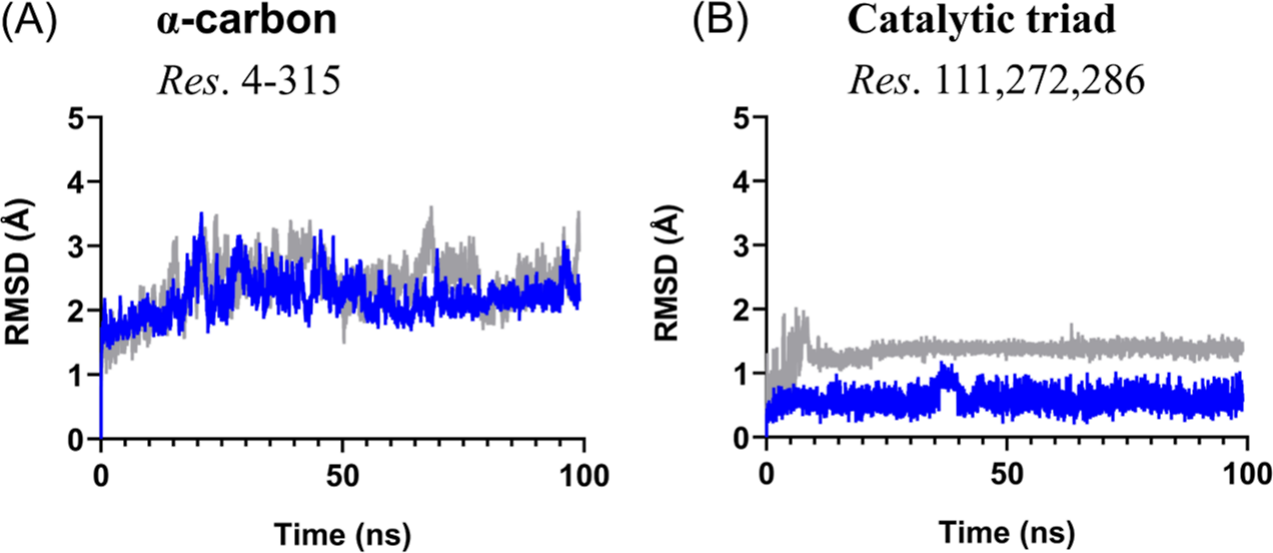
RMSD plots of the (A) backbone and (B) catalytic triad. The average RMSD of (PhSe)_2_ + PL^pro^ is represented in blue and the Apo structure in gray.

Also, the conformational changes of (PhSe)_2_ + PL^pro^ and Apo PL^pro^ were evaluated using the RMSD analysis (Figure 6). The two systems show an average RMSD of 2.20 Å for the (PhSe)_2_ + PL^pro^ and of 2.40 Å for the Apo PL^pro^ structure. The compactness of the two systems is also similar, as illustrated by *R*_g_ (Figure S2B). The modest impact of the binding of (PhSe)_2_ to PL^pro^ can be appreciated in the triad’s stability when compared to Apo PL^pro^ with 1 Å change in the RMSD.

**Figure 7:**
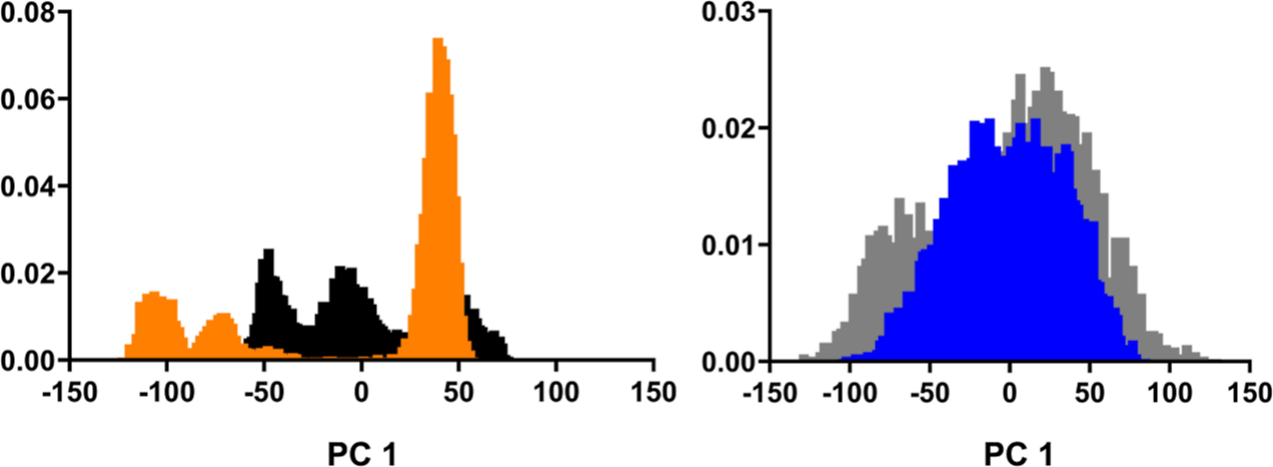
Overlap of principal component 1 (PC1) histograms from PCA in Cartesian space. (A) (PhSe)_2_ + M^pro^ (orange) and Apo M^pro^ (black). (B) (PhSe)_2_ + PL^pro^ (blue) and Apo PL^pro^ (gray).

When comparing principal component (PC) projections between different MD simulations, we obtain a criterion of similarity between the dominant modes of motion sampled along the different trajectories. PCA is performed in such a way that PC1 (the first principal component) exhibits the greatest variance in the sampled motion. We performed PCA on the trajectories of both (PhSe)_2_ + protease and Apo protease. Figure 7 shows the overlap of histograms of PC 1 projections for the simulations.

The simulations for (PhSe)_2_ + M^pro^ and Apo M^pro^ have different distributions for PC1, but the number of modes is conserved, indicating that these motions are not disruptive, which is consistent with the observations of the RMSD plots (Figure 5A). Conversely, the simulations of (PhSe)_2_ + PL^pro^ and Apo PL^pro^ have significant overlap, suggesting that similar dynamic modes were sampled.

Based on the equilibrium trajectories, MM-GBSA calculations were carried out to explore the binding affinity between (PhSe)_2_ and M^pro^ (Table S5)/PL^pro^ (Table S6). The binding free energy of (PhSe)_2_ to M^pro^ is −19 kcal mol^–1^; in the case of PL^pro^, it is −23 kcal mol^–1^. These values indicate a neatly favorable stabilizing role of both catalytic pockets toward this ligand. To identify the M^pro^/PL^pro^ residues involved in the (PhSe)_2_ binding, the computed free energies were decomposed into single residue contributions.

**Figure 8:**
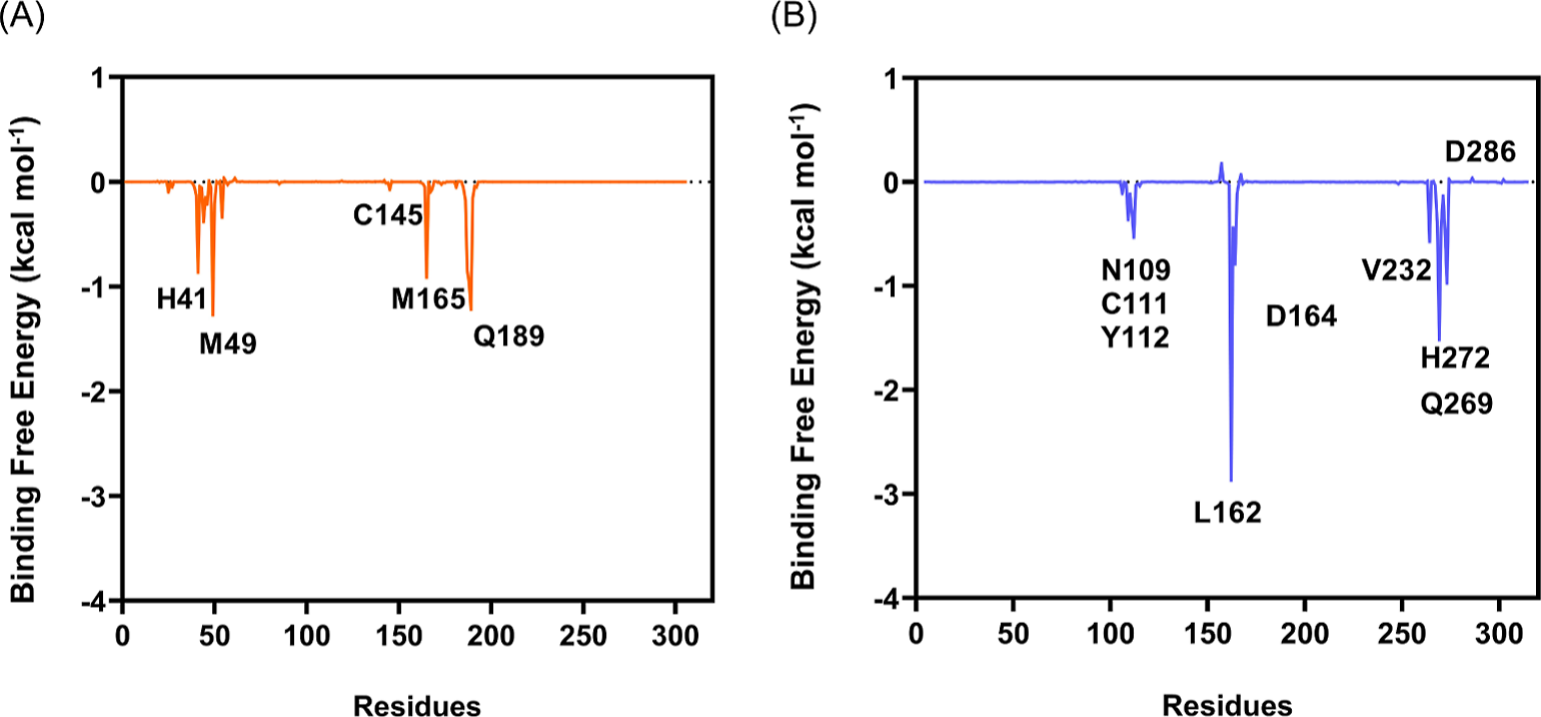
Binding free energy (kcal mol^–1^) decomposition per residue, for (A) (PhSe)_2_ + M^pro^ (orange) and (B) (PhSe)_2_ + PL^pro^ (blue).

The energy decomposition (Figure 8) indicates that the four conserved residues (H41, M49, M165, and Q189) are those that mostly contribute to the binding of (PhSe)_2_ and M^pro^. Regarding PL^pro^, L162, D164, Q269, and H272 are the four residues providing the highest contributions to the binding energy, mainly via hydrophobic and alkyl−π interactions (L162) and π-stacking (H272); the whole decomposition is provided in the Supporting Information (Tables S7 and S8).

Binding energies obtained with the MM-PBSA approach were calculated for M^pro^ with results showing a binding energy of −5 kcal mol^–1^ for the last 50 ns of the trajectory. To ensure the accuracy of this result, we also computed the binding affinity using the whole production trajectory of 200 ns (for a total of 1000 snapshots). Results showed a slightly more favorable binding activity of −8 kcal mol^–1^ (the full decomposition of the binding energies can be found in Supporting Information, Tables S9 and S10) which confirms the favorable ligand–protein binding in M^pro^.

#### 3.2.2 Torsional Motions of (PhSe)_2_ in M^pro^ and PL^pro^

**Figure 9:**
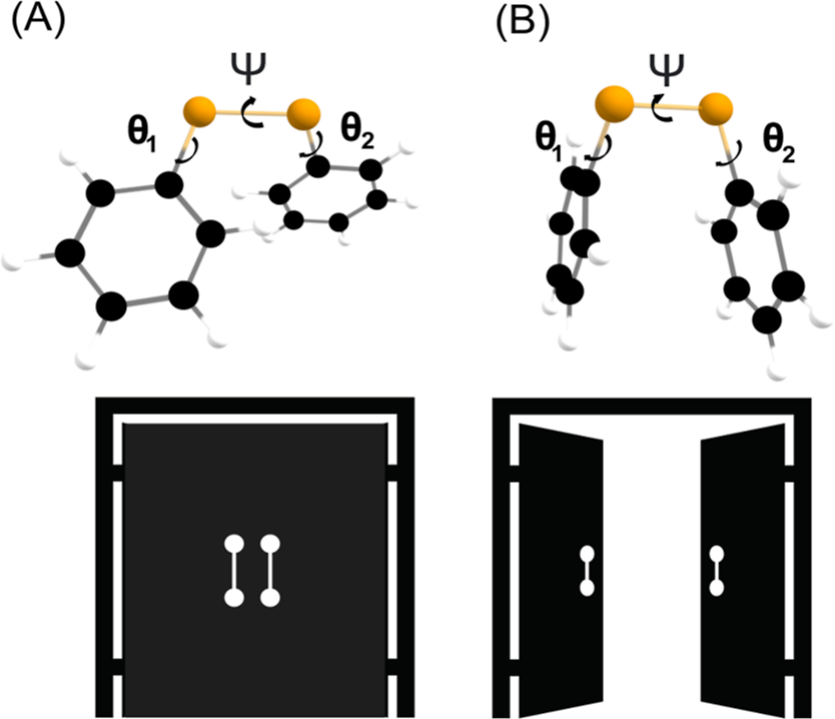
(A) Closed and (B) open conformations of (PhSe)_2_ with the main dihedral angles C–Se–Se–C (Ψ) and C–C–Se–Se (θ_1_ and θ_2_).

It is well known that the phenyl rings of (PhSe)_2_ freely rotate in solution.^51,80,81^ This motion can be followed by measuring the values of the dihedrals θ_1_ and θ_2_ (Figure 9). Conversely, the value of the main dihedral Ψ is close to 90 or −90°, characterizing two conformations which easily interconvert at room temperature. An accurate computational analysis showed that (PhSe)_2_ has two minimum energy structures corresponding to two distinct mutual orientations of the phenyl rings, denoted closed and open conformations^51,75,80^ (Figure 9).

**Figure 10:**
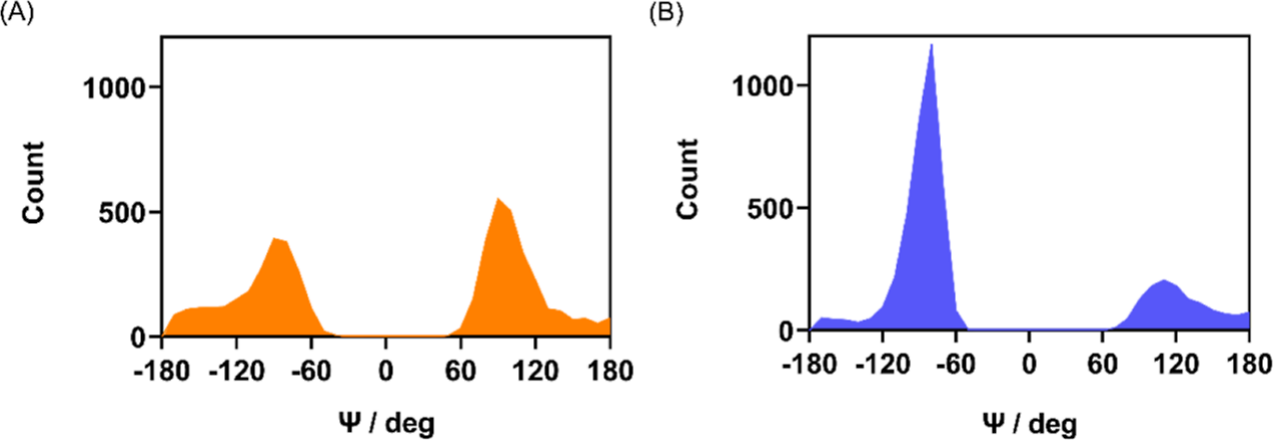
Distributions of the dihedral Ψ in the catalytic pocket of (A) M^pro^ and (B) PL^pro^.

This conformational behavior was observed also in the catalytic pockets of M^pro^ and PL^pro^ during the MD simulations. The dihedral angle Ψ and the dihedrals θ_1_ and θ_2_ were examined during the 200 ns simulation time. The Ψ distributions in M^pro^ and PL^pro^ closely resemble the results of free (PhSe)_2_ in water, with peaks close to ±90°,^51^ although in protein, they are not perfectly symmetric, and the peak intensities are not equivalent (Figure 10) due to specific interactions with the surrounding residues.

**Figure 11:**
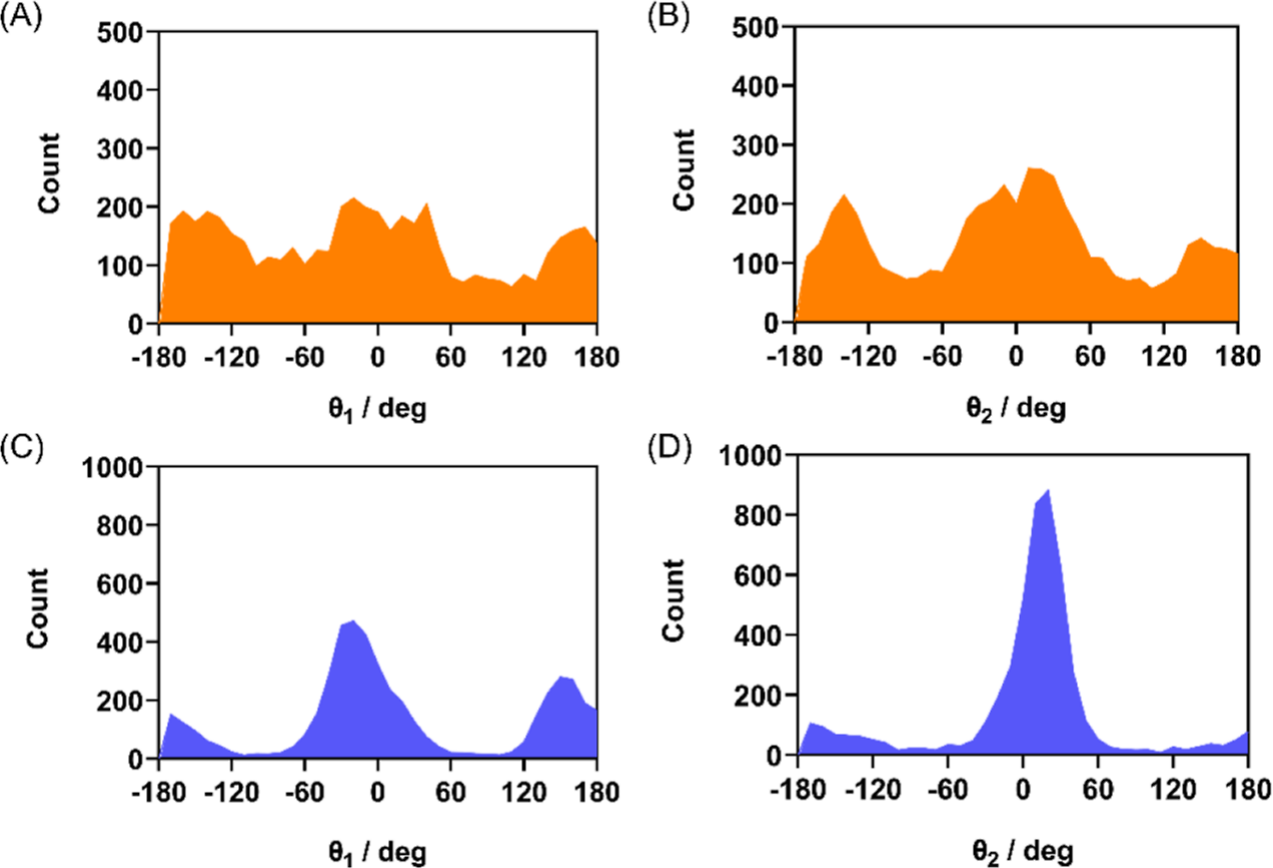
Distributions of the dihedrals θ_1_ and θ_2_ in (A) and (B) M^pro^ and (C) and (D) PL^pro^.

During the simulations, in M^pro^ and PL^pro^, the Se–Se bond length is well maintained, with average values of 2.37 and 2.28 Å, respectively. The same is true for the C–Se/C′–Se′ bonds and C–Se–Se′/C′–Se′–Se angles, which have average values of 1.97 Å and 103° for (PhSe)_2_ in M^pro^ and 1.95 Å and 107° for (PhSe)_2_ in PL^pro^, respectively. Regarding the orientation of the phenyl rings of (PhSe)_2_, in both proteases, the distributions are similar, with the closed conformer being more easily found (peaks at 0 and 180°, Figure 11). These findings are consistent with Torsello’s results^51^ and with experimental findings^82^ in solution.

The dynamics of (PhSe)_2_ characterized by flip-flop and rotation of the phenyl rings is nicely maintained in protein, suggesting that also in the complex anisotropic environment, the conformational barriers are modest.^83^ The dominant Ψ dihedral conformation at +90° and at θ_1_ and θ_2_ values of 0°/180° for diphenyl diselenide in M^pro^ was chosen for the DFT mechanistic investigation on the M^pro^ cluster.

### 3.3 M^pro^ Covalent Inhibition by (PhSe)_2_

The mechanism of covalent inhibition of these proteases by (PhSe)_2_ implies the formation of a S–Se bond with cleavage of the Se–Se bond of the ligand. Deprotonation of the catalytic cysteine is mandatory since it activates the nucleophilic potential of the chalcogen, and in both cases, proton transfer from Cys to His may be postulated. As described above, the RMSD of (PhSe)_2_ + M^pro^ shows a very stable dyad with minimal fluctuations and an average value of 1.0 Å, whereas in the absence of (PhSe)_2_, as in the case of the Apo, the average RMSD increases to 3.0 Å (Figure 5B). The stability of (PhSe)_2_ + M^pro^ limits the conformational freedom of C145 and H41 residues. Similar stable behavior is seen in the PL^pro^ catalytic triad (C111, H272, and D286) (Figure 6B) with a lower average RMSD of 0.57 Å, much lower than the RMSD of the triad of the Apo structure (1.34 Å). Analysis of the contacts reveals that (PhSe)_2_ remains in the catalytic pocket of M^pro^ and of PL^pro^.

**Figure 12:**
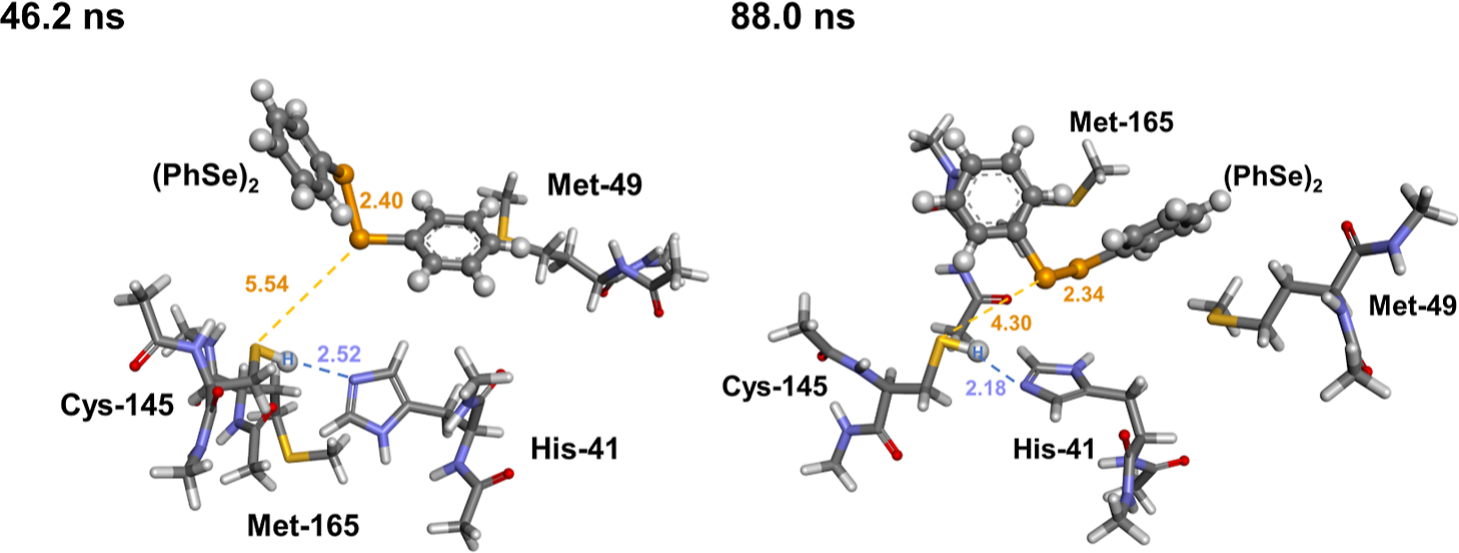
Optimized catalytic clusters (RC) for the mechanistic investigation of covalent M^pro^ inhibition by (PhSe)_2_, taken from MD snapshots at 46.2 ns (left) and at 88 ns (right). Level of theory: SMD(Water)-B3LYP-D3(BJ)/6-311G(d,p),ccPVTZ.

In Scheme 1C,D, our proposed mechanism of activated nucleophilic C sulfur attacking selenium in the diselenide ligand to form an inhibited product (PInhb) is shown. Due to this close analogy, we have focused on the mechanistic details of covalent inhibition using a cluster extracted from (PhSe)_2_ + M^pro^. In order to probe the effect of different conformations of the residues and of the arrangement of (PhSe)_2_ within the catalytic pocket on the reaction, two different enzymatic clusters were extracted from the MD simulations. A cluster was extracted at 46.2 ns with a Ψ value of −91° and a S–Se–Se angle amplitude of ≈80°. For the mechanistic study, all key residues C145, H41, M165, and M49, except Q189 [distance from (PhSe)_2_ > 4.0 Å], were included in a cluster which was treated at (SMD-)B3LYP-D3(BJ)/6-311G(d,p),ccPVTZ. The coordinates of the second reactant complex (RC) were extracted from an equilibrated 88 ns MD snapshot which has the dominant Ψ at +94° and C145-Sγ to the Nε atom of H41 at 2.16 and 4.30 Å to the selenium atom of (PhSe)_2_. Importantly, all the included residues are conserved residues and are found within 4 Å from (PhSe)_2_ and have been all verified by the contact analysis and MD energy decomposition to play important roles in the binding of (PhSe)_2_. The backbone atoms of the catalytic pocket were constrained to maintain the conformation found in the protein environment as obtained from the MD simulations at the corresponding times (the full list of constrained atoms in the cluster can be found in the Supporting Information). Capping groups were used to saturate terminal residues mimicking a peptide bond. At the N-terminus, an acetyl group was linked, and at the C-terminus, an amide group was connected (Figure 12). Poses at later times were not investigated due to a change of position of the (PhSe)_2_ molecule at about 100 ns (see movie in the Supporting Information) which makes the proposed mechanism unfeasible due to the phenyl rings of the ligand being found between Cys145 and His41, making the initial proton transfer difficult to model (vide infra).

For both clusters, similar mechanistic features were investigated, and the energetics of the two inhibition mechanisms were comparable. Thus, only results obtained starting from the snapshot at 46.2 ns will be discussed. Results obtained starting from the snapshot at 88 ns can be found in the Supporting Information. The probed inhibitory mechanism closely resembles the acylation phase of the fully functional M^pro^ described in Scheme 1A. The presence of (PhSe)_2_ does not prevent C145 activation, which is deprotonated by H41 with an activation energy of 5.28 kcal mol^–1^; a zwitterionic intermediate formed by deprotonated cysteine (C) and protonated histidine (HIP) was found on the potential energy surface (PES) at 6.33 kcal mol^–1^ above the initial RC. This point is higher than the transition state leading to it on the Gibbs free energy surface, but it is correctly located at slightly lower energy on the electronic energy surface. This is thus considered an artifact of the thermodynamic correction and is common in those cases in which the transition state and the zwitterion are close in energy on the electronic PES. After proton transfer from C145 to H41, the activated cysteinate residue attacks the weak electrophilic Se atom of (PhSe)_2_, resulting in a three-center intermediate (TCI) with an almost linear S–Se–Se arrangement of the 2.46 Å C(S)–Se bond and 2.81 Å Se–Se (PhSe)_2_ bond. This step occurs without an appreciable activation energy, only requiring a slight conformational rearrangement of (PhSe)_2_ within the catalytic pocket. The TCI is located at 1.71 kcal mol^–1^ above the initial RC, and the overall step is thus weakly endergonic. Further breaking of the Se–Se bond was found to occur also without an appreciable activation energy, leading to a free selenolate and to fully formed S–Se bonds. This adduct (PInhb) lies at −0.95 kcal mol^–1^ with respect to the initial RC, and the overall inhibition process is thus very weakly exergonic/isoergonic. Further protonation of PhSe^–^ via back-proton transfer from HIP leads to higher-energy products (product complex, PC), located at 3.75 kcal mol^–1^ above the RC. Thus, no attempt to locate the transition state for such a process was pursued. For the snapshot at 88 ns, the transition state for this process was associated with high activation energy (10.7 kcal mol^–1^, Figure S4), further suggesting that this process likely does not occur.

The energetics here provided does not suggest a strong covalent inhibition, with the overall process being only very weakly favored from the thermodynamic point of view. However, at least partly, (PhSe)_2_ is expected to bind to Cys145. The energetics can still be safely compared to the one computed for the covalent binding of ebselen to M^pro^ provided by some of us.^6^ For this latter organoselenium compound, the inhibited product was located at ca. −7 kcal mol^–1^ with respect to the RC. Thus, our calculations predict a lower (covalent) inhibition strength of (PhSe)_2_ compared to ebselen, in nice agreement with the experimental findings.

## 4 Conclusions

In this work, starting from the *in vitro* effects of (PhSe)_2_ on SARS-CoV-2 replication, using Vero E6 cells, we investigated one of the possible molecular aspects of inhibition using MD simulations and quantum mechanics (DFT) calculations. Experimental findings show an inhibitory concentration in the low micromolar range. The adduct between M^pro^/PL^pro^ and (PhSe)_2_ is stable in the simulation time, with (PhSe)_2_ phenyl rings buried between histidine residues and residues Met165, Met49, and Cys145 via π-stacking interactions between the imidazole ring of histidine and the phenyl ring of (PhSe)_2_, as confirmed by examining the key dihedral angle Ψ (C–Se–Se–C′) and supporting dihedrals θ1 and θ2 of (PhSe)_2_. From the mechanistic point of view, we found that the covalent S–Se bond formation is only slightly energetically favored, in agreement with the fact that (PhSe)_2_ appears to be a less effective covalent inhibitor than the well-known ebselen, for which a thermodynamically favored S–Se bond formation was previously reported.^6,13^

Further computational and experimental work is required to design (PhSe)_2_ derivatives and new molecules with organodiselenide scaffolds as inhibitors of viral proteases, offering an appealing strategy for combating SARS-CoV-2.

## Supporting Information Available

Force field parameters for (PhSe)_2_, PES scans [B3LYP/6-311G(d,p),ccPVTZ], radius of gyration plots, RMDS plots, equilibration plots, MM/GBSA energy results, total MM/GBSA and MM/PBSA energy decomposition results, reaction energies [SMD-B3LYP-D3(BJ)/6-311G(d,p),ccPVTZ] for (PhSe)_2_ + Cys in the neutral and anionic form, and coordinates of the QM structures (PDF).

## Notes

AMBER 2020 software [the Amber molecular dynamics Package (ambermd.org)] was used to perform mocular dynamics simulations; analyses were carried out with CPPTRAJ, a free open-source tool included in AmberTools 20. The DFT calculations were performed using Gaussian 16 Rev. A.03 (Gaussian 16|Gaussian.com). The protein–ligand interaction and docking analyses were performed using Discovery Studio Visualizer v21.1.0.20298, VMD for LINUXAMD64, version 1.9.4a57 (April 27, 2022), AutoDockTools-1.5.6, and AutoDock Vina- 1.1.2, which can be downloaded for free at https://discover.3ds.com/discovery-studio-visualizer-download, http://www.ks.uiuc.edu/Research/vmd/, https://ccsb.scripps.edu/mgltools/,and https://vina.scripps.edu,respectively. The computational protocols are described in the Materials and Methods. The coordinates of all optimized structures are provided in the Supporting Information
