# Supplementary material for: Diphenyl Diselenide and SARS-CoV-2: in silico Exploration of the Mechanisms of Inhibition of Main Protease (Mpro) and Papain-like Protease (PLpro)
Source: J Chem Inf Model. 2023 Mar 23;63(7):2226–39. doi: 10.1021/acs.jcim.3c00168 (PMC10091420; doi:10.1021/acs.jcim.3c00168)
Supplement: Supplementary file 1 — ci3c00168_si_001.pdf [file ci3c00168_si_001.pdf]

Supporting Information

Diphenyl diselenide and SARS-CoV-2: *in silico* exploration of the mechanisms of inhibition of Main protease (M^{pro}) and Papain-like protease (PL^{pro})

Folorunsho Bright Omage[1], Andrea Madabeni[2], Amanda Resende Tucci[3,4], Pablo Andrei Nogara[1], Marco Bortoli[5], Alice dos Santos Rosa[3,4], Vivian Neuza dos Santos Ferreira[3,4], Joao Batista Teixeira Rocha[1], Milene Dias Miranda[3,4], * Laura Orian[2]*

[1] Departamento de Bioquímica e Biologia Molecular, Universidade Federal de Santa Maria, Santa Maria RS 97105-900, Brazil.

[2] Dipartimento di Scienze Chimiche, Università degli Studi di Padova, Via Marzolo 1, 35131 Padova, Italy

[3] Laboratório de Vírus Respiratórios e do Sarampo, Instituto Oswaldo Cruz, Fundação Oswaldo Cruz RJ 21041-210, Brazil

[4] Laboratório de Morfologia e Morfogênese Viral, Instituto Oswaldo Cruz, Fundação Oswaldo Cruz RJ 21041-210, Brazil

[5]Institute of Computational Chemistry and Catalysis (IQCC) and Department of Chemistry, Faculty of Sciences, University of Girona, C/M. A. Capmany 69, 17003 Girona, Spain.

*Correspondence: laura.orian@unipd.it (L.O.); mmiranda@ioc.fiocruz.br (M.D.M.)

Table S1 Force field parameters for $(PhSe)_2$ taken from *J. Phys. Chem. A* **2016**, 120, 25, 4389–4400. Values are reported in AMBER unit of measure (see https://ambermd.org/FileFormats.php#parm.dat for a full specification of the different terms).

bonds		angles		dihedrals					
r_{eq}	RK	θ_{eq}	TK	IDIVF	$PK_1/2$	$PK_2/2$	$PK_3/2$	$PK_4/2$	PHASE
Se-Se		Se-Se-C		C-Se-Se-C					
2.33	109	104.53	67.14	1	-1.8498	-1.6208	−0.6648	0.0015	180
Se-C		Se-C-C		Se-Se-C-C					
1.948	143.4	124.14	86.16	1	2.5629	0.2734	0.8091	0.0679	180
C-C		C-C-C		Se/C-C-C-C					
1.387	478.4	119.97	67.18	Se/C/H-C-C-H					
C-H		C-C-C		4			14.5		180
1.087	344.3	120.01	48.46						

Table S2: PES Scan. Bond distances of the chalcogens, constrained in the TCI as starting structure for TS guess search for the S-Se bond formation and the Se-Se bond breaking. Level of Theory: B3LYP/6-311G(d,p),ccPVTZ

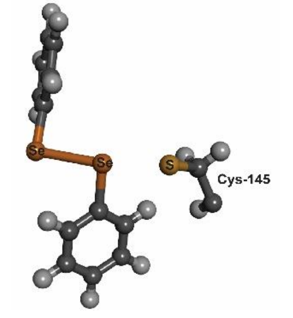

	TCI	1	2	3	4	5	6	7	8	9	10	11	12	13	14	15	16	17	18	19	20	21	22	23	24	25
S-Se	2.615	2.715	2.815	2.915	3.015	3.115	3.215	3.315	3.415	3.515	3.615															
Se-Se	2.686	2.790	2.890	2.990	3.090	3.190	3.290	3.390	3.490	3.590	3.690	3.790	3.890	3.990	4.090	4.190	4.290	4.390	4.490	4.590	4.690	4.790	4.890	4.990	5.090	5.190

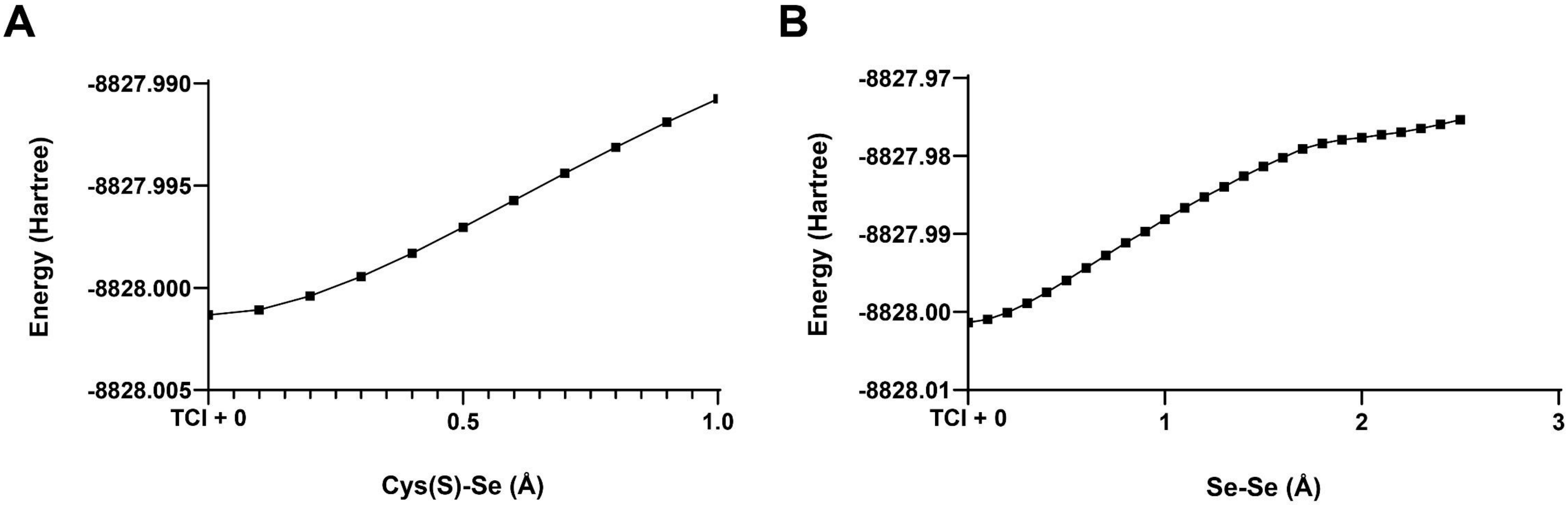

Figure S1. Plot of PES scan starting from TCI coordinate. (A) The TCI S-Se bond distance of 2.61 Å extended at 0.1 Å in 10 steps (B) TCI Se-Se bond distance of 2.69 Å extended at 0.1 Å in 25 steps. Level of Theory: B3LYP/6-311G(d,p),ccPVTZ

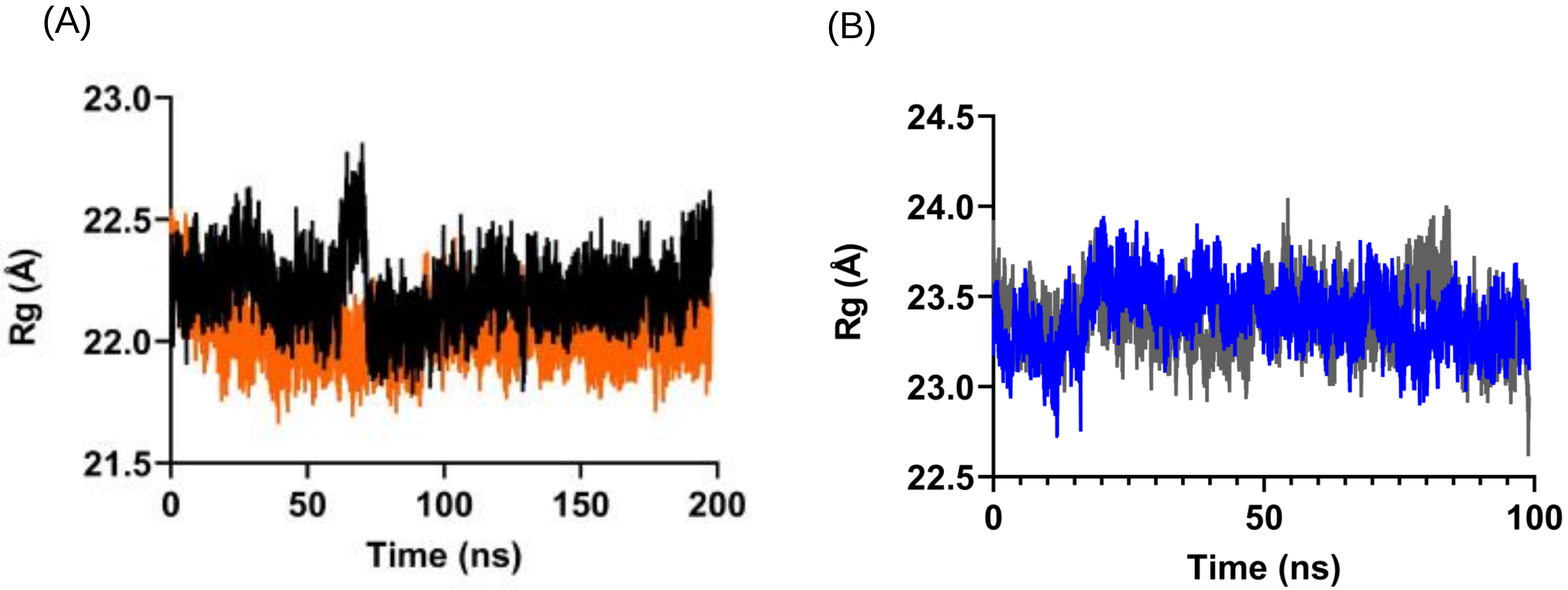

Figure S2. (A) Radius of Gyration for Mpro Apo (black) and $PhSe_2$-M^{pro}(orange) (B) Radius of gyration for (PhSe)2 bound PLpro (blue) and Apo (grey).

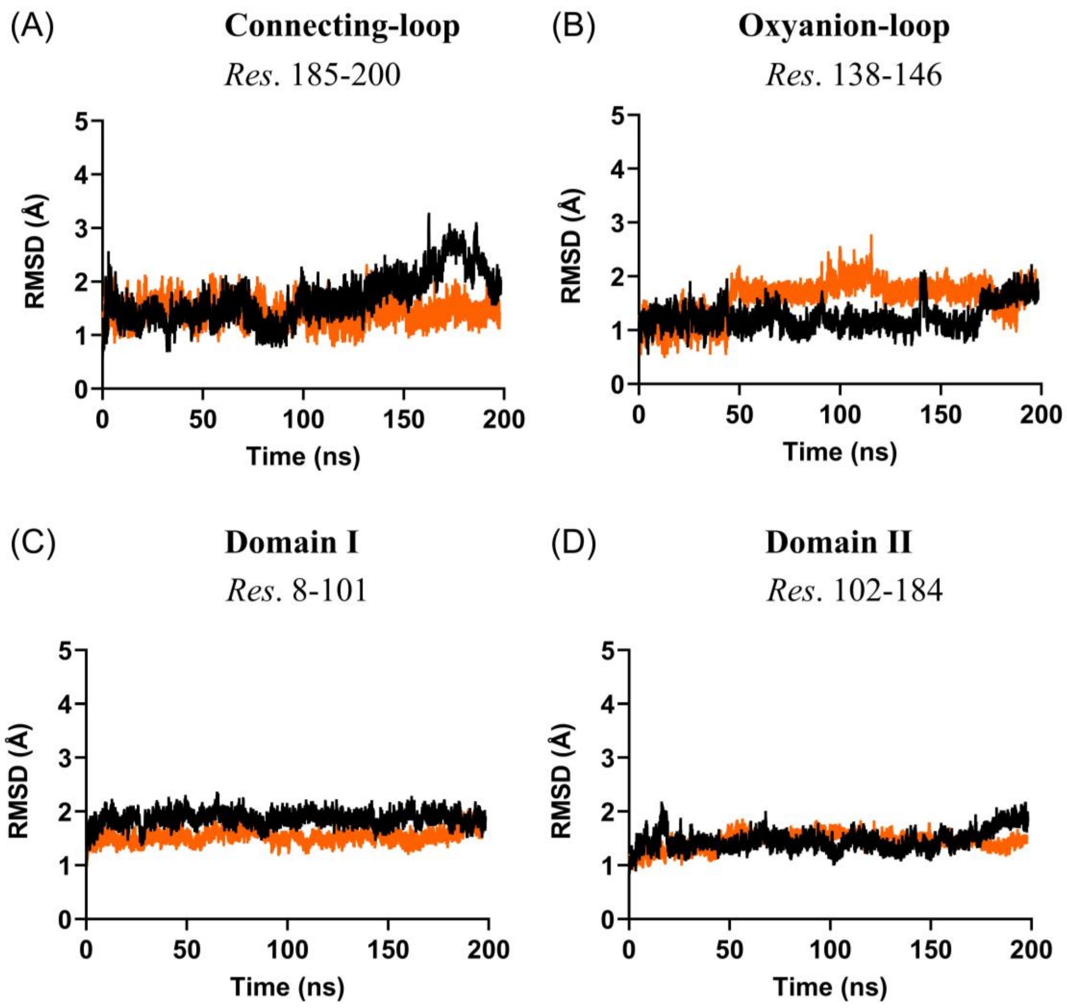

Figure S3. RMSD plots of the (A) Connecting-loop (B) Oxyanion-loop (C) Domain I and (D) Domain II. The average RMSD of $(PhSe)_2$ + M^{pro} is represented in orange and the Apo structure in black.

Table S3: Model reaction for the neutral reaction: $(PhSe)_2$ + H-Cys —> PhSe-SCys + PhSeH. Level of theory: SMD-B3LYP-D3(BJ)/6-311G(d,p),ccPVTZ. Δ_{rxn}, in kcalmol^{-1}, is the classical reaction energy. Electronic ($Elect_{298}$) energies and Gibb's ($Gibbs_{298}$) free energies were measured at 298K

**Method**	**Phase**	**Type of Energy**	**E(PhSe)$_2$**	**E(CysS-H)**	**E(PhSe-SCys)**	**E(PhSeH)**	**Δ_{rxn}(kcal mol^{-1})**
B3LYP/6-311G(d,p),ccPVTZ	Water	$Elect_{298}$	-3304903.593	-561162.3242	-2213245.891	-1652820.382	**-0.35**
		$Gibbs_{298}$	-3304817.045	-561072.1257	-2213108.98	-1652779.499	**0.69**
	Diethylether	$Elect_{298}$	-3304908.825	-561160.7837	-2213244.498	-1652822.946	**2.17**
		$Gibbs_{298}$	-3304822.144	-561070.5281	-2213109.409	-1652781.878	**1.38**

Table S4: Model reaction for the anionic reaction: $(PhSe)_2$ + Cys- —> PhSe-SCys + PhSe-. Level of theory: SMD-B3LYP-D3(BJ)/6-311G(d,p),ccPVTZ.Δ_{rxn}, in kcalmol^{-1}, is the classical reaction energy. Electronic ($Elect_{298}$) energies and Gibb's ($Gibbs_{298}$) free energies were measured at 298K

**Method**	**Phase**	**Type of Energy**	**E(PhSe)$_2$**	**E(CysS-)**	**E(PhSe-SCys)**	**E(PhSe-)**	**Δ_{rxn}(kcal mol^{-1})**
B3LYP/6-311G(d,p),ccPVTZ	Water	$Elect_{298}$	-3304903.593	-560858.834	-2213245.891	-1652533.559	**-17.02**
		$Gibbs_{298}$	-3304817.045	-560774.438	-2213108.980	-1652496.810	**-14.31**
	Diethylether	$Elect_{298}$	-3304908.825	-560845.725	-2213244.498	-1652521.834	**-11.78**
		$Gibbs_{298}$	-3304822.144	-560761.086	-2213109.409	-1652485.355	**-11.53**

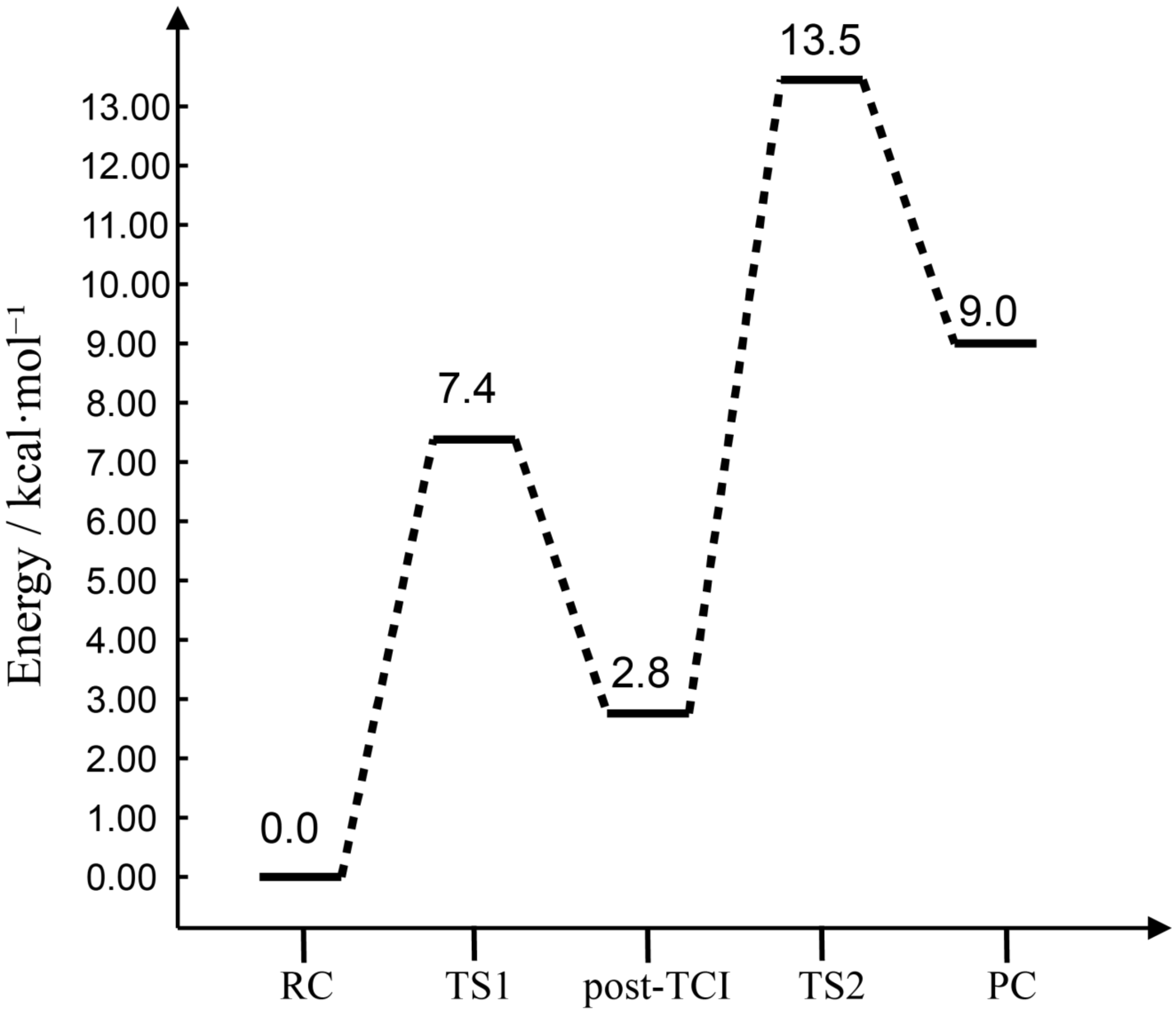

Figure S4. Energy profile for the inhibition mechanism of M^{pro} by $(PhSe)_2$ optimized in water (black) starting from the snapshot at 88 ns. All energies (G) are relative to RC. Level of theory: SMD-B3LYP-D3(BJ)/6-311G(d,p),cc-pVTZ.

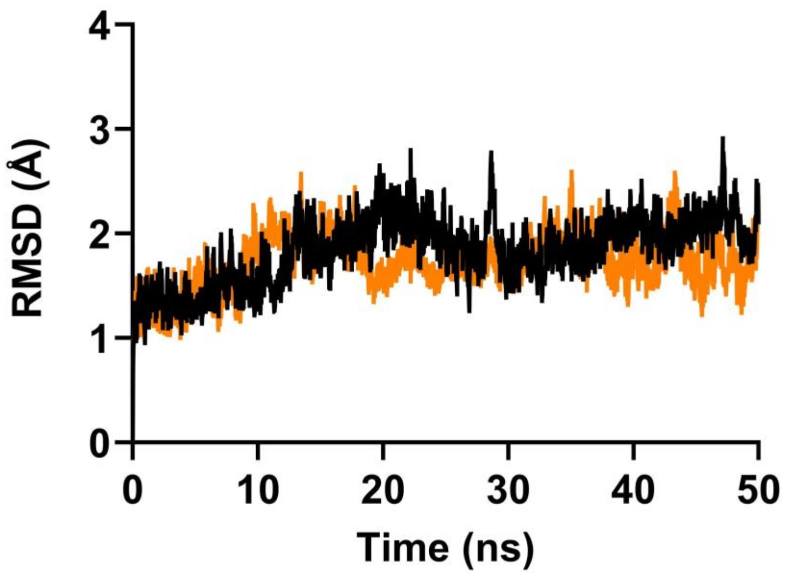

Figure S5. The equilibration RMSD plots of $(PhSe)_2$ + M^{pro} represented in orange and the Apo structure in black. Simulation time 50 ns

Table S5: The calculated binding free energies using MM/GBSA method for M^{pro} DPDS complex.

Energy (Kcal/mol)	GBSA	
	Mean	Std

$\Delta Evdw$	-24.51	4.15
$\Delta Eele$	-3.29	2.27
$\Delta Gpol$ [EGB]	11.34	2.58
$\Delta Gnonpol$ [ESURF]	-3.18	0.52
$\Delta Gbind$	-19.64	3.76

Table S6: The calculated binding free energies using MM/GBSA method for PL^{pro} DPDS complex.

Energy (Kcal/mol)	GBSA	
	Mean	Std
$\Delta Evdw$	-29.30	1.86
$\Delta Eele$	-4.72	2.04
$\Delta Gpol$ [EGB]	14.36	1.59
$\Delta Gnonpol$ [ESURF]	-3.89	0.18
$\Delta Gbind$	-23.55	1.93

Table S7: Total Energy Decomposition using MM/GBSA method for M^{pro} $(PhSe)_2$ complex.

Total Energy Decomposition:

Residue	Internal Avg.	van der Waals Avg.	Electrostatic Avg.	Polar Solvation Avg.	Non-Polar Solv. Avg.	TOTAL
SER 1	0.00	0.00	0.01	-0.01	0.00	0.00
GLY 2	0.00	0.00	0.00	0.00	0.00	0.00
PHE 3	0.00	0.00	0.00	0.00	0.00	0.00
ARG 4	0.00	0.00	0.01	-0.01	0.00	0.00
LYS 5	0.00	0.00	0.02	-0.02	0.00	0.00
MET 6	0.00	0.00	0.00	0.00	0.00	0.00
ALA 7	0.00	0.00	0.00	0.00	0.00	0.00
PHE 8	0.00	0.00	0.00	0.00	0.00	0.00
PRO 9	0.00	0.00	0.00	0.00	0.00	0.00
SER 10	0.00	0.00	0.00	0.00	0.00	0.00
GLY 11	0.00	0.00	0.00	0.00	0.00	0.00
LYS 12	0.00	0.00	0.01	-0.01	0.00	0.00
VAL 13	0.00	0.00	0.00	0.00	0.00	0.00
GLU 14	0.00	0.00	-0.02	0.02	0.00	0.00
GLY 15	0.00	0.00	0.00	0.00	0.00	0.00
CYS 16	0.00	0.00	0.00	0.00	0.00	0.00
MET 17	0.00	0.00	0.00	0.00	0.00	0.00
VAL 18	0.00	0.00	0.00	0.00	0.00	0.00
GLN 19	0.00	0.00	0.00	0.01	0.00	0.01
VAL 20	0.00	0.00	0.00	0.00	0.00	0.00
THR 21	0.00	0.00	0.00	0.01	0.00	0.00
CYS 22	0.00	-0.01	0.00	0.00	0.00	0.00
GLY 23	0.00	0.00	0.00	0.01	0.00	0.00
THR 24	0.00	-0.02	0.00	0.03	0.00	0.00
THR 25	0.00	-0.11	-0.01	0.05	-0.02	-0.10
THR 26	0.00	-0.02	0.00	0.02	0.00	0.00
LEU 27	0.00	-0.06	0.00	0.01	0.00	-0.05
ASN 28	0.00	0.00	0.00	0.00	0.00	0.00
GLY 29	0.00	0.00	0.00	0.00	0.00	0.00

LEU 30	0.00	0.00	0.00	0.00	0.00	0.00
TRP 31	0.00	0.00	0.00	0.00	0.00	0.00
LEU 32	0.00	0.00	0.00	0.00	0.00	0.00
ASP 33	0.00	0.00	-0.01	0.01	0.00	0.00
ASP 34	0.00	0.00	0.00	0.00	0.00	0.00
VAL 35	0.00	0.00	0.00	0.00	0.00	0.00
VAL 36	0.00	0.00	0.00	0.00	0.00	0.00
TYR 37	0.00	0.00	0.00	0.00	0.00	0.00
CYS 38	0.00	-0.01	0.01	-0.01	0.00	-0.01
PRO 39	0.00	-0.04	0.02	-0.03	0.00	-0.05
ARG 40	0.00	-0.08	-0.05	0.00	0.00	-0.13
HID 41	0.00	-1.29	-0.03	0.61	-0.16	-0.86
VAL 42	0.00	-0.06	0.01	0.01	0.00	-0.04
ILE 43	0.00	-0.06	0.00	0.00	0.00	-0.06
CYS 44	0.00	-0.50	-0.10	0.29	-0.06	-0.38
THR 45	0.00	-0.23	0.03	0.06	-0.01	-0.15
SER 46	0.00	-0.32	-0.02	0.19	-0.06	-0.21
GLU 47	0.00	-0.05	0.07	-0.01	0.00	0.01
ASP 48	0.00	-0.11	0.04	0.01	0.00	-0.06
MET 49	0.00	-1.36	-0.13	0.46	-0.25	-1.27
LEU 50	0.00	-0.24	0.01	0.06	-0.03	-0.21
ASN 51	0.00	-0.05	-0.03	0.09	0.00	0.00
PRO 52	0.00	-0.02	0.00	0.01	0.00	-0.01
ASN 53	0.00	-0.02	-0.01	0.03	0.00	0.01
TYR 54	0.00	-0.49	-0.02	0.20	-0.03	-0.34
GLU 55	0.00	-0.03	0.01	0.06	0.00	0.04
ASP 56	0.00	0.00	0.02	-0.01	0.00	0.01
LEU 57	0.00	-0.03	0.00	0.00	0.00	-0.03
LEU 58	0.00	-0.01	0.00	0.00	0.00	0.00
ILE 59	0.00	0.00	0.00	0.00	0.00	0.00
ARG 60	0.00	0.00	-0.02	0.03	0.00	0.01
LYS 61	0.00	-0.01	-0.05	0.09	0.00	0.04
SER 62	0.00	0.00	0.00	0.00	0.00	0.00

ASN 63	0.00	0.00	0.00	0.00	0.00	0.00
HIE 64	0.00	0.00	0.00	0.00	0.00	0.00
ASN 65	0.00	0.00	0.00	0.01	0.00	0.00
PHE 66	0.00	0.00	0.00	0.00	0.00	0.00
LEU 67	0.00	0.00	0.00	0.00	0.00	0.00
VAL 68	0.00	0.00	0.00	0.00	0.00	0.00
GLN 69	0.00	0.00	0.00	0.00	0.00	0.00
ALA 70	0.00	0.00	0.00	0.00	0.00	0.00
GLY 71	0.00	0.00	0.00	0.00	0.00	0.00
ASN 72	0.00	0.00	0.00	0.00	0.00	0.00
VAL 73	0.00	0.00	0.00	0.00	0.00	0.00
GLN 74	0.00	0.00	0.00	0.00	0.00	0.00
LEU 75	0.00	0.00	0.00	0.00	0.00	0.00
ARG 76	0.00	0.00	0.00	0.00	0.00	0.00
VAL 77	0.00	0.00	0.00	0.00	0.00	0.00
ILE 78	0.00	0.00	0.00	0.00	0.00	0.00
GLY 79	0.00	0.00	0.00	0.00	0.00	0.00
HIE 80	0.00	0.00	0.00	0.01	0.00	0.00
SER 81	0.00	0.00	0.00	0.00	0.00	0.00
MET 82	0.00	0.00	0.00	0.00	0.00	0.00
GLN 83	0.00	0.00	0.00	0.01	0.00	0.00
ASN 84	0.00	0.00	0.01	0.00	0.00	0.00
CYS 85	0.00	-0.03	-0.01	0.02	0.00	-0.02
VAL 86	0.00	-0.01	0.00	0.00	0.00	0.00
LEU 87	0.00	-0.01	0.00	0.00	0.00	0.00
LYS 88	0.00	0.00	0.00	0.00	0.00	0.00
LEU 89	0.00	0.00	0.00	0.00	0.00	0.00
LYS 90	0.00	0.00	0.00	0.00	0.00	0.00
VAL 91	0.00	0.00	0.00	0.00	0.00	0.00
ASP 92	0.00	0.00	0.00	0.00	0.00	0.00
THR 93	0.00	0.00	0.00	0.00	0.00	0.00
ALA 94	0.00	0.00	0.00	0.00	0.00	0.00
ASN 95	0.00	0.00	0.00	0.00	0.00	0.00

PRO 96	0.00	0.00	0.00	0.00	0.00	0.00
LYS 97	0.00	0.00	0.01	-0.01	0.00	0.00
THR 98	0.00	0.00	0.00	0.00	0.00	0.00
PRO 99	0.00	0.00	0.00	0.00	0.00	0.00
LYS 100	0.00	0.00	0.01	-0.01	0.00	0.00
TYR 101	0.00	0.00	0.00	0.00	0.00	0.00
LYS 102	0.00	0.00	0.01	-0.01	0.00	0.00
PHE 103	0.00	0.00	0.00	0.00	0.00	0.00
VAL 104	0.00	0.00	0.00	0.00	0.00	0.00
ARG 105	0.00	0.00	0.02	-0.02	0.00	0.01
ILE 106	0.00	0.00	0.00	0.00	0.00	0.00
GLN 107	0.00	0.00	0.00	0.00	0.00	0.00
PRO 108	0.00	0.00	0.00	0.00	0.00	0.00
GLY 109	0.00	0.00	0.00	0.00	0.00	0.00
GLN 110	0.00	0.00	0.00	0.00	0.00	0.00
THR 111	0.00	0.00	0.00	0.00	0.00	0.00
PHE 112	0.00	0.00	0.00	0.00	0.00	0.00
SER 113	0.00	0.00	0.00	0.00	0.00	0.00
VAL 114	0.00	0.00	0.00	0.00	0.00	0.00
LEU 115	0.00	0.00	0.00	0.00	0.00	0.00
ALA 116	0.00	0.00	0.00	0.00	0.00	0.00
CYS 117	0.00	0.00	0.00	0.00	0.00	0.00
TYR 118	0.00	0.00	0.00	0.01	0.00	0.00
ASN 119	0.00	0.00	0.00	0.01	0.00	0.01
GLY 120	0.00	0.00	0.00	0.00	0.00	0.00
SER 121	0.00	0.00	0.00	0.00	0.00	0.00
PRO 122	0.00	0.00	0.00	0.00	0.00	0.00
SER 123	0.00	0.00	0.00	0.00	0.00	0.00
GLY 124	0.00	0.00	0.00	0.00	0.00	0.00
VAL 125	0.00	0.00	0.00	0.00	0.00	0.00
TYR 126	0.00	0.00	0.00	0.00	0.00	0.00
GLN 127	0.00	0.00	0.00	0.00	0.00	0.00
CYS 128	0.00	0.00	0.00	0.00	0.00	0.00

ALA 129	0.00	0.00	0.00	0.00	0.00	0.00
MET 130	0.00	0.00	0.00	0.00	0.00	0.00
ARG 131	0.00	0.00	0.02	-0.02	0.00	0.00
PRO 132	0.00	0.00	0.00	0.00	0.00	0.00
ASN 133	0.00	0.00	0.00	0.01	0.00	0.00
PHE 134	0.00	0.00	0.00	0.01	0.00	0.00
THR 135	0.00	0.00	0.00	0.00	0.00	0.00
ILE 136	0.00	0.00	0.00	0.00	0.00	0.00
LYS 137	0.00	0.00	0.02	-0.02	0.00	0.00
GLY 138	0.00	0.00	0.00	0.00	0.00	0.00
SER 139	0.00	0.00	0.00	0.00	0.00	0.00
PHE 140	0.00	0.00	0.00	0.01	0.00	0.00
LEU 141	0.00	0.00	0.00	0.01	0.00	0.00
ASN 142	0.00	-0.04	0.00	0.06	-0.01	0.01
GLY 143	0.00	-0.02	0.00	0.01	0.00	-0.01
SER 144	0.00	-0.01	0.00	0.00	0.00	-0.01
CYS 145	0.00	-0.08	0.03	-0.01	-0.01	-0.07
GLY 146	0.00	-0.01	0.01	0.01	0.00	0.01
SER 147	0.00	0.00	0.00	0.01	0.00	0.00
VAL 148	0.00	0.00	0.00	0.00	0.00	0.00
GLY 149	0.00	0.00	0.00	0.00	0.00	0.00
PHE 150	0.00	0.00	0.00	0.00	0.00	0.00
ASN 151	0.00	0.00	0.00	0.00	0.00	0.00
ILE 152	0.00	0.00	0.00	0.00	0.00	0.00
ASP 153	0.00	0.00	-0.01	0.01	0.00	0.00
TYR 154	0.00	0.00	0.00	0.00	0.00	0.00
ASP 155	0.00	0.00	-0.01	0.01	0.00	0.00
CYS 156	0.00	0.00	0.00	0.00	0.00	0.00
VAL 157	0.00	0.00	0.00	0.00	0.00	0.00
SER 158	0.00	0.00	0.00	0.00	0.00	0.00
PHE 159	0.00	0.00	0.00	0.00	0.00	0.00
CYS 160	0.00	0.00	0.00	0.00	0.00	0.00
TYR 161	0.00	0.00	0.00	0.00	0.00	0.00

MET 162	0.00	-0.01	-0.01	0.01	0.00	-0.01
HIE 163	0.00	-0.03	-0.01	0.02	0.00	-0.02
HIE 164	0.00	-0.37	-0.07	0.41	-0.03	-0.05
MET 165	0.00	-0.87	-0.19	0.30	-0.16	-0.91
GLU 166	0.00	-0.16	-0.06	0.24	-0.03	0.00
LEU 167	0.00	-0.13	0.02	0.02	-0.01	-0.10
PRO 168	0.00	-0.07	0.01	0.01	-0.02	-0.07
THR 169	0.00	-0.01	0.00	0.01	0.00	0.00
GLY 170	0.00	0.00	0.00	0.00	0.00	0.00
VAL 171	0.00	-0.01	0.00	0.00	0.00	-0.01
HIE 172	0.00	-0.01	0.00	0.01	0.00	-0.01
ALA 173	0.00	-0.03	-0.01	0.01	0.00	-0.02
GLY 174	0.00	-0.01	0.01	0.00	0.00	-0.01
THR 175	0.00	-0.01	-0.01	0.01	0.00	-0.01
ASP 176	0.00	0.00	-0.03	0.03	0.00	0.00
LEU 177	0.00	0.00	0.00	0.00	0.00	0.00
GLU 178	0.00	0.00	-0.01	0.01	0.00	0.00
GLY 179	0.00	0.00	0.01	0.00	0.00	0.00
ASN 180	0.00	0.00	0.00	0.01	0.00	0.00
PHE 181	0.00	-0.10	0.02	0.03	0.00	-0.05
TYR 182	0.00	0.00	0.00	0.01	0.00	0.00
GLY 183	0.00	0.00	0.00	0.01	0.00	0.00
PRO 184	0.00	-0.01	0.01	0.00	0.00	0.00
PHE 185	0.00	-0.07	0.02	-0.01	0.00	-0.06
VAL 186	0.00	-0.33	-0.05	0.21	-0.01	-0.17
ASP 187	0.00	-1.05	-0.12	0.42	-0.09	-0.84
ARG 188	0.00	-1.44	-0.50	1.10	-0.12	-0.96
GLN 189	0.00	-1.53	-0.33	0.88	-0.23	-1.22
THR 190	0.00	-0.29	-0.06	0.22	-0.03	-0.15
ALA 191	0.00	-0.05	0.01	0.00	-0.01	-0.04
GLN 192	0.00	-0.13	-0.02	0.12	-0.01	-0.05
ALA 193	0.00	-0.01	0.00	0.01	0.00	0.00
ALA 194	0.00	0.00	0.00	0.00	0.00	0.00

GLY 195	0.00	0.00	0.00	0.00	0.00	0.00
THR 196	0.00	0.00	0.00	0.00	0.00	0.00
ASP 197	0.00	0.00	-0.02	0.02	0.00	0.00
THR 198	0.00	0.00	0.00	0.00	0.00	0.00
THR 199	0.00	0.00	0.00	0.00	0.00	0.00
ILE 200	0.00	0.00	0.00	0.00	0.00	0.00
THR 201	0.00	0.00	0.00	0.00	0.00	0.00
VAL 202	0.00	0.00	0.00	0.00	0.00	0.00
ASN 203	0.00	0.00	0.00	0.00	0.00	0.00
VAL 204	0.00	0.00	0.00	0.00	0.00	0.00
LEU 205	0.00	0.00	0.00	0.00	0.00	0.00
ALA 206	0.00	0.00	0.00	0.00	0.00	0.00
TRP 207	0.00	0.00	0.00	0.00	0.00	0.00
LEU 208	0.00	0.00	0.00	0.00	0.00	0.00
TYR 209	0.00	0.00	0.00	0.00	0.00	0.00
ALA 210	0.00	0.00	0.00	0.00	0.00	0.00
ALA 211	0.00	0.00	0.00	0.00	0.00	0.00
VAL 212	0.00	0.00	0.00	0.00	0.00	0.00
ILE 213	0.00	0.00	0.00	0.00	0.00	0.00
ASN 214	0.00	0.00	0.00	0.00	0.00	0.00
GLY 215	0.00	0.00	0.00	0.00	0.00	0.00
ASP 216	0.00	0.00	-0.01	0.01	0.00	0.00
ARG 217	0.00	0.00	0.00	0.00	0.00	0.00
TRP 218	0.00	0.00	0.00	0.00	0.00	0.00
PHE 219	0.00	0.00	0.00	0.00	0.00	0.00
LEU 220	0.00	0.00	0.00	0.00	0.00	0.00
ASN 221	0.00	0.00	0.00	0.00	0.00	0.00
ARG 222	0.00	0.00	0.00	0.00	0.00	0.00
PHE 223	0.00	0.00	0.00	0.00	0.00	0.00
THR 224	0.00	0.00	0.00	0.00	0.00	0.00
THR 225	0.00	0.00	0.00	0.00	0.00	0.00
THR 226	0.00	0.00	0.00	0.00	0.00	0.00
LEU 227	0.00	0.00	0.00	0.00	0.00	0.00

ASN 228	0.00	0.00	0.00	0.00	0.00	0.00
ASP 229	0.00	0.00	0.00	0.00	0.00	0.00
PHE 230	0.00	0.00	0.00	0.00	0.00	0.00
ASN 231	0.00	0.00	0.00	0.00	0.00	0.00
LEU 232	0.00	0.00	0.00	0.00	0.00	0.00
VAL 233	0.00	0.00	0.00	0.00	0.00	0.00
ALA 234	0.00	0.00	0.00	0.00	0.00	0.00
MET 235	0.00	0.00	0.00	0.00	0.00	0.00
LYS 236	0.00	0.00	0.01	-0.01	0.00	0.00
TYR 237	0.00	0.00	0.00	0.00	0.00	0.00
ASN 238	0.00	0.00	0.00	0.00	0.00	0.00
TYR 239	0.00	0.00	0.00	0.00	0.00	0.00
GLU 240	0.00	0.00	-0.01	0.01	0.00	0.00
PRO 241	0.00	0.00	0.00	0.00	0.00	0.00
LEU 242	0.00	0.00	0.00	0.00	0.00	0.00
THR 243	0.00	0.00	0.00	0.00	0.00	0.00
GLN 244	0.00	0.00	0.00	0.00	0.00	0.00
ASP 245	0.00	0.00	-0.01	0.01	0.00	0.00
HIE 246	0.00	0.00	0.00	0.00	0.00	0.00
VAL 247	0.00	0.00	0.00	0.00	0.00	0.00
ASP 248	0.00	0.00	-0.01	0.01	0.00	0.00
ILE 249	0.00	0.00	0.00	0.00	0.00	0.00
LEU 250	0.00	0.00	0.00	0.00	0.00	0.00
GLY 251	0.00	0.00	0.00	0.00	0.00	0.00
PRO 252	0.00	0.00	0.00	0.00	0.00	0.00
LEU 253	0.00	0.00	0.00	0.00	0.00	0.00
SER 254	0.00	0.00	0.00	0.00	0.00	0.00
ALA 255	0.00	0.00	0.00	0.00	0.00	0.00
GLN 256	0.00	0.00	0.00	0.00	0.00	0.00
THR 257	0.00	0.00	0.00	0.00	0.00	0.00
GLY 258	0.00	0.00	0.00	0.00	0.00	0.00
ILE 259	0.00	0.00	0.00	0.00	0.00	0.00
ALA 260	0.00	0.00	0.00	0.00	0.00	0.00

VAL 261	0.00	0.00	0.00	0.00	0.00	0.00
LEU 262	0.00	0.00	0.00	0.00	0.00	0.00
ASP 263	0.00	0.00	0.00	0.00	0.00	0.00
MET 264	0.00	0.00	0.00	0.00	0.00	0.00
CYS 265	0.00	0.00	0.00	0.00	0.00	0.00
ALA 266	0.00	0.00	0.00	0.00	0.00	0.00
SER 267	0.00	0.00	0.00	0.00	0.00	0.00
LEU 268	0.00	0.00	0.00	0.00	0.00	0.00
LYS 269	0.00	0.00	0.00	0.00	0.00	0.00
GLU 270	0.00	0.00	0.00	0.00	0.00	0.00
LEU 271	0.00	0.00	0.00	0.00	0.00	0.00
LEU 272	0.00	0.00	0.00	0.00	0.00	0.00
GLN 273	0.00	0.00	0.00	0.00	0.00	0.00
ASN 274	0.00	0.00	0.00	0.00	0.00	0.00
GLY 275	0.00	0.00	0.00	0.00	0.00	0.00
MET 276	0.00	0.00	0.00	0.00	0.00	0.00
ASN 277	0.00	0.00	0.00	0.00	0.00	0.00
GLY 278	0.00	0.00	0.00	0.00	0.00	0.00
ARG 279	0.00	0.00	0.01	-0.01	0.00	0.00
THR 280	0.00	0.00	0.00	0.00	0.00	0.00
ILE 281	0.00	0.00	0.00	0.00	0.00	0.00
LEU 282	0.00	0.00	0.00	0.00	0.00	0.00
GLY 283	0.00	0.00	0.00	0.00	0.00	0.00
SER 284	0.00	0.00	0.00	0.00	0.00	0.00
ALA 285	0.00	0.00	0.00	0.00	0.00	0.00
LEU 286	0.00	0.00	0.00	0.00	0.00	0.00
LEU 287	0.00	0.00	0.00	0.00	0.00	0.00
GLU 288	0.00	0.00	-0.01	0.01	0.00	0.00
ASP 289	0.00	0.00	-0.02	0.02	0.00	0.00
GLU 290	0.00	0.00	-0.02	0.02	0.00	0.00
PHE 291	0.00	0.00	0.00	0.00	0.00	0.00
THR 292	0.00	0.00	0.00	0.00	0.00	0.00
PRO 293	0.00	0.00	0.00	0.00	0.00	0.00

PHE 294	0.00	0.00	0.00	0.00	0.00	0.00
ASP 295	0.00	0.00	-0.01	0.01	0.00	0.00
VAL 296	0.00	0.00	0.00	0.00	0.00	0.00
VAL 297	0.00	0.00	0.00	0.00	0.00	0.00
ARG 298	0.00	0.00	0.01	-0.01	0.00	0.00
GLN 299	0.00	0.00	0.00	0.00	0.00	0.00
CYS 300	0.00	0.00	0.00	0.00	0.00	0.00
SER 301	0.00	0.00	0.00	0.00	0.00	0.00
GLY 302	0.00	0.00	0.00	0.00	0.00	0.00
VAL 303	0.00	0.00	0.00	0.00	0.00	0.00
THR 304	0.00	0.00	0.00	0.00	0.00	0.00
PHE 305	0.00	0.00	0.00	0.00	0.00	0.00
GLN 306	0.00	0.00	-0.01	0.01	0.00	0.00

Table S8: Total Energy Decomposition using MM/GBSA method for PL^{pro} $(PhSe)_2$ complex.

Total Energy Decomposition:

Residue	Internal Avg.	van der Waals Avg.	Electrostatic Avg.	Polar Solvation Avg.	Non-Polar Solv. Avg.	TOTAL
THR 1	0.00	0.00	0.03	-0.03	0.00	0.00
ILE 2	0.00	0.00	0.00	0.00	0.00	0.00
LYS 3	0.00	0.00	0.03	-0.03	0.00	0.00
VAL 4	0.00	0.00	0.00	0.00	0.00	0.00

PHE 5	0.00	0.00	0.00	0.00	0.00	0.00
THR 6	0.00	0.00	0.00	0.00	0.00	0.00
THR 7	0.00	0.00	0.00	0.00	0.00	0.00
VAL 8	0.00	0.00	0.00	0.00	0.00	0.00
ASP 9	0.00	0.00	-0.05	0.05	0.00	0.00
ASN 10	0.00	0.00	0.00	0.00	0.00	0.00
ILE 11	0.00	0.00	0.00	0.00	0.00	0.00
ASN 12	0.00	0.00	0.00	0.00	0.00	0.00
LEU 13	0.00	0.00	0.00	0.00	0.00	0.00
HIE 14	0.00	0.00	0.00	0.00	0.00	0.00
THR 15	0.00	0.00	0.00	0.00	0.00	0.00
GLN 16	0.00	0.00	0.00	0.00	0.00	0.00
VAL 17	0.00	0.00	0.00	0.00	0.00	0.00
VAL 18	0.00	0.00	0.00	0.00	0.00	0.00
ASP 19	0.00	0.00	-0.03	0.03	0.00	0.00
MET 20	0.00	0.00	0.00	0.00	0.00	0.00
SER 21	0.00	0.00	0.00	0.00	0.00	0.00
MET 22	0.00	0.00	0.00	0.00	0.00	0.00
THR 23	0.00	0.00	0.00	0.00	0.00	0.00
TYR 24	0.00	0.00	0.00	0.00	0.00	0.00
GLY 25	0.00	0.00	0.00	0.00	0.00	0.00
GLN 26	0.00	0.00	0.00	0.00	0.00	0.00
GLN 27	0.00	0.00	0.00	0.00	0.00	0.00
PHE 28	0.00	0.00	0.00	0.00	0.00	0.00
GLY 29	0.00	0.00	0.00	0.00	0.00	0.00
PRO 30	0.00	0.00	0.00	0.00	0.00	0.00
THR 31	0.00	0.00	0.00	0.00	0.00	0.00
TYR 32	0.00	0.00	0.00	0.00	0.00	0.00
LEU 33	0.00	0.00	0.00	0.00	0.00	0.00
ASP 34	0.00	0.00	-0.07	0.07	0.00	0.00
GLY 35	0.00	0.00	0.00	0.00	0.00	0.00

ALA 36	0.00	0.00	0.00	0.00	0.00	0.00
ASP 37	0.00	0.00	-0.07	0.07	0.00	0.00
VAL 38	0.00	0.00	0.00	0.00	0.00	0.00
THR 39	0.00	0.00	0.00	0.00	0.00	0.00
LYS 40	0.00	0.00	0.06	-0.06	0.00	0.00
ILE 41	0.00	0.00	0.00	0.00	0.00	0.00
LYS 42	0.00	0.00	0.03	-0.03	0.00	0.00
PRO 43	0.00	0.00	0.00	0.00	0.00	0.00
HIE 44	0.00	0.00	0.00	0.00	0.00	0.00
ASN 45	0.00	0.00	0.00	0.00	0.00	0.00
SER 46	0.00	0.00	0.00	0.00	0.00	0.00
HIE 47	0.00	0.00	0.00	0.00	0.00	0.00
GLU 48	0.00	0.00	-0.03	0.03	0.00	0.00
GLY 49	0.00	0.00	0.00	0.00	0.00	0.00
LYS 50	0.00	0.00	0.04	-0.04	0.00	0.00
THR 51	0.00	0.00	0.00	0.00	0.00	0.00
PHE 52	0.00	0.00	0.00	0.00	0.00	0.00
TYR 53	0.00	0.00	0.00	0.00	0.00	0.00
VAL 54	0.00	0.00	0.00	0.00	0.00	0.00
LEU 55	0.00	0.00	0.00	0.00	0.00	0.00
PRO 56	0.00	0.00	0.00	0.00	0.00	0.00
ASN 57	0.00	0.00	0.00	0.00	0.00	0.00
ASP 58	0.00	0.00	-0.04	0.04	0.00	0.00
ASP 59	0.00	0.00	-0.04	0.04	0.00	0.00
THR 60	0.00	0.00	0.00	0.00	0.00	0.00
LEU 61	0.00	0.00	0.00	0.00	0.00	0.00
ARG 62	0.00	0.00	0.05	-0.05	0.00	0.00
VAL 63	0.00	0.00	0.00	0.00	0.00	0.00
GLU 64	0.00	0.00	-0.04	0.04	0.00	0.00
ALA 65	0.00	0.00	0.00	0.00	0.00	0.00
PHE 66	0.00	0.00	0.00	0.00	0.00	0.00

GLU 67	0.00	0.00	-0.04	0.04	0.00	0.00
TYR 68	0.00	0.00	0.00	0.00	0.00	0.00
TYR 69	0.00	0.00	0.00	0.00	0.00	0.00
HIE 70	0.00	0.00	0.00	0.00	0.00	0.00
THR 71	0.00	0.00	0.00	0.00	0.00	0.00
THR 72	0.00	0.00	0.00	0.00	0.00	0.00
ASP 73	0.00	0.00	-0.10	0.10	0.00	0.00
PRO 74	0.00	0.00	0.00	0.00	0.00	0.00
SER 75	0.00	0.00	0.00	0.00	0.00	0.00
PHE 76	0.00	0.00	0.00	0.00	0.00	0.00
LEU 77	0.00	0.00	0.00	0.00	0.00	0.00
GLY 78	0.00	0.00	0.00	0.00	0.00	0.00
ARG 79	0.00	0.00	0.14	-0.13	0.00	0.01
TYR 80	0.00	0.00	-0.01	0.01	0.00	0.00
MET 81	0.00	0.00	0.00	0.00	0.00	0.00
SER 82	0.00	0.00	0.00	0.00	0.00	0.00
ALA 83	0.00	0.00	0.00	0.00	0.00	0.00
LEU 84	0.00	0.00	0.00	0.00	0.00	0.00
ASN 85	0.00	0.00	0.00	0.00	0.00	0.00
HIE 86	0.00	0.00	0.01	0.00	0.00	0.00
THR 87	0.00	0.00	0.01	0.00	0.00	0.00
LYS 88	0.00	0.00	0.09	-0.08	0.00	0.00
LYS 89	0.00	0.00	0.12	-0.11	0.00	0.00
TRP 90	0.00	-0.01	0.01	0.00	0.00	0.00
LYS 91	0.00	0.00	0.08	-0.07	0.00	0.00
TYR 92	0.00	0.00	0.00	0.00	0.00	0.00
PRO 93	0.00	0.00	0.00	0.00	0.00	0.00
GLN 94	0.00	0.00	0.00	0.00	0.00	0.00
VAL 95	0.00	0.00	0.00	0.00	0.00	0.00
ASN 96	0.00	0.00	0.00	0.00	0.00	0.00
GLY 97	0.00	0.00	0.00	0.00	0.00	0.00

LEU 98	0.00	0.00	0.00	0.00	0.00	0.00
THR 99	0.00	0.00	0.00	0.00	0.00	0.00
SER 100	0.00	0.00	0.00	0.01	0.00	0.00
ILE 101	0.00	-0.01	0.00	0.00	0.00	0.00
LYS 102	0.00	0.00	0.09	-0.09	0.00	0.00
TRP 103	0.00	-0.22	0.07	0.07	-0.03	-0.11
ALA 104	0.00	-0.01	-0.03	0.04	0.00	0.00
ASP 105	0.00	-0.03	-0.26	0.29	0.00	-0.01
ASN 106	0.00	-0.68	-0.57	0.93	-0.05	-0.36
ASN 107	0.00	-0.10	0.01	-0.02	0.00	-0.12
CYS 108	0.00	-0.38	0.11	0.01	-0.04	-0.31
TYR 109	0.00	-0.72	0.18	0.03	-0.02	-0.53
LEU 110	0.00	-0.05	0.07	-0.05	0.00	-0.03
ALA 111	0.00	-0.01	0.02	-0.03	0.00	-0.01
THR 112	0.00	-0.02	0.03	-0.05	0.00	-0.04
ALA 113	0.00	-0.01	0.02	-0.02	0.00	0.00
LEU 114	0.00	0.00	0.01	-0.01	0.00	0.00
LEU 115	0.00	0.00	0.00	0.00	0.00	0.00
THR 116	0.00	0.00	0.01	0.00	0.00	0.00
LEU 117	0.00	0.00	0.00	0.00	0.00	0.00
GLN 118	0.00	0.00	0.00	0.00	0.00	0.00
GLN 119	0.00	0.00	0.00	0.00	0.00	0.00
ILE 120	0.00	0.00	0.00	0.00	0.00	0.00
GLU 121	0.00	0.00	-0.01	0.01	0.00	0.00
LEU 122	0.00	0.00	0.00	0.00	0.00	0.00
LYS 123	0.00	0.00	0.02	-0.02	0.00	0.00
PHE 124	0.00	0.00	0.00	0.00	0.00	0.00
ASN 125	0.00	0.00	0.00	0.00	0.00	0.00
PRO 126	0.00	0.00	0.00	0.00	0.00	0.00
PRO 127	0.00	0.00	0.00	0.00	0.00	0.00
ALA 128	0.00	0.00	0.00	0.00	0.00	0.00

LEU 129	0.00	0.00	0.00	0.00	0.00	0.00
GLN 130	0.00	0.00	0.00	0.00	0.00	0.00
ASP 131	0.00	0.00	-0.05	0.05	0.00	0.00
ALA 132	0.00	0.00	0.00	0.00	0.00	0.00
TYR 133	0.00	0.00	0.00	0.00	0.00	0.00
TYR 134	0.00	0.00	0.00	0.00	0.00	0.00
ARG 135	0.00	0.00	0.06	-0.06	0.00	0.00
ALA 136	0.00	0.00	0.00	0.00	0.00	0.00
ARG 137	0.00	0.00	0.03	-0.03	0.00	0.00
ALA 138	0.00	0.00	0.00	0.00	0.00	0.00
GLY 139	0.00	0.00	0.00	0.00	0.00	0.00
GLU 140	0.00	0.00	-0.07	0.07	0.00	0.00
ALA 141	0.00	0.00	0.00	0.00	0.00	0.00
ALA 142	0.00	0.00	-0.01	0.01	0.00	0.00
ASN 143	0.00	0.00	-0.01	0.01	0.00	0.00
PHE 144	0.00	0.00	-0.01	0.01	0.00	0.00
CYS 145	0.00	0.00	-0.01	0.01	0.00	0.00
ALA 146	0.00	0.00	-0.01	0.01	0.00	0.00
LEU 147	0.00	0.00	-0.01	0.01	0.00	0.00
ILE 148	0.00	-0.01	0.00	0.00	0.00	-0.01
LEU 149	0.00	-0.01	0.00	0.01	0.00	-0.01
ALA 150	0.00	0.00	-0.01	0.01	0.00	0.00
TYR 151	0.00	0.00	0.00	0.00	0.00	0.00
CYS 152	0.00	-0.01	-0.01	0.03	0.00	0.00
ASN 153	0.00	-0.01	-0.01	0.03	0.00	0.01
LYS 154	0.00	-0.16	0.69	-0.30	-0.05	0.18
THR 155	0.00	-0.01	-0.01	0.04	0.00	0.01
VAL 156	0.00	-0.01	0.01	-0.01	0.00	0.00
GLY 157	0.00	-0.03	0.05	-0.02	0.00	-0.01
GLU 158	0.00	-0.17	-0.41	0.56	0.00	-0.03
LEU 159	0.00	-2.76	-0.43	0.67	-0.35	-2.87

GLY 160	0.00	-1.33	0.00	0.99	-0.10	-0.44
ASP 161	0.00	-0.93	0.10	0.17	-0.13	-0.79
VAL 162	0.00	-0.12	-0.10	0.10	0.00	-0.11
ARG 163	0.00	-0.04	-0.31	0.34	0.00	-0.01
GLU 164	0.00	-0.08	-0.38	0.54	-0.01	0.07
THR 165	0.00	-0.03	-0.03	0.04	0.00	-0.02
MET 166	0.00	-0.01	-0.01	0.02	0.00	0.00
SER 167	0.00	0.00	0.01	0.00	0.00	0.01
TYR 168	0.00	-0.01	0.01	0.00	0.00	0.00
LEU 169	0.00	0.00	0.00	0.00	0.00	0.00
PHE 170	0.00	0.00	0.00	0.00	0.00	0.00
GLN 171	0.00	0.00	0.00	0.00	0.00	0.00
HID 172	0.00	0.00	0.00	0.00	0.00	0.00
ALA 173	0.00	0.00	0.00	0.00	0.00	0.00
ASN 174	0.00	0.00	0.00	0.00	0.00	0.00
LEU 175	0.00	0.00	0.00	0.00	0.00	0.00
ASP 176	0.00	0.00	-0.02	0.02	0.00	0.00
SER 177	0.00	0.00	0.00	0.00	0.00	0.00
CYS 178	0.00	0.00	0.00	0.00	0.00	0.00
LYS 179	0.00	0.00	-0.01	0.01	0.00	0.00
ARG 180	0.00	0.00	-0.04	0.04	0.00	0.00
VAL 181	0.00	0.00	0.00	0.00	0.00	0.00
LEU 182	0.00	0.00	0.00	0.00	0.00	0.00
ASN 183	0.00	0.00	0.00	0.00	0.00	0.00
VAL 184	0.00	0.00	0.00	0.00	0.00	0.00
VAL 185	0.00	0.00	0.00	0.00	0.00	0.00
CY1 186	0.00	0.00	0.02	-0.02	0.00	0.00
LYS 187	0.00	0.00	-0.03	0.03	0.00	0.00
THR 188	0.00	0.00	0.00	0.00	0.00	0.00
CY1 189	0.00	0.00	0.02	-0.02	0.00	0.00
GLY 190	0.00	0.00	0.00	0.00	0.00	0.00

GLN 191	0.00	0.00	0.00	0.00	0.00	0.00
GLN 192	0.00	0.00	0.00	0.00	0.00	0.00
GLN 193	0.00	0.00	0.00	0.00	0.00	0.00
THR 194	0.00	0.00	0.00	0.00	0.00	0.00
THR 195	0.00	0.00	0.00	0.00	0.00	0.00
LEU 196	0.00	0.00	0.00	0.00	0.00	0.00
LYS 197	0.00	0.00	0.00	0.00	0.00	0.00
GLY 198	0.00	0.00	0.00	0.00	0.00	0.00
VAL 199	0.00	0.00	0.01	0.00	0.00	0.00
GLU 200	0.00	0.00	0.02	-0.01	0.00	0.00
ALA 201	0.00	0.00	0.00	0.00	0.00	0.00
VAL 202	0.00	0.00	0.00	0.00	0.00	0.00
MET 203	0.00	0.00	-0.01	0.01	0.00	0.00
TYR 204	0.00	0.00	0.00	0.00	0.00	0.00
MET 205	0.00	0.00	-0.01	0.01	0.00	0.00
GLY 206	0.00	0.00	0.00	0.01	0.00	0.00
THR 207	0.00	0.00	0.00	0.01	0.00	0.00
LEU 208	0.00	0.00	-0.01	0.01	0.00	0.00
SER 209	0.00	0.00	0.00	0.00	0.00	0.00
TYR 210	0.00	0.00	0.00	0.00	0.00	0.00
GLU 211	0.00	0.00	0.05	-0.05	0.00	0.00
GLN 212	0.00	0.00	0.00	0.00	0.00	0.00
PHE 213	0.00	0.00	0.00	0.00	0.00	0.00
LYS 214	0.00	0.00	-0.04	0.04	0.00	0.00
LYS 215	0.00	0.00	-0.05	0.05	0.00	0.00
GLY 216	0.00	0.00	0.00	0.00	0.00	0.00
VAL 217	0.00	0.00	0.00	0.00	0.00	0.00
GLN 218	0.00	0.00	0.00	0.00	0.00	0.00
ILE 219	0.00	0.00	0.00	0.00	0.00	0.00
PRO 220	0.00	0.00	0.00	0.00	0.00	0.00
CY1 221	0.00	0.00	0.03	-0.03	0.00	0.00

THR 222	0.00	0.00	0.00	0.00	0.00	0.00
CY1 223	0.00	0.00	0.02	-0.02	0.00	0.00
GLY 224	0.00	0.00	0.00	0.00	0.00	0.00
LYS 225	0.00	0.00	-0.04	0.04	0.00	0.00
GLN 226	0.00	0.00	0.00	0.00	0.00	0.00
ALA 227	0.00	0.00	0.00	0.00	0.00	0.00
THR 228	0.00	0.00	0.00	0.00	0.00	0.00
LYS 229	0.00	0.00	-0.05	0.05	0.00	0.00
TYR 230	0.00	0.00	0.00	0.00	0.00	0.00
LEU 231	0.00	0.00	0.00	0.00	0.00	0.00
VAL 232	0.00	0.00	0.00	0.00	0.00	0.00
GLN 233	0.00	0.00	0.00	0.00	0.00	0.00
GLN 234	0.00	0.00	0.00	0.00	0.00	0.00
GLU 235	0.00	0.00	0.01	-0.01	0.00	0.00
SER 236	0.00	0.00	0.00	0.00	0.00	0.00
PRO 237	0.00	0.00	0.00	0.00	0.00	0.00
PHE 238	0.00	0.00	0.00	0.00	0.00	0.00
VAL 239	0.00	0.00	0.00	0.00	0.00	0.00
MET 240	0.00	0.00	-0.01	0.01	0.00	0.00
MET 241	0.00	0.00	0.00	0.00	0.00	0.00
SER 242	0.00	0.00	-0.03	0.03	0.00	0.00
ALA 243	0.00	0.00	0.02	-0.01	0.00	0.00
PRO 244	0.00	-0.01	-0.04	0.04	0.00	0.00
PRO 245	0.00	-0.02	-0.03	0.03	0.00	-0.02
ALA 246	0.00	0.00	0.01	-0.01	0.00	0.00
GLN 247	0.00	0.00	0.01	-0.01	0.00	0.00
TYR 248	0.00	0.00	0.00	0.00	0.00	0.00
GLU 249	0.00	0.00	0.10	-0.09	0.00	0.00
LEU 250	0.00	0.00	0.00	0.00	0.00	0.00
LYS 251	0.00	0.00	-0.06	0.06	0.00	0.00
HID 252	0.00	0.00	0.00	0.00	0.00	0.00

GLY 253	0.00	0.00	0.00	0.00	0.00	0.00
THR 254	0.00	0.00	0.00	0.00	0.00	0.00
PHE 255	0.00	0.00	0.00	0.00	0.00	0.00
THR 256	0.00	0.00	0.00	0.00	0.00	0.00
CYS 257	0.00	0.00	0.00	0.00	0.00	0.00
ALA 258	0.00	0.00	-0.01	0.01	0.00	0.00
SER 259	0.00	-0.01	-0.02	0.02	0.00	-0.01
GLU 260	0.00	-0.02	0.32	-0.30	0.00	0.01
TYR 261	0.00	-0.85	0.18	0.17	-0.08	-0.57
THR 262	0.00	-0.04	0.04	0.00	0.00	0.00
GLY 263	0.00	-0.03	0.04	0.00	0.00	0.01
ASN 264	0.00	-0.09	0.19	-0.14	0.00	-0.03
TYR 265	0.00	-0.49	-0.20	0.36	-0.05	-0.39
GLN 266	0.00	-1.94	-0.95	1.68	-0.31	-1.52
CYS 267	0.00	-0.54	-0.12	0.23	-0.01	-0.44
GLY 268	0.00	-0.58	-0.67	1.23	-0.11	-0.13
HIE 269	0.00	-0.58	-0.15	0.29	-0.02	-0.46
TYR 270	0.00	-1.33	0.03	0.47	-0.13	-0.97
LYS 271	0.00	-0.02	-0.16	0.21	0.00	0.03
HID 272	0.00	0.00	-0.01	0.01	0.00	0.00
ILE 273	0.00	0.00	0.00	0.00	0.00	0.00
THR 274	0.00	0.00	0.00	0.00	0.00	0.00
SER 275	0.00	0.00	0.00	0.00	0.00	0.00
LYS 276	0.00	0.00	-0.01	0.01	0.00	0.00
GLU 277	0.00	0.00	0.02	-0.02	0.00	0.00
THR 278	0.00	0.00	0.00	0.00	0.00	0.00
LEU 279	0.00	0.00	0.00	0.00	0.00	0.00
TYR 280	0.00	0.00	0.00	0.00	0.00	0.00
CYS 281	0.00	0.00	-0.01	0.01	0.00	0.00
ILE 282	0.00	0.00	0.01	-0.01	0.00	0.00
ASP 283	0.00	-0.01	-0.02	0.07	0.00	0.04

GLY 284	0.00	0.00	0.01	-0.01	0.00	0.00
ALA 285	0.00	0.00	0.01	0.00	0.00	0.00
LEU 286	0.00	0.00	0.00	0.00	0.00	0.00
LEU 287	0.00	0.00	0.00	0.00	0.00	0.00
THR 288	0.00	0.00	0.00	0.00	0.00	0.00
LYS 289	0.00	0.00	-0.02	0.02	0.00	0.00
SER 290	0.00	0.00	0.00	0.00	0.00	0.00
SER 291	0.00	0.00	0.00	0.00	0.00	0.00
GLU 292	0.00	0.00	0.06	-0.06	0.00	0.00
TYR 293	0.00	0.00	-0.01	0.01	0.00	0.00
LYS 294	0.00	0.00	-0.11	0.11	0.00	0.00
GLY 295	0.00	0.00	-0.01	0.01	0.00	0.00
PRO 296	0.00	-0.01	0.03	-0.02	0.00	0.00
ILE 297	0.00	0.00	0.01	-0.01	0.00	0.00
THR 298	0.00	-0.02	0.01	0.00	0.00	-0.01
ASP 299	0.00	-0.01	0.28	-0.25	0.00	0.02
VAL 300	0.00	0.00	0.00	0.00	0.00	0.00
PHE 301	0.00	0.00	0.00	0.00	0.00	0.00
TYR 302	0.00	0.00	0.00	0.00	0.00	0.00
LYS 303	0.00	0.00	-0.01	0.01	0.00	0.00
GLU 304	0.00	0.00	0.03	-0.03	0.00	0.00
ASN 305	0.00	0.00	0.00	0.00	0.00	0.00
SER 306	0.00	0.00	0.00	0.00	0.00	0.00
TYR 307	0.00	0.00	0.00	0.00	0.00	0.00
THR 308	0.00	0.00	0.00	0.00	0.00	0.00
THR 309	0.00	0.00	0.00	0.00	0.00	0.00
THR 310	0.00	0.00	0.00	0.00	0.00	0.00
ILE 311	0.00	0.00	0.00	0.00	0.00	0.00
LYS 312	0.00	0.00	0.00	0.00	0.00	0.00
ZN1 313	0.00	0.00	-0.02	0.02	0.00	0.00

Table S9 The calculated binding free energies using MM/PBSA method for M^{pro} DPDS complex along the whole 200 ns trajectory.

Energy (Kcal/mol)	PBSA	
	Mean	Std
$\Delta Evdw$	-29.07	3.50
$\Delta Eele$	-0.16	0.74
$\Delta Gpol\ [EPB]$	7.86	1.95
$\Delta Genpolar$	-20.27	1.69
$\Delta Gedisper$	33.39	1.95
$\Delta Gbind$	-8.24	4.46

Table S10 The calculated binding free energies using MM/PBSA method for M^{pro} DPDS complex for the last 50 ns.

Energy (Kcal/mol)	PBSA	
	Mean	Std
$\Delta Evdw$	-26.37	2.33
$\Delta Eele$	0.19	0.73
$\Delta Gpol\ [EPB]$	8.18	1.86
$\Delta Genpolar$	-19.16	1.33
$\Delta Gedisper$	32.18	1.33
$\Delta Gbind$	-5.00	3.11

Table S11: Cluster 1 at 88ns; Optimized coordinates at SMD(**water**)-D3BJ-B3LYP/6-311G(d,p),ccPVTZ. Constrained atoms (*first column = -1*): 1,2,3,4,5,6,7,8,9,10,22,23,24,25,26,27,28,29,30,31,32,33,34,35,36,37,38,39,51,52,53,54,55,56,57,58,59,60,61,62,63,64,65,66,67,68,74,75,76,77,78,79,80, 81,82,83,84,85,86,87,88,89,90,91,103,104,105,106,107,108,109,110

A) **RC**

E= -8828.101079 Ha

G= -8827.679970 Ha

C	-1	0.74069200	5.59311200	4.86475600
H	-1	1.24877200	6.36689900	5.44108600
C	-1	1.73363400	4.97548900	3.90010900
O	-1	2.84803000	4.63123400	4.24147000
H	-1	-0.13568300	6.02028400	4.37375800
H	-1	0.41218900	4.82354600	5.56744000
N	-1	1.28573300	4.85095200	2.59929000
H	-1	0.29473900	4.98144400	2.44433600
C	-1	1.98141000	4.14446900	1.64156900
H	-1	3.01987900	4.09774700	1.98802200
C	0	1.48794600	2.70030000	1.40738500
H	0	2.12001700	2.24511200	0.64334000
H	0	1.64377300	2.14099800	2.33214300
C	0	0.05017000	2.61207700	1.00724800
N	0	-0.93632400	2.14263600	1.85530700
H	0	-0.77742100	1.75209900	2.77385900
C	0	-2.12265100	2.18447400	1.19587600
H	0	-3.04570000	1.84655600	1.63696800
N	0	-1.97085800	2.68453200	-0.01761700
C	0	-0.61936800	2.94759900	-0.14620400
H	0	-0.20569400	3.35665100	-1.05257700
C	-1	2.04660700	4.94675200	0.29021700
O	-1	2.17894200	4.38101900	-0.78189100
N	-1	1.97830800	6.29336400	0.43437300
H	-1	1.87842400	6.65581100	1.36879600
C	-1	2.09585200	7.20605500	-0.68776500

H	-1	2.17696800	6.60449500	-1.59146300
H	-1	1.21540100	7.84991800	-0.76300200
H	-1	2.98766800	7.83262000	-0.59551400
C	-1	10.32413000	2.74446200	-3.06840300
H	-1	11.23345700	3.33213400	-2.94324600
C	-1	10.13941500	1.86748100	-1.84386600
O	-1	10.32844300	2.27846200	-0.71546700
H	-1	10.37775200	2.18170200	-4.00246600
H	-1	9.48486700	3.44226100	-3.12608800
N	-1	9.75780600	0.57007100	-2.10714700
H	-1	9.43867300	0.35494500	-3.04073700
C	-1	9.27625400	-0.32715500	-1.02284400
H	-1	9.55902400	0.17737700	-0.09534800
C	0	7.75767900	-0.52261500	-1.06148300
H	0	7.47479100	-1.03120000	-1.98801100
H	0	7.48032400	-1.17582800	-0.23233300
C	0	7.02038000	0.81280500	-0.95794200
H	0	7.34451300	1.37123200	-0.07618600
H	0	7.22903900	1.43276800	-1.83163500
S	0	5.19845000	0.67096900	-0.89913200
C	0	4.95817900	0.20601000	0.84317500
H	0	3.88700400	0.07498200	0.99494700
H	0	5.46427200	-0.73096200	1.07206500
H	0	5.32083100	0.99976400	1.49854200
C	-1	10.05580300	-1.67162500	-1.05967600
O	-1	9.59866600	-2.67282200	-0.53177900
N	-1	11.26206000	-1.62488700	-1.67657800
H	-1	11.54920200	-0.73818900	-2.05867500
C	-1	12.16300600	-2.76184100	-1.71336800
H	-1	11.63925300	-3.61028900	-1.27637800
H	-1	12.44476400	-3.00362300	-2.74167600
H	-1	13.07019900	-2.56730100	-1.13372000
C	-1	-8.71312800	1.08478300	-4.11285600
H	-1	-9.63217100	1.51216600	-4.51715500
C	-1	-8.43442100	1.71621900	-2.76581300
O	-1	-9.25943900	1.68439000	-1.85610300

H	-1	-7.90390200	1.21663000	-4.83267900
H	-1	-8.88571600	0.01742900	-3.96110500
N	-1	-7.22331200	2.30754000	-2.62258300
H	-1	-6.57265300	2.29909200	-3.39268500
C	-1	-6.73453500	2.80624800	-1.34023600
H	-1	-7.07558600	2.09192200	-0.58301800
C	0	-5.21038800	2.81784200	-1.35348000
H	0	-4.84610600	3.54052000	-2.08378700
H	0	-4.84256600	3.10786500	-0.37297800
S	0	-4.51848500	1.15616700	-1.74494700
H	0	-3.31137000	1.44581400	-1.20436500
C	-1	-7.24118400	4.20059700	-0.90059700
O	-1	-6.47877500	5.13631700	-0.71619000
N	-1	-8.58150700	4.24797600	-0.71775600
H	-1	-9.10679800	3.39968900	-0.91295600
C	-1	-9.27095400	5.45977200	-0.31646000
H	-1	-9.78751500	5.31968200	0.63752900
H	-1	-8.52801800	6.24775700	-0.20549400
H	-1	-10.00406200	5.76119000	-1.07028500
C	-1	-5.55909200	0.13234100	1.68140000
H	-1	-5.42083000	0.84756800	0.86848900
C	-1	-4.70341000	-1.07569400	1.36802700
O	-1	-3.47945900	-1.00213800	1.29649300
H	-1	-6.62081500	-0.09944700	1.77665100
H	-1	-5.20437300	0.58991300	2.60649400
N	-1	-5.37150100	-2.23119300	1.15863600
H	-1	-6.37370200	-2.26708300	1.28529800
C	-1	-4.73541900	-3.46963400	0.84146700
H	-1	-4.00238100	-3.27305700	0.05372800
C	0	-3.91798200	-4.03704800	2.05181300
H	0	-3.79901200	-3.23372000	2.78004200
H	0	-4.47124500	-4.84126300	2.54405600
C	0	-2.54359500	-4.50662000	1.59458200
H	0	-2.62181000	-5.34152600	0.89538200
H	0	-2.03320800	-3.68659800	1.08898800
S	0	-1.49344800	-5.02404400	2.99698700

C	0	0.04307000	-5.33511500	2.06968200
H	0	-0.10624700	-6.12729000	1.33378200
H	0	0.37562400	-4.42483300	1.57223600
H	0	0.80199900	-5.64958400	2.78693600
C	-1	-5.72382200	-4.56702200	0.35857100
O	-1	-6.86535400	-4.62072600	0.78908400
N	-1	-5.18451800	-5.44753800	-0.51801700
H	-1	-4.27514900	-5.23627300	-0.90043000
C	-1	-5.92699000	-6.58062300	-1.04161700
H	-1	-6.88316700	-6.61626100	-0.52293000
H	-1	-6.11080500	-6.47084500	-2.11432600
H	-1	-5.38644700	-7.51417700	-0.86661900
Se	0	-0.40211200	-0.64384000	-0.74235100
Se	0	0.24485600	-1.29258600	1.40713900
C	0	-1.23775500	-2.26220100	-1.39855600
C	0	-2.44051300	-2.13328800	-2.09550000
C	0	-3.05041100	-3.26229900	-2.63982500
C	0	-2.47007600	-4.51936300	-2.47679500
C	0	-1.27699200	-4.64463300	-1.76463300
C	0	-0.65408000	-3.51935900	-1.23238600
C	0	1.97381800	-2.12969000	1.14046000
C	0	2.61472400	-2.58022000	2.29852900
C	0	3.84880100	-3.21785100	2.20033900
C	0	4.44626100	-3.41009800	0.95436700
C	0	3.80144300	-2.95555700	-0.19349100
C	0	2.56646000	-2.31480400	-0.10562400
H	0	-2.90099900	-1.16017900	-2.21093400
H	0	-3.98651100	-3.15852200	-3.17574000
H	0	-2.94728300	-5.39717600	-2.89713200
H	0	-0.82620200	-5.62002900	-1.62421200
H	0	0.27045000	-3.62401900	-0.68172900
H	0	2.15250700	-2.43883100	3.26926400
H	0	4.34243600	-3.56428300	3.10099600
H	0	5.40723400	-3.90487500	0.88053900
H	0	4.26070900	-3.09132800	-1.16556100
H	0	2.07339300	-1.95823600	-1.00110600

B) **TS1 img= -1209.2836 cm^{-1}**

E= -8828.089782 Ha

G= -8827.668208 Ha

C	-1	0.98897400	5.51820400	5.09336000
H	-1	1.51000300	6.25360200	5.70717300
C	-1	1.96621700	4.94808400	4.08445300
O	-1	3.08092000	4.57552900	4.39358100
H	-1	0.11147500	5.98004200	4.63697200
H	-1	0.66079300	4.71270700	5.75470700
N	-1	1.50374500	4.90159400	2.78358800
H	-1	0.51243700	5.04908900	2.64707700
C	-1	2.18306700	4.24488400	1.77991800
H	-1	3.22459400	4.16973400	2.11164000
C	0	1.66533700	2.82306300	1.45445700
H	0	2.26956600	2.42360100	0.64020300
H	0	1.84865800	2.19774800	2.33106400
C	0	0.21520800	2.70688100	1.08609200
N	0	-0.74337600	2.32024900	2.01151400
H	0	-0.56051000	2.10849800	2.98399400
C	0	-1.92501900	2.16048500	1.39154400
H	0	-2.82908200	1.83987900	1.87848400
N	0	-1.79313500	2.45627500	0.10621800
C	0	-0.46611500	2.79751600	-0.10311600
H	0	-0.09270900	3.05281300	-1.07980800
C	-1	2.24146300	5.12219800	0.47557200
O	-1	2.35760000	4.61729800	-0.62831900
N	-1	2.18694200	6.45895300	0.69674600
H	-1	2.10003400	6.76846500	1.65128300
C	-1	2.30113400	7.43300000	-0.37293000
H	-1	2.36741800	6.88318200	-1.31017700
H	-1	1.42583500	8.08754300	-0.40189600
H	-1	3.19951100	8.04571600	-0.25491700
C	-1	10.46345300	3.04453500	-3.09244000
H	-1	11.37932700	3.61640100	-2.94397500
C	-1	10.28346300	2.10088500	-1.91777900

O	-1	10.48790800	2.44535700	-0.76996800
H	-1	10.50228400	2.53541300	-4.05749700
H	-1	9.63001000	3.75157600	-3.10126500
N	-1	9.88745500	0.82380900	-2.25019200
H	-1	9.55665300	0.66491900	-3.19097400
C	-1	9.40889300	-0.12953800	-1.21342800
H	-1	9.70592700	0.31899800	-0.26193100
C	0	7.89100400	-0.32020500	-1.25957400
H	0	7.59921100	-0.66346300	-2.25694300
H	0	7.62897800	-1.10879100	-0.55283400
C	0	7.14959700	0.96846200	-0.91231800
H	0	7.42105200	1.31258800	0.08896600
H	0	7.40691800	1.76421400	-1.61454700
S	0	5.32781400	0.85126000	-1.00709200
C	0	4.96671600	-0.23985900	0.40534900
H	0	3.88157200	-0.26536100	0.50954600
H	0	5.33285200	-1.25239000	0.23564800
H	0	5.40259200	0.16554400	1.32024900
C	-1	10.17585800	-1.47628000	-1.33510900
O	-1	9.71514000	-2.50199000	-0.85999500
N	-1	11.37603900	-1.40473800	-1.96138700
H	-1	11.66724200	-0.50019900	-2.29559000
C	-1	12.26618700	-2.54535400	-2.07251000
H	-1	11.73930700	-3.41282600	-1.67877400
H	-1	12.53507100	-2.73059900	-3.11589500
H	-1	13.18106600	-2.39181600	-1.49263000
C	-1	-8.59789000	1.60843600	-4.02272500
H	-1	-9.51715200	2.06591200	-4.39208400
C	-1	-8.29957900	2.15993100	-2.64510600
O	-1	-9.11532600	2.08324200	-1.72975000
H	-1	-7.79500000	1.77417200	-4.74263900
H	-1	-8.77858200	0.53567700	-3.93000300
N	-1	-7.08152100	2.73124300	-2.48155400
H	-1	-6.43918100	2.76177100	-3.25804200
C	-1	-6.57518200	3.15200500	-1.17858500
H	-1	-6.91484400	2.39958600	-0.45890100

C	0	-5.03741900	3.22390100	-1.19943700
H	0	-4.75329200	4.01075100	-1.90153000
H	0	-4.72068900	3.55673000	-0.21233000
S	0	-4.10161100	1.69416000	-1.60103500
H	0	-2.77037400	2.15056500	-0.71716400
C	-1	-7.06454300	4.52396100	-0.65526600
O	-1	-6.29175100	5.44094500	-0.42564100
N	-1	-8.40237900	4.57202700	-0.45495300
H	-1	-8.93735200	3.74072700	-0.69237600
C	-1	-9.07660500	5.76484000	0.02195800
H	-1	-9.58450700	5.57506600	0.97199800
H	-1	-8.32543400	6.53890500	0.16944400
H	-1	-9.81471500	6.11487600	-0.70551800
C	-1	-5.39277400	0.30104700	1.67352300
H	-1	-5.25645600	1.06019900	0.90104900
C	-1	-4.55140700	-0.89432000	1.28256300
O	-1	-3.32761700	-0.82736300	1.20235200
H	-1	-6.45552000	0.07323900	1.76691100
H	-1	-5.02435500	0.70222000	2.61915200
N	-1	-5.23211000	-2.03036900	1.01512700
H	-1	-6.23320400	-2.06493600	1.15053600
C	-1	-4.61057600	-3.25412400	0.62137200
H	-1	-3.88398600	-3.01928200	-0.16187900
C	0	-3.80494500	-3.89611400	1.79780000
H	0	-3.58686700	-3.10736800	2.51878800
H	0	-4.42146900	-4.64105900	2.30888400
C	0	-2.49057500	-4.49829600	1.31829200
H	0	-2.65316800	-5.29413700	0.58952500
H	0	-1.88399200	-3.72164400	0.85073600
S	0	-1.54167300	-5.18268900	2.72311200
C	0	-0.00049000	-5.59935500	1.85146800
H	0	-0.19121800	-6.32537200	1.05917600
H	0	0.45476000	-4.70325700	1.43346400
H	0	0.67877300	-6.03710900	2.58329300
C	-1	-5.61389600	-4.31379300	0.08759000
O	-1	-6.75130300	-4.38231600	0.52680100

N	-1	-5.09185000	-5.14693000	-0.84341200
H	-1	-4.18433000	-4.92301000	-1.22332500
C	-1	-5.84975100	-6.24277700	-1.42233700
H	-1	-6.80084900	-6.29967700	-0.89625700
H	-1	-6.04372500	-6.07047700	-2.48511300
H	-1	-5.31598600	-7.18923200	-1.30664300
Se	0	0.41846500	-0.67838500	-0.20737000
Se	0	0.06548800	-1.24080800	2.04043500
C	0	-0.49682000	-2.13546100	-1.08942800
C	0	-1.74118800	-1.89337900	-1.67359000
C	0	-2.40230500	-2.92526000	-2.33893800
C	0	-1.83003500	-4.19447000	-2.40737900
C	0	-0.58933900	-4.43327500	-1.81597700
C	0	0.08489100	-3.40381400	-1.16502200
C	0	1.54027400	-2.42683300	2.46323100
C	0	1.51482100	-2.99831400	3.73844200
C	0	2.52223800	-3.88084800	4.11971600
C	0	3.55484900	-4.19665700	3.23668100
C	0	3.57630500	-3.61692000	1.96952800
C	0	2.57174300	-2.73246600	1.57937700
H	0	-2.19432100	-0.91310400	-1.59898500
H	0	-3.36947500	-2.73869900	-2.79082700
H	0	-2.35156100	-4.99676400	-2.91667000
H	0	-0.14479900	-5.42034700	-1.86267100
H	0	1.04857200	-3.58648100	-0.70895500
H	0	0.70838700	-2.76741100	4.42483200
H	0	2.49466500	-4.32577200	5.10776500
H	0	4.33487400	-4.88702900	3.53464800
H	0	4.37558000	-3.85163100	1.27580400
H	0	2.58916800	-2.29113500	0.59168600

C) **post-TCI**

E= -8828.102212 Ha

G= -8827.675945 Ha

C	-1	2.11889300	5.47859000	5.10946700
H	-1	2.69849300	6.21437000	5.66780100
C	-1	3.03443500	4.79257100	4.11494000

O	-1	4.13205300	4.37033500	4.42133600
H	-1	1.25968200	5.96677400	4.64576300
H	-1	1.75941000	4.73397700	5.82377000
N	-1	2.53759100	4.70034600	2.82928500
H	-1	1.55389300	4.90065600	2.70567000
C	-1	3.15057500	3.94687800	1.85132200
H	-1	4.19386200	3.82659800	2.16452000
C	0	2.58687100	2.53049700	1.63753100
H	0	3.24806600	2.00425900	0.94513000
H	0	2.63970300	2.00536300	2.59258200
C	0	1.18678200	2.44597600	1.12022100
N	0	0.23783700	1.62921300	1.71990300
H	0	0.41013900	1.04099100	2.52647000
C	0	-0.88219700	1.59667200	0.99849800
H	0	-1.74968900	0.98919600	1.21405800
N	0	-0.69824900	2.41764900	-0.03425800
C	0	0.57571300	2.95275900	0.00770600
H	0	0.93793800	3.61443800	-0.75568400
C	-1	3.22949500	4.74278000	0.49697100
O	-1	3.28709100	4.16942000	-0.57768600
N	-1	3.26100000	6.09083000	0.64056100
H	-1	3.21681900	6.45917300	1.57697300
C	-1	3.40689900	6.99323800	-0.48655200
H	-1	3.41645100	6.38769900	-1.39118200
H	-1	2.57219000	7.69774500	-0.53500500
H	-1	4.34324300	7.55517200	-0.42397000
C	-1	11.21927000	1.96185200	-3.11900900
H	-1	12.17137400	2.48374400	-3.02401900
C	-1	11.01215600	1.09979700	-1.88748500
O	-1	11.26566500	1.49606600	-0.76648400
H	-1	11.20318500	1.39696600	-4.05319000
H	-1	10.42999600	2.71724300	-3.15133800
N	-1	10.53184300	-0.16749400	-2.13595700
H	-1	10.16845900	-0.35905300	-3.05843400
C	-1	10.02245200	-1.02804500	-1.03549200
H	-1	10.37002200	-0.54600300	-0.11784900

C	0	8.49623700	-1.12343500	-1.04046400
H	0	8.16206200	-1.59492400	-1.96960400
H	0	8.19864100	-1.77524900	-0.21784100
C	0	7.84825800	0.25231500	-0.89592600
H	0	8.21771000	0.76666000	-0.00510800
H	0	8.08265200	0.87978600	-1.75776100
S	0	6.02307400	0.21867300	-0.81810600
C	0	5.78471300	-0.42006600	0.86819400
H	0	4.71046700	-0.49106400	1.03322300
H	0	6.22412700	-1.41166200	0.98068500
H	0	6.21707400	0.26429000	1.60013500
C	-1	10.70382300	-2.42476100	-1.09377400
O	-1	10.19408700	-3.39130600	-0.54988400
N	-1	11.89005500	-2.46323900	-1.74905400
H	-1	12.22677800	-1.59895800	-2.14188800
C	-1	12.70676000	-3.66102900	-1.81228500
H	-1	12.13856900	-4.47045600	-1.35716900
H	-1	12.93779700	-3.92184000	-2.84855900
H	-1	13.64345200	-3.53126100	-1.26218800
C	-1	-7.91127500	1.65223800	-3.55379000
H	-1	-8.81024700	2.14362500	-3.92948300
C	-1	-7.54585800	2.26206600	-2.21756100
O	-1	-8.34161800	2.28834100	-1.28198700
H	-1	-7.11812000	1.72675400	-4.29926100
H	-1	-8.15392100	0.59971000	-3.39451300
N	-1	-6.29206900	2.76631800	-2.11412700
H	-1	-5.66847300	2.71198300	-2.90450400
C	-1	-5.72828300	3.22845500	-0.84866500
H	-1	-6.09521700	2.54022500	-0.07985800
C	0	-4.17437800	3.12446100	-0.86804600
H	0	-3.76253200	4.13225200	-0.88919800
H	0	-3.82031800	2.65027900	0.04515300
S	0	-3.36700900	2.31025400	-2.30290900
H	0	-1.38431100	2.57225600	-0.77332500
C	-1	-6.12122800	4.65540000	-0.39642500
O	-1	-5.28906400	5.53474900	-0.23789800

N	-1	-7.44829800	4.79728400	-0.17087700
H	-1	-8.03816900	3.98830100	-0.34757200
C	-1	-8.03725200	6.05467100	0.24976600
H	-1	-8.53173900	5.95118100	1.21998700
H	-1	-7.23733700	6.78813800	0.33547100
H	-1	-8.77089300	6.40735900	-0.48094100
C	-1	-4.64935100	0.47763300	2.13918100
H	-1	-4.48671100	1.18151100	1.32064800
C	-1	-3.89152300	-0.78760900	1.80060900
O	-1	-2.66826900	-0.80099200	1.69078200
H	-1	-5.72114500	0.32146800	2.26849000
H	-1	-4.23378700	0.90874200	3.05145500
N	-1	-4.64608600	-1.89308700	1.61494400
H	-1	-5.64358600	-1.85810600	1.77389500
C	-1	-4.10906500	-3.17331100	1.28040200
H	-1	-3.38978300	-3.02879200	0.46936800
C	0	-3.34905100	-3.77248200	2.49679700
H	0	-2.66440000	-2.99769700	2.84473800
H	0	-4.06605100	-3.96523000	3.29981500
C	0	-2.55760300	-5.03499800	2.16894500
H	0	-3.20456400	-5.90960300	2.09699900
H	0	-2.02951800	-4.92325100	1.21851200
S	0	-1.31435500	-5.34536400	3.47474100
C	0	-0.53100200	-6.82862600	2.77167000
H	0	-1.25559200	-7.63867700	2.67241300
H	0	-0.09233000	-6.60310500	1.79801100
H	0	0.25905400	-7.13778400	3.45705700
C	-1	-5.18782600	-4.19789500	0.83150800
O	-1	-6.31589400	-4.17095900	1.29814800
N	-1	-4.74019400	-5.11417100	-0.06011000
H	-1	-3.83085600	-4.96757500	-0.47174100
C	-1	-5.57721100	-6.19176600	-0.55761500
H	-1	-6.51648500	-6.15987800	-0.00868300
H	-1	-5.78691500	-6.06898200	-1.62414000
H	-1	-5.09867100	-7.16124700	-0.39805300
Se	0	-2.91277600	0.11158900	-1.83705400

Se	0	-1.56176100	-2.83468700	-1.60490800
C	0	-4.64469500	-0.73983700	-2.01918700
C	0	-5.69234100	-0.40594100	-1.16099400
C	0	-6.88994900	-1.11407400	-1.21371300
C	0	-7.03821800	-2.17198300	-2.10896700
C	0	-5.99408800	-2.49891400	-2.97393300
C	0	-4.80162400	-1.77793700	-2.94088200
C	0	-0.16711100	-2.09070700	-0.50541700
C	0	0.03570700	-2.53888400	0.80913200
C	0	1.05060000	-2.00486200	1.59943700
C	0	1.89226200	-1.01424800	1.09602500
C	0	1.70017200	-0.55579100	-0.20703800
C	0	0.67861800	-1.08077100	-0.99621300
H	0	-5.57320100	0.38801000	-0.44060300
H	0	-7.69810600	-0.84450500	-0.54436600
H	0	-7.96210900	-2.73729900	-2.13455200
H	0	-6.10568900	-3.31543200	-3.67770900
H	0	-3.99245300	-2.03387200	-3.61250300
H	0	-0.60297300	-3.30965600	1.21873700
H	0	1.18200100	-2.36831400	2.61298200
H	0	2.67248300	-0.59235800	1.71701700
H	0	2.34060800	0.22185100	-0.60834900
H	0	0.53971500	-0.71278900	-2.00607700

D) **TS2 img= -967.796 cm^{-1}**

E= -8828.077273 Ha

G= -8827.658490 Ha

C	-1	1.02980000	5.68840000	4.89300000
H	-1	1.55350000	6.45020000	5.47130000
C	-1	2.00820000	5.05860000	3.92140000
O	-1	3.11770000	4.69320000	4.25670000
H	-1	0.15920000	6.13340000	4.40750000
H	-1	0.69060000	4.92110000	5.59290000
N	-1	1.55340000	4.94870000	2.62160000
H	-1	0.70710000	5.36990000	2.38120000

C	-1	2.23300000	4.23540000	1.65760000
H	-1	3.27190000	4.16850000	1.99990000
C	0	1.87483900	2.74326200	1.47712700
H	0	2.71477100	2.27755500	0.95881600
H	0	1.86131600	2.31625000	2.48535700
C	0	0.65165300	2.31431100	0.74940900
N	0	-0.65612300	2.51510200	1.16330300
H	0	-0.94767400	3.08506100	1.94663200
C	0	-1.48029600	1.80555700	0.35941500
H	0	-2.54880900	1.77066500	0.48115300
N	0	-0.77155500	1.17108200	-0.56506200
C	0	0.55551100	1.47634200	-0.32707700
H	0	1.34980100	1.04676000	-0.91493800
C	-1	2.30740000	5.04330000	0.31020000
O	-1	2.42570000	4.48100000	-0.76530000
N	-1	2.26300000	6.39010000	0.46160000
H	-1	2.17300000	6.74940000	1.39820000
C	-1	2.39230000	7.30650000	-0.65630000
H	-1	2.45960000	6.70830000	-1.56340000
H	-1	1.52300000	7.96600000	-0.72490000
H	-1	3.29530000	7.91690000	-0.56420000
C	-1	10.53260000	2.71460000	-3.09120000
H	-1	11.45250000	3.28560000	-2.96640000
C	-1	10.33710000	1.83460000	-1.87050000
O	-1	10.53750000	2.23630000	-0.74070000
H	-1	10.57290000	2.15580000	-4.02830000
H	-1	9.70540000	3.42720000	-3.14210000
N	-1	9.93200000	0.54540000	-2.13890000
H	-1	9.60560000	0.34080000	-3.07240000
C	-1	9.43870000	-0.34880000	-1.05800000
H	-1	9.73390000	0.14550000	-0.12840000
C	0	7.91728500	-0.52169700	-1.11519000
H	0	7.64165700	-0.95463100	-2.08177400
H	0	7.62383900	-1.23347400	-0.34226000
C	0	7.19284700	0.80944200	-0.91800100
H	0	7.41746700	1.23406700	0.06367700

H	0	7.51329400	1.53363100	-1.66964700
S	0	5.37672200	0.73738400	-1.11506700
C	0	4.87622800	-0.10946200	0.41655800
H	0	3.78686300	-0.15471500	0.40795300
H	0	5.27164200	-1.12433800	0.45760100
H	0	5.20481000	0.45517600	1.29084000
C	-1	10.19470000	-1.70660000	-1.10390000
O	-1	9.72200000	-2.70240000	-0.57980000
N	-1	11.39920000	-1.67770000	-1.72540000
H	-1	11.70040000	-0.79410000	-2.10410000
C	-1	12.28010000	-2.83000000	-1.77150000
H	-1	11.74320000	-3.67140000	-1.33690000
H	-1	12.55370000	-3.07130000	-2.80220000
H	-1	13.19260000	-2.65430000	-1.19440000
C	-1	-8.53450000	1.39260000	-4.07180000
H	-1	-9.44740000	1.83810000	-4.47040000
C	-1	-8.23980000	2.01210000	-2.72260000
O	-1	-9.06190000	1.98990000	-1.80990000
H	-1	-7.72580000	1.51410000	-4.79400000
H	-1	-8.72510000	0.32770000	-3.92490000
N	-1	-7.01790000	2.58110000	-2.58090000
H	-1	-6.37050000	2.56570000	-3.35350000
C	-1	-6.51590000	3.06490000	-1.29660000
H	-1	-6.86670000	2.35250000	-0.54320000
C	0	-4.96280300	3.07094000	-1.32737800
H	0	-4.62163500	4.08789400	-1.52290100
H	0	-4.55801300	2.77345000	-0.36226000
S	0	-4.13503800	2.11171500	-2.65058600
H	0	-0.95944200	-0.16277800	-1.04614900
C	-1	-6.99660000	4.46580000	-0.85030000
O	-1	-6.21710000	5.38660000	-0.66240000
N	-1	-8.33510000	4.53530000	-0.66100000
H	-1	-8.87580000	3.69730000	-0.85850000
C	-1	-9.00180000	5.75680000	-0.25080000
H	-1	-9.51720000	5.62070000	0.70440000
H	-1	-8.24480000	6.53110000	-0.13860000

H	-1	-9.73230000	6.07490000	-1.00030000
C	-1	-5.37620000	0.35550000	1.70630000
H	-1	-5.22800000	1.07140000	0.89500000
C	-1	-4.54300000	-0.86580000	1.38120000
O	-1	-3.31790000	-0.81370000	1.30760000
H	-1	-6.44130000	0.14130000	1.80350000
H	-1	-5.01000000	0.80150000	2.63150000
N	-1	-5.23170000	-2.00860000	1.16970000
H	-1	-6.23390000	-2.02770000	1.30020000
C	-1	-4.61830000	-3.25700000	0.84400000
H	-1	-3.88530000	-3.06780000	0.05430000
C	0	-3.81447100	-3.84070500	2.05200200
H	0	-3.62706300	-3.02406300	2.75001700
H	0	-4.41542700	-4.58643000	2.57966100
C	0	-2.47927000	-4.42193900	1.60416900
H	0	-2.61611900	-5.27206900	0.93239000
H	0	-1.90546800	-3.65608600	1.07717300
S	0	-1.49275600	-4.96573400	3.04425800
C	0	0.05534000	-5.36927900	2.17728800
H	0	-0.09073800	-6.20944700	1.49589100
H	0	0.41412600	-4.50222500	1.62415500
H	0	0.79286300	-5.64441900	2.93199900
C	-1	-5.62780000	-4.33370000	0.35910000
O	-1	-6.76850000	-4.36970000	0.79350000
N	-1	-5.10710000	-5.21900000	-0.52410000
H	-1	-4.19570000	-5.02160000	-0.90890000
C	-1	-5.87120000	-6.33610000	-1.05080000
H	-1	-6.82600000	-6.35780000	-0.52860000
H	-1	-6.05710000	-6.21760000	-2.12220000
H	-1	-5.34640000	-7.27990000	-0.88260000
Se	0	-3.85036100	0.04755900	-1.89266500
Se	0	-0.77416900	-1.78584600	-1.38076500
C	0	-5.49913100	-0.92474900	-2.16467400
C	0	-6.67028900	-0.58526300	-1.48556100
C	0	-7.78387000	-1.41444100	-1.56582400
C	0	-7.72136000	-2.60397700	-2.29283000

C	0	-6.54872700	-2.94806200	-2.96161600
C	0	-5.43900200	-2.10397300	-2.91093700
C	0	0.49339000	-2.02479500	0.05690900
C	0	0.22335800	-1.50602100	1.32953300
C	0	1.14853400	-1.66100800	2.36036200
C	0	2.34381000	-2.34485500	2.14122300
C	0	2.60914000	-2.87306100	0.87871900
C	0	1.69375300	-2.71010600	-0.15984500
H	0	-6.70693800	0.30261200	-0.87364400
H	0	-8.69202800	-1.13917800	-1.04290400
H	0	-8.58180500	-3.26099400	-2.33331400
H	0	-6.49444300	-3.87149900	-3.52600900
H	0	-4.52871800	-2.36885800	-3.43473500
H	0	-0.70842700	-0.98726300	1.51500000
H	0	0.92842200	-1.25037200	3.33940800
H	0	3.06014200	-2.46555500	2.94518000
H	0	3.53582500	-3.40489100	0.69558000
H	0	1.91611700	-3.10996400	-1.1420260

D) **PC**
E= -8828.088615 Ha
G= -8827.665580 Ha

C	-1	2.00304700	5.40221500	4.98874000
H	-1	2.58057500	6.14799300	5.53584100
C	-1	2.92059000	4.70356100	4.00490500
O	-1	4.01930300	4.28902700	4.31783600
H	-1	1.14259700	5.88093500	4.51753800
H	-1	1.64546900	4.66761900	5.71428600
N	-1	2.42423300	4.59040800	2.72075200
H	-1	1.43998100	4.78610800	2.59398900
C	-1	3.03939000	3.82379900	1.75439000
H	-1	4.08290300	3.71105100	2.06967100
C	0	2.44173500	2.42036300	1.56070000
H	0	2.98131700	1.91827100	0.75386800
H	0	2.62621300	1.84563400	2.47222700
C	0	0.97340400	2.45638200	1.28621000

N	0	0.14937000	1.37190200	1.50623900
H	0	0.45068200	0.45332600	1.80863000
C	0	-1.12866800	1.74175200	1.21690000
H	0	-1.95176600	1.05555800	1.32007200
N	0	-1.18250900	3.00202900	0.82949900
C	0	0.12567700	3.45285000	0.86129400
H	0	0.36847000	4.46481400	0.57926300
C	-1	3.11637200	4.59908600	0.38814500
O	-1	3.17563200	4.00953700	-0.67763400
N	-1	3.14416600	5.94924300	0.51113600
H	-1	3.09886900	6.33171600	1.44181100
C	-1	3.28782300	6.83473000	-0.62960600
H	-1	3.29915100	6.21547800	-1.52488400
H	-1	2.45122300	7.53615600	-0.68890200
H	-1	4.22263800	7.40008400	-0.57550300
C	-1	11.11416200	1.78487900	-3.18406300
H	-1	12.06483900	2.31073300	-3.09694400
C	-1	10.90919800	0.94116600	-1.93954300
O	-1	11.16145500	1.35518800	-0.82469500
H	-1	11.09974700	1.20575700	-4.10951300
H	-1	10.32285700	2.53755400	-3.22800900
N	-1	10.43225600	-0.33086500	-2.16858400
H	-1	10.06963600	-0.53767900	-3.08818600
C	-1	9.92520900	-1.17646900	-1.05547500
H	-1	10.27120500	-0.67898500	-0.14514700
C	0	8.39612500	-1.23364000	-1.01604200
H	0	8.00760700	-1.75942300	-1.89283800
H	0	8.10663100	-1.80515600	-0.13241300
C	0	7.82371600	0.18529100	-0.95279600
H	0	8.36776400	0.78641300	-0.22042800
H	0	7.92623800	0.68147700	-1.91907900
S	0	6.05080900	0.30868900	-0.53806800
C	0	6.12307600	-0.01937400	1.25257600
H	0	5.14684300	0.22813000	1.66977200
H	0	6.34927200	-1.06573000	1.45990200
H	0	6.87595600	0.62218100	1.71357700

C	-1	10.61032300	-2.57165500	-1.09188100
O	-1	10.10300900	-3.53120400	-0.53369600
N	-1	11.79665200	-2.61696700	-1.74669500
H	-1	12.13113400	-1.75786300	-2.15258000
C	-1	12.61663400	-3.81335600	-1.79148200
H	-1	12.05056000	-4.61727300	-1.32412600
H	-1	12.84853400	-4.08933100	-2.82362700
H	-1	13.55288600	-3.67267400	-1.24332300
C	-1	-8.01540800	1.41695300	-3.61622900
H	-1	-8.91564500	1.90011600	-3.99948000
C	-1	-7.65185700	2.04812200	-2.28942900
O	-1	-8.44782000	2.08649700	-1.35444900
H	-1	-7.22234300	1.48222400	-4.36266200
H	-1	-8.25523500	0.36632900	-3.44092800
N	-1	-6.39923900	2.55688700	-2.19350600
H	-1	-5.77560600	2.49266700	-2.98297400
C	-1	-5.83740100	3.04062000	-0.93421800
H	-1	-6.20217200	2.36257300	-0.15620500
C	0	-4.28291400	2.98699400	-0.99695300
H	0	-3.91326300	4.00394800	-1.11153400
H	0	-3.86717900	2.60110700	-0.07048100
S	0	-3.49856500	2.09621500	-2.39572900
H	0	-1.87050400	-2.35268900	-0.21963800
C	-1	-6.23408100	4.47303600	-0.50579100
O	-1	-5.40416900	5.35661400	-0.35935500
N	-1	-7.56144900	4.61451600	-0.28166400
H	-1	-8.14910600	3.80133900	-0.44606100
C	-1	-8.15386400	5.87658200	0.11966100
H	-1	-8.64821900	5.78657900	1.09129300
H	-1	-7.35594600	6.61343300	0.19425000
H	-1	-8.88834200	6.21609000	-0.61642800
C	-1	-4.75118600	0.33866200	2.09506400
H	-1	-4.59011900	1.02933900	1.26456700
C	-1	-3.99012600	-0.92977900	1.77403400
O	-1	-2.76652300	-0.94187900	1.66623800
H	-1	-5.82258900	0.18114000	2.22594400

H	-1	-4.33693600	0.78432000	3.00001000
N	-1	-4.74126300	-2.04020300	1.60668600
H	-1	-5.73906800	-2.00550600	1.76468100
C	-1	-4.20104600	-3.32460000	1.29169900
H	-1	-3.48204700	-3.18930300	0.47840900
C	0	-3.41980200	-3.90224800	2.50949700
H	0	-2.92588100	-3.05977000	2.99400700
H	0	-4.13082600	-4.32159600	3.22714300
C	0	-2.35991100	-4.92391600	2.11414200
H	0	-2.79978400	-5.86715200	1.78923400
H	0	-1.75169400	-4.53010900	1.29748300
S	0	-1.22972800	-5.24864400	3.51933900
C	0	0.17047200	-5.95511700	2.59766700
H	0	-0.12963900	-6.86040500	2.06747400
H	0	0.56530800	-5.22285500	1.89093400
H	0	0.94486000	-6.20714900	3.32331700
C	-1	-5.27679600	-4.35820800	0.85814700
O	-1	-6.40507400	-4.32711800	1.32417100
N	-1	-4.82659100	-5.28675800	-0.01926800
H	-1	-3.91760000	-5.14398400	-0.43301600
C	-1	-5.66062900	-6.37405800	-0.50039000
H	-1	-6.60007100	-6.33633500	0.04788500
H	-1	-5.87049800	-6.26813900	-1.56868800
H	-1	-5.17949700	-7.33969500	-0.32599200
Se	0	-2.85881200	0.12291000	-1.62788900
Se	0	-0.73848200	-2.86767200	-0.99876400
C	0	-4.43121900	-0.99456100	-1.78656400
C	0	-5.65676900	-0.60238100	-1.24773200
C	0	-6.75025800	-1.46039600	-1.31522300
C	0	-6.61494200	-2.72500300	-1.88826700
C	0	-5.38512200	-3.12084200	-2.41058000
C	0	-4.29281200	-2.25485900	-2.37268900
C	0	0.59032300	-2.25178200	0.25556900
C	0	0.35371600	-2.25408400	1.63314500
C	0	1.34694500	-1.80882800	2.50476800
C	0	2.58069400	-1.38063500	2.01241300

C	0	2.81433000	-1.39676200	0.63894600
C	0	1.82211800	-1.82285800	-0.24072600
H	0	-5.75695800	0.35507000	-0.76056100
H	0	-7.70020800	-1.14328300	-0.90107000
H	0	-7.46087700	-3.40091900	-1.92079400
H	0	-5.27397500	-4.10314300	-2.85370100
H	0	-3.34133600	-2.55883000	-2.79189400
H	0	-0.59393000	-2.59344600	2.02664800
H	0	1.15380500	-1.80599700	3.57134200
H	0	3.35161300	-1.03670600	2.69118900
H	0	3.76706800	-1.07311300	0.24041500
H	0	2.00466700	-1.81174100	-1.30864400

Table S12: Cluster 1 at 88ns; Optimized coordinates at SMD(**deiethylether**)-B3LYP/6-311G(d,p),ccPVTZ. Constrained atoms (*first column = -1*): 1,2,3,4,5,6,7,8,9,10,22,23,24,25,26,27,28,29,30,31,32,33,34,35,36,37,38,39,51,52,53,54,55,56,57,58,59,60,61,62,63,64,65,66,67,68,74,75,76,77,78,79,80, 81,82,83,84,85,86,87,88,89,90,91,103,104,105,106,107,108,109,110

A) **RC**

E= -8828.094382 Ha

G= -8827.672764 Ha

C	-1	1.08046300	5.81142200	4.88862200
H	-1	1.60840900	6.57903500	5.45527200
C	-1	2.05532500	5.16118300	3.92697500
O	-1	3.16269100	4.79467400	4.26804800
H	-1	0.21242900	6.25378900	4.39626200
H	-1	0.73679300	5.05685200	5.60025700
N	-1	1.59993100	5.03385600	2.62903300
H	-1	0.61167400	5.18573200	2.47610900
C	-1	2.27559400	4.30174800	1.67621200
H	-1	3.31394800	4.23449300	2.01960700

C	0	1.79509200	2.85884400	1.44679100
H	0	2.43302500	2.42710200	0.67261500
H	0	1.97458500	2.29686500	2.36711900
C	0	0.35816500	2.72223800	1.06010900
N	0	-0.49694800	1.87938200	1.74408800
H	0	-0.23327700	1.26892400	2.50430400
C	0	-1.71200200	1.90887700	1.13882300
H	0	-2.51151600	1.24200800	1.42006500
N	0	-1.69776800	2.73422400	0.10891100
C	0	-0.41517300	3.24852600	0.04967600
H	0	-0.11264700	3.93744000	-0.72131000
C	-1	2.35436200	5.08856700	0.31652200
O	-1	2.46973800	4.50905600	-0.75018500
N	-1	2.31784800	6.43778500	0.44712200
H	-1	2.22976400	6.81191700	1.37812100
C	-1	2.45238800	7.33606700	-0.68469000
H	-1	2.51627000	6.72364700	-1.58245600
H	-1	1.58680800	7.99937100	-0.76351700
H	-1	3.35879700	7.94269500	-0.60189000
C	-1	10.56648000	2.66099100	-3.04752000
H	-1	11.48961500	3.22866600	-2.93147700
C	-1	10.36595600	1.80100900	-1.81345400
O	-1	10.56856400	2.21892700	-0.68996600
H	-1	10.60366500	2.08768500	-3.97595200
H	-1	9.74338900	3.37743700	-3.10948700
N	-1	9.95354200	0.51011800	-2.06216700
H	-1	9.62604400	0.29305900	-2.99238900
C	-1	9.45518200	-0.36450700	-0.96706700
H	-1	9.75300700	0.14261800	-0.04575900
C	0	7.94007700	-0.56921100	-1.00434200
H	0	7.65371600	-1.01782800	-1.96144300
H	0	7.69718100	-1.29967400	-0.23176900
C	0	7.15602900	0.72211200	-0.78874200
H	0	7.46348300	1.22225100	0.13348500
H	0	7.32506400	1.42355100	-1.60908700
S	0	5.34245600	0.48235600	-0.73555600

C	0	5.15671100	-0.26165500	0.91522200
H	0	4.09147700	-0.44029400	1.06492100
H	0	5.68326500	-1.21288200	0.98853200
H	0	5.51608500	0.42519700	1.68452400
C	-1	10.20332200	-1.72706900	-0.99281400
O	-1	9.72505300	-2.71198900	-0.45314100
N	-1	11.40808800	-1.71457000	-1.61424400
H	-1	11.71427900	-0.83869400	-2.00637900
C	-1	12.28233300	-2.87242100	-1.64247200
H	-1	11.74067400	-3.70401900	-1.19504300
H	-1	12.55467600	-3.13109600	-2.66920400
H	-1	13.19588800	-2.69308900	-1.06793300
C	-1	-8.50776400	1.43255800	-4.01018100
H	-1	-9.41813300	1.87700400	-4.41570500
C	-1	-8.20962000	2.07100600	-2.67062600
O	-1	-9.03183900	2.06754700	-1.75783700
H	-1	-7.69832700	1.53829600	-4.73406300
H	-1	-8.70445800	0.37108700	-3.84693100
N	-1	-6.98463800	2.63555600	-2.53759000
H	-1	-6.33715000	2.60416400	-3.30975700
C	-1	-6.47975100	3.13569400	-1.26197200
H	-1	-6.83453500	2.43724700	-0.49642800
C	0	-4.95467000	3.12736100	-1.29824100
H	0	-4.59695300	3.84995500	-2.03223900
H	0	-4.55699700	3.42232900	-0.33105600
S	0	-4.29344300	1.45844300	-1.70291600
H	0	-3.06389000	1.73964100	-1.20462400
C	-1	-6.95243200	4.54588500	-0.83509100
O	-1	-6.16789200	5.46547100	-0.66274300
N	-1	-8.29061500	4.62614800	-0.64808500
H	-1	-8.83610800	3.78833200	-0.83281700
C	-1	-8.95036700	5.85761500	-0.25687900
H	-1	-9.46655900	5.73923000	0.70024600
H	-1	-8.18899200	6.62925200	-0.15650900
H	-1	-9.67903900	6.16827900	-1.01125000
C	-1	-5.35556700	0.46612900	1.78273600

H	-1	-5.20374300	1.16966600	0.96210400
C	-1	-4.52921400	-0.76455700	1.47891000
O	-1	-3.30416900	-0.72008800	1.40235100
H	-1	-6.42201500	0.25999000	1.88398600
H	-1	-4.98698700	0.92471900	2.70190800
N	-1	-5.22463200	-1.90630800	1.28357700
H	-1	-6.22692900	-1.91766800	1.41400100
C	-1	-4.61854600	-3.16231500	0.97693200
H	-1	-3.88402300	-2.99079500	0.18473200
C	0	-3.82679100	-3.70729700	2.20222900
H	0	-3.32440700	-2.84944900	2.64960400
H	0	-4.52795900	-4.10979300	2.93909900
C	0	-2.77086100	-4.73785000	1.81524700
H	0	-3.21590300	-5.69391400	1.53351000
H	0	-2.18608800	-4.36098500	0.97367800
S	0	-1.60109400	-5.01347100	3.19507200
C	0	-0.31975500	-5.93811000	2.29477800
H	0	-0.71785500	-6.87552500	1.90045200
H	0	0.08856200	-5.33487100	1.48243100
H	0	0.47978600	-6.16334300	3.00191600
C	-1	-5.63382800	-4.24134700	0.50865600
O	-1	-6.77472900	-4.26419600	0.94357200
N	-1	-5.11824700	-5.14296200	-0.36070700
H	-1	-4.20562400	-4.95676000	-0.74835600
C	-1	-5.88864400	-6.26380400	-0.87015700
H	-1	-6.84349500	-6.27199800	-0.34788300
H	-1	-6.07376900	-6.16068900	-1.94330200
H	-1	-5.36922000	-7.20781300	-0.68745400
Se	0	-0.80472900	-0.94940500	-0.96880800
Se	0	-0.40396400	-1.80799000	1.16693900
C	0	-1.73160700	-2.45116900	-1.75989300
C	0	-2.87461800	-2.18787000	-2.51829300
C	0	-3.55175400	-3.23535300	-3.14034300
C	0	-3.10021100	-4.54618900	-2.99771000
C	0	-1.96613700	-4.80689500	-2.22629200
C	0	-1.27574000	-3.76292700	-1.61473900

C	0	1.38417700	-2.55130600	1.01674800
C	0	1.93635800	-3.04553600	2.20354400
C	0	3.19818000	-3.63462700	2.18957300
C	0	3.91900400	-3.72959800	0.99965100
C	0	3.36895500	-3.22597200	-0.17683700
C	0	2.10434900	-2.63898900	-0.17352500
H	0	-3.24078800	-1.17287100	-2.61422200
H	0	-4.44072700	-3.02519400	-3.72383600
H	0	-3.62879900	-5.35995000	-3.48049800
H	0	-1.60760700	-5.82381300	-2.11155100
H	0	-0.39028300	-3.96763500	-1.02678500
H	0	1.38104700	-2.97853800	3.13280300
H	0	3.61807100	-4.01692500	3.11334800
H	0	4.90321700	-4.18311600	0.99184800
H	0	3.92710300	-3.27848500	-1.10476200
H	0	1.68587900	-2.24298600	-1.09059800

B) **TS1 img= -1128.7924 cm^{-1}**

E= -8828.083026 Ha

G= -8827.663793 Ha

C	-1	0.98897400	5.51820400	5.09336000
H	-1	1.51000300	6.25360200	5.70717300
C	-1	1.96623600	4.94811500	4.08444500
O	-1	3.08092000	4.57552900	4.39358100
H	-1	0.11147500	5.98004200	4.63697200
H	-1	0.66079300	4.71270700	5.75470700
N	-1	1.50365200	4.90162200	2.78350600
H	-1	0.51243400	5.04907900	2.64708200
C	-1	2.18335600	4.24485700	1.78023100
H	-1	3.22463400	4.16977900	2.11152500
C	0	1.71315500	2.81451000	1.43493100
H	0	2.35653400	2.46016200	0.62830600
H	0	1.90874400	2.18201100	2.30455400
C	0	0.27841400	2.62725700	1.03011400
N	0	-0.56267600	1.82132500	1.78465000
H	0	-0.28978200	1.31404800	2.61559400

C	0	-1.74861500	1.69579200	1.16511600
H	0	-2.52877300	1.00799900	1.45553500
N	0	-1.73140600	2.40592700	0.04868500
C	0	-0.47866400	2.99166700	-0.05966600
H	0	-0.20060900	3.60301700	-0.90063400
C	-1	2.24130000	5.12221400	0.47557600
O	-1	2.35763500	4.61730000	-0.62831600
N	-1	2.18695800	6.45895300	0.69674700
H	-1	2.10003400	6.76846500	1.65128300
C	-1	2.30113400	7.43300000	-0.37293000
H	-1	2.36741800	6.88318200	-1.31017700
H	-1	1.42583500	8.08754300	-0.40189600
H	-1	3.19951100	8.04571600	-0.25491700
C	-1	10.46345300	3.04453500	-3.09244000
H	-1	11.37932700	3.61640100	-2.94397500
C	-1	10.28345100	2.10088800	-1.91778100
O	-1	10.48790800	2.44535700	-0.76996800
H	-1	10.50228400	2.53541300	-4.05749700
H	-1	9.63001000	3.75157600	-3.10126500
N	-1	9.88750600	0.82375800	-2.25021600
H	-1	9.55660300	0.66495700	-3.19096300
C	-1	9.40881600	-0.12951800	-1.21348100
H	-1	9.70597700	0.31896100	-0.26192900
C	0	7.89536000	-0.34481300	-1.25909600
H	0	7.60074000	-0.66031200	-2.26576200
H	0	7.66848600	-1.17419500	-0.58848200
C	0	7.11221900	0.90104900	-0.85640000
H	0	7.38931300	1.22658700	0.14998500
H	0	7.32135400	1.73107000	-1.53566400
S	0	5.29516300	0.70281800	-0.92567100
C	0	5.02531900	-0.50590900	0.40953000
H	0	3.94595600	-0.62660200	0.51154700
H	0	5.46354200	-1.47557800	0.17320700
H	0	5.43105900	-0.13455500	1.35303200
C	-1	10.17594000	-1.47624800	-1.33495100
O	-1	9.71511500	-2.50200000	-0.86004000

N	-1	11.37602200	-1.40474500	-1.96142100
H	-1	11.66724200	-0.50019900	-2.29559000
C	-1	12.26618700	-2.54535400	-2.07251000
H	-1	11.73930700	-3.41282600	-1.67877400
H	-1	12.53507100	-2.73059900	-3.11589500
H	-1	13.18106600	-2.39181600	-1.49263000
C	-1	-8.59789000	1.60843600	-4.02272500
H	-1	-9.51715200	2.06591200	-4.39208400
C	-1	-8.29966900	2.16013200	-2.64513600
O	-1	-9.11532600	2.08324200	-1.72975000
H	-1	-7.79500000	1.77417200	-4.74263900
H	-1	-8.77858200	0.53567700	-3.93000300
N	-1	-7.08130100	2.73062800	-2.48144100
H	-1	-6.43924900	2.76199900	-3.25808900
C	-1	-6.57493300	3.15071600	-1.17901200
H	-1	-6.91499700	2.40052100	-0.45799600
C	0	-5.03700900	3.23939600	-1.20889500
H	0	-4.76977200	4.03352100	-1.90980700
H	0	-4.71711900	3.58777600	-0.22822500
S	0	-4.09148100	1.72138800	-1.61720100
H	0	-2.71714300	2.18948500	-0.76966600
C	-1	-7.06461900	4.52422200	-0.65602300
O	-1	-6.29171800	5.44084200	-0.42534100
N	-1	-8.40237100	4.57200900	-0.45490000
H	-1	-8.93735200	3.74072700	-0.69237600
C	-1	-9.07660500	5.76484000	0.02195800
H	-1	-9.58450700	5.57506600	0.97199800
H	-1	-8.32543400	6.53890500	0.16944400
H	-1	-9.81471500	6.11487600	-0.70551800
C	-1	-5.39276500	0.30101700	1.67363100
H	-1	-5.25645600	1.06019900	0.90104900
C	-1	-4.55142900	-0.89409600	1.28240600
O	-1	-3.32757300	-0.82777300	1.20268300
H	-1	-6.45552000	0.07323900	1.76691100
H	-1	-5.02435500	0.70222000	2.61915200
N	-1	-5.23217100	-2.03035800	1.01499700

H	-1	-6.23319700	-2.06494900	1.15058500
C	-1	-4.61050400	-3.25420100	0.62143100
H	-1	-3.88393200	-3.01931700	-0.16184000
C	0	-3.82083800	-3.86941900	1.81438100
H	0	-3.33366900	-3.03813700	2.32417800
H	0	-4.52779200	-4.32547700	2.51399400
C	0	-2.74751100	-4.86331500	1.38567300
H	0	-3.17707500	-5.77878200	0.97460200
H	0	-2.10503600	-4.40505400	0.63028300
S	0	-1.68512000	-5.29854700	2.81049900
C	0	-0.34637800	-6.14857100	1.92223200
H	0	-0.72599500	-7.02322300	1.38964500
H	0	0.14309200	-5.46824600	1.22420100
H	0	0.38267400	-6.47517400	2.66454200
C	-1	-5.61428700	-4.31314000	0.08683900
O	-1	-6.75122100	-4.38248700	0.52698700
N	-1	-5.09177800	-5.14722800	-0.84315900
H	-1	-4.18499300	-4.92181500	-1.22423600
C	-1	-5.84921900	-6.24350400	-1.42165800
H	-1	-6.80084900	-6.29967700	-0.89625700
H	-1	-6.04372500	-6.07047700	-2.48511300
H	-1	-5.31598600	-7.18923200	-1.30664300
Se	0	-0.18909800	-0.93948400	-0.63337100
Se	0	-0.34036300	-1.96615800	1.47020500
C	0	-1.31933400	-2.13752400	-1.64593200
C	0	-2.49941900	-1.62918300	-2.19014200
C	0	-3.31898800	-2.45585400	-2.95690400
C	0	-2.97022300	-3.78871900	-3.16751500
C	0	-1.79770800	-4.29822900	-2.60558100
C	0	-0.96682000	-3.47334500	-1.85072500
C	0	1.34753200	-2.91015400	1.61300000
C	0	1.70266000	-3.33981100	2.89547200
C	0	2.86631800	-4.08139400	3.08151900
C	0	3.68718700	-4.38959300	1.99726700
C	0	3.33442400	-3.94929800	0.72330300
C	0	2.16644100	-3.21411700	0.52672300

H	0	-2.78712100	-0.60298300	-2.00323100
H	0	-4.23592000	-2.05645700	-3.37454700
H	0	-3.60745900	-4.42841600	-3.76767800
H	0	-1.52170000	-5.33441000	-2.76682800
H	0	-0.05249900	-3.86655600	-1.42454500
H	0	1.07098700	-3.10166500	3.74379600
H	0	3.13395200	-4.41207000	4.07893500
H	0	4.59621200	-4.96064300	2.14553200
H	0	3.96896600	-4.17626800	-0.12618300
H	0	1.90341900	-2.86566800	-0.46418200

C) **Zwit**

E= -8828.086565 Ha

G= -8827.662040 Ha

C	-1	0.95780000	5.48603900	5.15744000
H	-1	1.47641100	6.21546000	5.78036500
C	-1	1.93981900	4.92324300	4.14905900
O	-1	3.05232400	4.54655600	4.46108700
H	-1	0.08342100	5.95294400	4.70022300
H	-1	0.62494700	4.67536300	5.81006700
N	-1	1.48450900	4.88841200	2.84544100
H	-1	0.49408400	5.03851900	2.70465000
C	-1	2.16814400	4.23956200	1.83952200
H	-1	3.20793800	4.16006400	2.17671400
C	0	1.72483200	2.80371900	1.49018200
H	0	2.40784800	2.44357100	0.71925500
H	0	1.88278500	2.18325200	2.37556500
C	0	0.31816200	2.59930000	1.01526600
N	0	-0.54433200	1.75145800	1.69951500
H	0	-0.30363300	1.22455700	2.52904100
C	0	-1.69271000	1.61226600	1.02892300
H	0	-2.48334100	0.90647000	1.25574000
N	0	-1.61694700	2.37070600	-0.05623000
C	0	-0.37935400	2.98969500	-0.09948600
H	0	-0.07983100	3.62905700	-0.91026900
C	-1	2.23529200	5.12780300	0.54336000

O	-1	2.35656800	4.63221400	-0.56416200
N	-1	2.18106200	6.46270800	0.77565500
H	-1	2.08935300	6.76416900	1.73231800
C	-1	2.30242100	7.44570700	-0.28500700
H	-1	2.37312800	6.90382900	-1.22654500
H	-1	1.42814600	8.10165400	-0.31316400
H	-1	3.20093400	8.05617900	-0.15684000
C	-1	10.47381100	3.06963000	-2.99722800
H	-1	11.38960100	3.63896900	-2.83886700
C	-1	10.28616200	2.11621500	-1.83168400
O	-1	10.48474400	2.45059300	-0.67986700
H	-1	10.51727100	2.56872300	-3.96637800
H	-1	9.64134900	3.77784400	-3.00456900
N	-1	9.89025000	0.84258800	-2.17718000
H	-1	9.56447800	0.69215900	-3.12107800
C	-1	9.40491100	-0.11893200	-1.15125400
H	-1	9.69719200	0.32100200	-0.19430500
C	0	7.89218000	-0.33706100	-1.20974300
H	0	7.60353800	-0.61972300	-2.22786100
H	0	7.66677600	-1.18994900	-0.56891100
C	0	7.09930600	0.88820100	-0.76675700
H	0	7.35872600	1.16903900	0.25768900
H	0	7.31540700	1.74846600	-1.40508700
S	0	5.28415400	0.68685400	-0.87822800
C	0	4.99782300	-0.62601500	0.35158800
H	0	3.91708200	-0.75324200	0.42939100
H	0	5.43816300	-1.57399900	0.04250400
H	0	5.39128900	-0.33400100	1.32757000
C	-1	10.17068100	-1.46565300	-1.28022500
O	-1	9.70603900	-2.49475600	-0.81643100
N	-1	11.37437800	-1.39037500	-1.89930000
H	-1	11.66859100	-0.48340500	-2.22414800
C	-1	12.26363900	-2.53119800	-2.01529500
H	-1	11.73347600	-3.40129300	-1.63188200
H	-1	12.53799900	-2.70788400	-3.05873700
H	-1	13.17552100	-2.38385900	-1.42912300

C	-1	-8.58399700	1.66726300	-4.04409800
H	-1	-9.50062300	2.12911800	-4.41455700
C	-1	-8.29246900	2.20642000	-2.66017900
O	-1	-9.11338400	2.12315300	-1.75001200
H	-1	-7.77695500	1.83806100	-4.75816100
H	-1	-8.76659300	0.59399400	-3.96154400
N	-1	-7.07481300	2.77538100	-2.48521400
H	-1	-6.42795300	2.81089300	-3.25772900
C	-1	-6.57476600	3.18418300	-1.17542600
H	-1	-6.91947600	2.42513800	-0.46501700
C	0	-5.03406100	3.24456700	-1.19705500
H	0	-4.75565400	4.12479600	-1.78073800
H	0	-4.71644400	3.45788200	-0.17476500
S	0	-4.13285700	1.78081400	-1.83726800
H	0	-2.39340400	2.31330000	-0.79936000
C	-1	-7.06534000	4.55168800	-0.64310700
O	-1	-6.29264900	5.46582600	-0.40193900
N	-1	-8.40420800	4.60001100	-0.45015700
H	-1	-8.93894600	3.77147100	-0.69754000
C	-1	-9.07947800	5.78959100	0.03330700
H	-1	-9.59283000	5.59239000	0.97889500
H	-1	-8.32812000	6.56135200	0.19151800
H	-1	-9.81313000	6.14682600	-0.69517800
C	-1	-5.41189000	0.30693800	1.65814100
H	-1	-5.27034100	1.07245700	0.89302600
C	-1	-4.56992700	-0.88626500	1.26185200
O	-1	-3.34564800	-0.82012900	1.18850700
H	-1	-6.47542200	0.07975400	1.74383700
H	-1	-5.04813500	0.69949600	2.60924900
N	-1	-5.25063100	-2.01901800	0.98086600
H	-1	-6.25251500	-2.05337400	1.11042000
C	-1	-4.62856700	-3.24018300	0.57995200
H	-1	-3.89739400	-2.99961300	-0.19723500
C	0	-3.84302300	-3.86621600	1.76978500
H	0	-3.35020500	-3.04045600	2.28334200
H	0	-4.55174700	-4.32166900	2.46785900

C	0	-2.77677700	-4.86515700	1.33390000
H	0	-3.21365900	-5.78260400	0.93530400
H	0	-2.14310100	-4.41385800	0.56616400
S	0	-1.69724300	-5.29223600	2.74789000
C	0	-0.38991200	-6.18145700	1.85159900
H	0	-0.79101800	-7.06817800	1.35603200
H	0	0.08568600	-5.52698400	1.11986000
H	0	0.35637300	-6.49151200	2.58373600
C	-1	-5.63030200	-4.29392400	0.03170400
O	-1	-6.77022300	-4.36459500	0.46401600
N	-1	-5.10408300	-5.12041900	-0.90348300
H	-1	-4.19451200	-4.89340300	-1.27626900
C	-1	-5.86046200	-6.20948700	-1.49605700
H	-1	-6.81443900	-6.26969900	-0.97559300
H	-1	-6.04831200	-6.02795100	-2.55826300
H	-1	-5.32850600	-7.15771800	-1.38543800
Se	0	-0.15499400	-0.98136100	-0.68531100
Se	0	-0.33374900	-2.02926900	1.40981200
C	0	-1.38559800	-2.08024300	-1.69113000
C	0	-2.57838700	-1.50400100	-2.12965500
C	0	-3.46192100	-2.25598600	-2.90248700
C	0	-3.16376500	-3.57920700	-3.22195000
C	0	-1.98009200	-4.15862600	-2.75919200
C	0	-1.08528600	-3.40908500	-1.99845400
C	0	1.33637000	-3.00597100	1.53575900
C	0	1.72402000	-3.40110100	2.81948600
C	0	2.87753300	-4.16184300	2.99255000
C	0	3.65670100	-4.52098900	1.89355000
C	0	3.27093100	-4.11594600	0.61729100
C	0	2.11132600	-3.36471500	0.43388400
H	0	-2.83339400	-0.48878000	-1.85250000
H	0	-4.38819300	-1.80410400	-3.23758500
H	0	-3.85101100	-4.15934600	-3.82733900
H	0	-1.74512300	-5.18869500	-3.00361300
H	0	-0.16155900	-3.85316500	-1.64870000
H	0	1.12644400	-3.12055100	3.67940600

H	0	3.17088500	-4.46573400	3.99122800
H	0	4.55914900	-5.10485400	2.03158800
H	0	3.87256500	-4.38417600	-0.24403300
H	0	1.82024000	-3.04757400	-0.55972300

D) **TCI**
E= -8828.090397 Ha
G= -8827.663536 Ha

C	-1	2.23282100	5.44214100	5.21505000
H	-1	2.81726000	6.19021700	5.75157500
C	-1	3.13815600	4.73819000	4.22370700
O	-1	4.24029100	4.32449100	4.52548700
H	-1	1.36705300	5.91909500	4.75185900
H	-1	1.88354600	4.71129400	5.94838200
N	-1	2.62635800	4.61906100	2.94616100
H	-1	1.64080100	4.81478500	2.83022100
C	-1	3.22960500	3.84731800	1.97632800
H	-1	4.27668300	3.73562700	2.27951400
C	0	2.66530400	2.42621200	1.77069400
H	0	3.33769200	1.90575100	1.08533000
H	0	2.71531800	1.90506300	2.72910000
C	0	1.27031800	2.30515100	1.23324100
N	0	0.37678500	1.38773000	1.77212500
H	0	0.57718600	0.76524500	2.54426500
C	0	-0.74020200	1.32123200	1.04594100
H	0	-1.55789400	0.63057700	1.22363700
N	0	-0.60715100	2.20623000	0.05900900
C	0	0.62732900	2.82571800	0.14251700
H	0	0.96087900	3.53545300	-0.59081800
C	-1	3.29048400	4.61603700	0.60541800
O	-1	3.33678400	4.02128700	-0.45811300
N	-1	3.32057800	5.96675500	0.72151100
H	-1	3.28662700	6.35376600	1.65079200
C	-1	3.45105700	6.84661200	-0.42515500
H	-1	3.45133000	6.22301800	-1.31748600
H	-1	2.61419100	7.54824900	-0.47784500

H	-1	4.38676100	7.41165300	-0.38498700
C	-1	11.24349600	1.77968400	-3.04850200
H	-1	12.19543500	2.30537600	-2.97531800
C	-1	11.05297700	0.94213900	-1.79753700
O	-1	11.31878100	1.36141300	-0.68781100
H	-1	11.21768900	1.19608900	-3.97088900
H	-1	10.45213300	2.53261500	-3.08663300
N	-1	10.57268400	-0.33087100	-2.01477900
H	-1	10.19890500	-0.54173800	-2.92885300
C	-1	10.07844100	-1.17023800	-0.89125700
H	-1	10.43561700	-0.66902000	0.01227800
C	0	8.56042500	-1.32617000	-0.89190000
H	0	8.23070400	-1.63130900	-1.89115300
H	0	8.33342800	-2.15246300	-0.21955300
C	0	7.81260800	-0.07229000	-0.45935100
H	0	8.08627400	0.20849800	0.56145900
H	0	8.04361500	0.77615000	-1.10824100
S	0	5.99277200	-0.24955200	-0.56564700
C	0	5.72796000	-1.70496200	0.49586500
H	0	4.65347000	-1.88040500	0.52317600
H	0	6.20802300	-2.59322100	0.08588300
H	0	6.08974300	-1.52076300	1.50989300
C	-1	10.76224400	-2.56638400	-0.92959900
O	-1	10.26124600	-3.52285300	-0.36038600
N	-1	11.94076100	-2.61553600	-1.59797200
H	-1	12.27079400	-1.75862900	-2.01206500
C	-1	12.75945400	-3.81264200	-1.64682000
H	-1	12.19857200	-4.61393900	-1.16882600
H	-1	12.97883500	-4.09376000	-2.68031300
H	-1	13.70227500	-3.66987000	-1.11059300
C	-1	-7.89007600	1.42126900	-3.24998400
H	-1	-8.79457000	1.90311300	-3.62478000
C	-1	-7.51031800	2.05862100	-1.93070500
O	-1	-8.29502300	2.10203800	-0.98647200
H	-1	-7.10596300	1.48243800	-4.00615900
H	-1	-8.12836400	0.37165200	-3.06672600

N	-1	-6.25657400	2.56747600	-1.85230300
H	-1	-5.64223400	2.49855400	-2.64876800
C	-1	-5.67896700	3.05617800	-0.60317300
H	-1	-6.03514100	2.38275500	0.18359700
C	0	-4.12384200	2.92445300	-0.59532500
H	0	-3.72027200	3.89153300	-0.30337000
H	0	-3.83612700	2.19986700	0.16369600
S	0	-3.25839800	2.44770100	-2.14069800
H	0	-1.30825200	2.36515100	-0.67731700
C	-1	-6.06985000	4.49107900	-0.17502500
O	-1	-5.23793200	5.37518900	-0.04411400
N	-1	-7.39449300	4.63469200	0.06333300
H	-1	-7.98452100	3.82108400	-0.09006300
C	-1	-7.98136900	5.89904900	0.46558900
H	-1	-8.46411200	5.81405900	1.44349100
H	-1	-7.18221300	6.63576800	0.52704700
H	-1	-8.72441700	6.23542800	-0.26330100
C	-1	-4.55829200	0.36827900	2.42621700
H	-1	-4.40710300	1.05588700	1.59199400
C	-1	-3.80162400	-0.90205600	2.10458500
O	-1	-2.57977600	-0.91484700	1.97993000
H	-1	-5.62818300	0.21247300	2.57145200
H	-1	-4.13306600	0.81851500	3.32477400
N	-1	-4.55561700	-2.01250000	1.95002100
H	-1	-5.55139800	-1.97641800	2.11979600
C	-1	-4.02000100	-3.29805900	1.63478300
H	-1	-3.31040800	-3.16844100	0.81248900
C	0	-3.23078100	-3.85907200	2.85056100
H	0	-2.55955800	-3.06097400	3.17187700
H	0	-3.92943600	-4.05950200	3.66827900
C	0	-2.40805800	-5.10339200	2.52848400
H	0	-3.03734000	-5.98899000	2.42739700
H	0	-1.85841800	-4.96787400	1.59307200
S	0	-1.19103500	-5.40394800	3.86186200
C	0	-0.36143800	-6.86871700	3.17231700
H	0	-1.06487500	-7.69509500	3.05123600

H	0	0.10285900	-6.63334400	2.21243100
H	0	0.41537800	-7.16571500	3.87866800
C	-1	-5.10125700	-4.33378400	1.21924100
O	-1	-6.22386600	-4.29979300	1.69869400
N	-1	-4.66213100	-5.26682800	0.34105800
H	-1	-3.75806800	-5.12663500	-0.08419300
C	-1	-5.50245200	-6.35596600	-0.12473300
H	-1	-6.43524500	-6.31501200	0.43455200
H	-1	-5.72503300	-6.25510100	-1.19094600
H	-1	-5.01979700	-7.32103500	0.04858400
Se	0	-2.66561500	0.04457400	-1.85965100
Se	0	-1.77091200	-2.56941600	-1.51036400
C	0	-4.41947200	-0.70444100	-2.17915300
C	0	-5.37632400	-0.70284500	-1.16370000
C	0	-6.58990900	-1.36024900	-1.34390500
C	0	-6.85538900	-2.01891000	-2.54350800
C	0	-5.91146400	-1.99771500	-3.56906500
C	0	-4.69553500	-1.33957300	-3.39136900
C	0	-0.22032400	-1.98368800	-0.53746300
C	0	0.03373600	-2.45330600	0.75795000
C	0	1.16546700	-2.03983900	1.45598600
C	0	2.05558800	-1.13238300	0.88135400
C	0	1.80563600	-0.64628200	-0.40189400
C	0	0.68049500	-1.06911500	-1.10602600
H	0	-5.16397400	-0.20833000	-0.22777000
H	0	-7.32400600	-1.36286500	-0.54627600
H	0	-7.79550400	-2.54112700	-2.67960000
H	0	-6.11615500	-2.50126700	-4.50731400
H	0	-3.95236800	-1.33910600	-4.17906000
H	0	-0.66273500	-3.13501200	1.22493200
H	0	1.34482600	-2.42675800	2.45384400
H	0	2.92953300	-0.80142400	1.42954100
H	0	2.48777000	0.06179200	-0.85917000
H	0	0.50167100	-0.69862500	-2.10847400

E) **TS2 img= -1094.2847 cm^{-1}**
E= -8828.061401 Ha
G= -8827.639429 Ha

C	-1	1.02980000	5.68840000	4.89300000
H	-1	1.55350000	6.45020000	5.47130000
C	-1	2.00820000	5.05860000	3.92140000
O	-1	3.11770000	4.69320000	4.25670000
H	-1	0.15920000	6.13340000	4.40750000
H	-1	0.69060000	4.92110000	5.59290000
N	-1	1.55340000	4.94870000	2.62160000
H	-1	0.70710000	5.36990000	2.38120000
C	-1	2.23300000	4.23540000	1.65760000
H	-1	3.27190000	4.16850000	1.99990000
C	0	1.89485200	2.75294900	1.43964700
H	0	2.71192800	2.34914700	0.84123900
H	0	1.96059100	2.26598300	2.41678600
C	0	0.62974700	2.33381200	0.78418500
N	0	-0.48000000	1.91807400	1.50258500
H	0	-0.58253600	1.97789100	2.50585600
C	0	-1.33789300	1.29538300	0.67007300
H	0	-2.22534400	0.76581200	0.98343000
N	0	-0.84582600	1.30918200	-0.56570100
C	0	0.37359200	1.95908000	-0.50661100
H	0	1.00779600	2.06399500	-1.36977200
C	-1	2.30740000	5.04330000	0.31020000
O	-1	2.42570000	4.48100000	-0.76530000
N	-1	2.26300000	6.39010000	0.46160000
H	-1	2.17300000	6.74940000	1.39820000
C	-1	2.39230000	7.30650000	-0.65630000
H	-1	2.45960000	6.70830000	-1.56340000
H	-1	1.52300000	7.96600000	-0.72490000
H	-1	3.29530000	7.91690000	-0.56420000
C	-1	10.53260000	2.71460000	-3.09120000
H	-1	11.45250000	3.28560000	-2.96640000
C	-1	10.33710000	1.83460000	-1.87050000
O	-1	10.53750000	2.23630000	-0.74070000

H	-1	10.57290000	2.15580000	-4.02830000
H	-1	9.70540000	3.42720000	-3.14210000
N	-1	9.93200000	0.54540000	-2.13890000
H	-1	9.60560000	0.34080000	-3.07240000
C	-1	9.43870000	-0.34880000	-1.05800000
H	-1	9.73390000	0.14550000	-0.12840000
C	0	7.93417600	-0.59209800	-1.12086500
H	0	7.65131900	-0.84461800	-2.14880800
H	0	7.74516900	-1.47796600	-0.51677000
C	0	7.08879100	0.56905600	-0.61833400
H	0	7.34463700	0.81211900	0.41674300
H	0	7.23942700	1.47055200	-1.21734700
S	0	5.29093200	0.22998600	-0.72463800
C	0	5.18798900	-1.32747000	0.21401500
H	0	4.13402300	-1.59037300	0.27710800
H	0	5.71201600	-2.13661100	-0.29392400
H	0	5.58638300	-1.20270100	1.22312700
C	-1	10.19470000	-1.70660000	-1.10390000
O	-1	9.72200000	-2.70240000	-0.57980000
N	-1	11.39920000	-1.67770000	-1.72540000
H	-1	11.70040000	-0.79410000	-2.10410000
C	-1	12.28010000	-2.83000000	-1.77150000
H	-1	11.74320000	-3.67140000	-1.33690000
H	-1	12.55370000	-3.07130000	-2.80220000
H	-1	13.19260000	-2.65430000	-1.19440000
C	-1	-8.53450000	1.39260000	-4.07180000
H	-1	-9.44740000	1.83810000	-4.47040000
C	-1	-8.23980000	2.01210000	-2.72260000
O	-1	-9.06190000	1.98990000	-1.80990000
H	-1	-7.72580000	1.51410000	-4.79400000
H	-1	-8.72510000	0.32770000	-3.92490000
N	-1	-7.01790000	2.58110000	-2.58090000
H	-1	-6.37050000	2.56570000	-3.35350000
C	-1	-6.51590000	3.06490000	-1.29660000
H	-1	-6.86670000	2.35250000	-0.54320000
C	0	-4.96116600	3.09402300	-1.32486900

H	0	-4.64667400	4.13052100	-1.44880400
H	0	-4.55332100	2.75108200	-0.37653300
S	0	-4.10061200	2.21703500	-2.68064700
H	0	-0.81554300	0.07748200	-1.21335100
C	-1	-6.99660000	4.46580000	-0.85030000
O	-1	-6.21710000	5.38660000	-0.66240000
N	-1	-8.33510000	4.53530000	-0.66100000
H	-1	-8.87580000	3.69730000	-0.85850000
C	-1	-9.00180000	5.75680000	-0.25080000
H	-1	-9.51720000	5.62070000	0.70440000
H	-1	-8.24480000	6.53110000	-0.13860000
H	-1	-9.73230000	6.07490000	-1.00030000
C	-1	-5.37620000	0.35550000	1.70630000
H	-1	-5.22800000	1.07140000	0.89500000
C	-1	-4.54300000	-0.86580000	1.38120000
O	-1	-3.31790000	-0.81370000	1.30760000
H	-1	-6.44130000	0.14130000	1.80350000
H	-1	-5.01000000	0.80150000	2.63150000
N	-1	-5.23170000	-2.00860000	1.16970000
H	-1	-6.23390000	-2.02770000	1.30020000
C	-1	-4.61830000	-3.25700000	0.84400000
H	-1	-3.88530000	-3.06780000	0.05430000
C	0	-3.87415300	-3.81370900	2.09174700
H	0	-3.32242400	-2.97980700	2.52616800
H	0	-4.62547300	-4.13117200	2.82161700
C	0	-2.89847900	-4.94642700	1.79535000
H	0	-3.40695400	-5.83333700	1.41490100
H	0	-2.16069000	-4.62696500	1.05488800
S	0	-1.99612400	-5.39823700	3.32432500
C	0	-0.62611000	-6.34416800	2.59207200
H	0	-0.99804000	-7.20504100	2.03278400
H	0	-0.02503400	-5.70661900	1.94087700
H	0	-0.00302800	-6.69986000	3.41427300
C	-1	-5.62780000	-4.33370000	0.35910000
O	-1	-6.76850000	-4.36970000	0.79350000
N	-1	-5.10710000	-5.21900000	-0.52410000

H	-1	-4.19570000	-5.02160000	-0.90890000
C	-1	-5.87120000	-6.33610000	-1.05080000
H	-1	-6.82600000	-6.35780000	-0.52860000
H	-1	-6.05710000	-6.21760000	-2.12220000
H	-1	-5.34640000	-7.27990000	-0.88260000
Se	0	-3.69144000	0.16779800	-1.93590400
Se	0	-0.40473200	-1.54719100	-1.45659600
C	0	-5.27815500	-0.86668100	-2.32938800
C	0	-6.48454900	-0.63175300	-1.66687800
C	0	-7.56934900	-1.47715200	-1.87413000
C	0	-7.44469200	-2.58008900	-2.71947500
C	0	-6.23765000	-2.82295100	-3.37113000
C	0	-5.15592400	-1.96186100	-3.18766100
C	0	0.32840000	-1.54545600	0.33431400
C	0	-0.38759400	-2.11112100	1.39359600
C	0	0.10497000	-2.04777500	2.69523000
C	0	1.31213400	-1.40135400	2.95807600
C	0	2.02818000	-0.83114700	1.90713200
C	0	1.54454200	-0.90930100	0.60163100
H	0	-6.57134600	0.19818000	-0.98093000
H	0	-8.50516200	-1.28337400	-1.36315300
H	0	-8.28453200	-3.24967100	-2.86337700
H	0	-6.13727100	-3.67742700	-4.03067800
H	0	-4.21912800	-2.14199500	-3.70119500
H	0	-1.35217600	-2.55584400	1.20292500
H	0	-0.46219700	-2.50228600	3.49943200
H	0	1.69404400	-1.34629300	3.97156100
H	0	2.96780000	-0.32580400	2.09898500
H	0	2.10220600	-0.46373400	-0.21169000

D) **PC**
E= -8828.082478 Ha
G= -8827.662003 Ha

C	-1	1.99959200	5.17031300	5.16897500
H	-1	2.57070100	5.88998300	5.75631500
C	-1	2.92003200	4.54386600	4.14023000

O	-1	4.02606000	4.12635100	4.42185500
H	-1	1.12996000	5.66452700	4.73187800
H	-1	1.65631900	4.38910700	5.85145800
N	-1	2.41706700	4.50058900	2.85435400
H	-1	1.42957300	4.69012800	2.74494300
C	-1	3.03625500	3.80116800	1.84074500
H	-1	4.08305900	3.68398600	2.14292800
C	0	2.46746800	2.40283500	1.55755000
H	0	2.98880000	2.00213700	0.68456900
H	0	2.70705300	1.75841300	2.40899900
C	0	0.99028100	2.39907300	1.33624400
N	0	0.22843600	1.26891400	1.54173500
H	0	0.57999200	0.35904900	1.81065700
C	0	-1.07207900	1.57388200	1.27248400
H	0	-1.84825900	0.82896200	1.33719100
N	0	-1.19659400	2.83486900	0.91334400
C	0	0.08308000	3.35895600	0.94573700
H	0	0.27412700	4.38429900	0.66789800
C	-1	3.09425300	4.65730100	0.52263900
O	-1	3.15460100	4.13302100	-0.57666100
N	-1	3.10510400	5.99800400	0.72564900
H	-1	3.06064700	6.32381000	1.67768900
C	-1	3.22986600	6.95162100	-0.36112400
H	-1	3.24368900	6.38690800	-1.29172600
H	-1	2.38373900	7.64395600	-0.37392700
H	-1	4.15747900	7.52534700	-0.27861100
C	-1	11.10572400	2.16889200	-3.25492500
H	-1	12.04992800	2.70143600	-3.14189600
C	-1	10.91973400	1.24997600	-2.06173700
O	-1	11.17358600	1.60033600	-0.92562400
H	-1	11.09309500	1.64568200	-4.21312300
H	-1	10.30431500	2.91207700	-3.24960100
N	-1	10.45824700	-0.01264600	-2.36355100
H	-1	10.09247800	-0.16914400	-3.29173400
C	-1	9.96938200	-0.92941000	-1.29985800
H	-1	10.31448300	-0.48258400	-0.36343800

C	0	8.44497500	-1.05342000	-1.29043500
H	0	8.09566800	-1.39810200	-2.26944200
H	0	8.18330700	-1.82784100	-0.56831300
C	0	7.77252000	0.27031500	-0.93359300
H	0	8.10825700	0.62545500	0.04447300
H	0	8.02808000	1.04288900	-1.66253000
S	0	5.94602400	0.23175900	-0.93725500
C	0	5.62201500	-0.83368900	0.50191800
H	0	4.54487600	-0.81382600	0.66826100
H	0	5.93545700	-1.86284800	0.32197700
H	0	6.12222700	-0.44199000	1.39028300
C	-1	10.67249800	-2.31077200	-1.42319000
O	-1	10.18145700	-3.30856400	-0.92022200
N	-1	11.85521300	-2.30100700	-2.08597000
H	-1	12.17576700	-1.41484900	-2.44184800
C	-1	12.69055900	-3.48142000	-2.20646200
H	-1	12.13809100	-4.31928500	-1.78468500
H	-1	12.91955500	-3.69237100	-3.25447900
H	-1	13.62833100	-3.36094000	-1.65608300
C	-1	-8.01968400	1.56867700	-3.60254100
H	-1	-8.92861700	2.06155800	-3.95136100
C	-1	-7.65610000	2.12469600	-2.24254600
O	-1	-8.44657900	2.09663700	-1.30255300
H	-1	-7.23228400	1.68894400	-4.34813200
H	-1	-8.24454300	0.50634100	-3.48878400
N	-1	-6.40971300	2.64374500	-2.12341300
H	-1	-5.79029000	2.63502300	-2.91873800
C	-1	-5.84635400	3.05931000	-0.84071400
H	-1	-6.19723200	2.33131100	-0.10241600
C	0	-4.29325300	3.05246000	-0.93383100
H	0	-3.96181900	4.07761300	-1.10482300
H	0	-3.83167500	2.74536600	0.00187700
S	0	-3.50438900	2.14340300	-2.31069900
H	0	-1.33839700	-1.63089200	0.09236500
C	-1	-6.25913500	4.45819800	-0.32551400
O	-1	-5.44002400	5.34266000	-0.13135500

N	-1	-7.58681600	4.56815100	-0.08606700
H	-1	-8.16473400	3.75831800	-0.29534000
C	-1	-8.19325300	5.79598900	0.39297000
H	-1	-8.68022400	5.64169700	1.36023400
H	-1	-7.40465300	6.53782800	0.50688900
H	-1	-8.93677300	6.16870800	-0.31753000
C	-1	-4.70555300	0.19695100	2.01624700
H	-1	-4.55879300	0.93785700	1.22751500
C	-1	-3.92986200	-1.03972200	1.61620800
O	-1	-2.70695500	-1.02902300	1.50100700
H	-1	-5.77390700	0.01741800	2.14347500
H	-1	-4.29146400	0.59353400	2.94386600
N	-1	-4.66738700	-2.14833000	1.38702200
H	-1	-5.66451300	-2.13659600	1.55239200
C	-1	-4.11215000	-3.40439800	0.99332800
H	-1	-3.40036200	-3.21108100	0.18538400
C	0	-3.32273900	-4.04551800	2.17620300
H	0	-2.91318900	-3.23204700	2.77441300
H	0	-4.02152700	-4.59726000	2.81241200
C	0	-2.16972600	-4.93303400	1.72382400
H	0	-2.51705800	-5.77217500	1.11761800
H	0	-1.46246400	-4.34669200	1.13237700
S	0	-1.26805500	-5.58409100	3.17917500
C	0	0.33287000	-5.95125700	2.39790700
H	0	0.22621900	-6.71285700	1.62264400
H	0	0.76632800	-5.04460900	1.97532200
H	0	0.99331500	-6.33157700	3.17866800
C	-1	-5.17704700	-4.42473600	0.50480200
O	-1	-6.30265900	-4.43670200	0.97810600
N	-1	-4.72019200	-5.29332900	-0.42877600
H	-1	-3.81580400	-5.11392200	-0.83831900
C	-1	-5.54286800	-6.36126500	-0.96918100
H	-1	-6.47924700	-6.36892500	-0.41445500
H	-1	-5.76085700	-6.19486300	-2.02809900
H	-1	-5.04797400	-7.32897500	-0.85525700
Se	0	-3.18740400	0.07657800	-1.58773300

Se	0	-0.11757400	-2.06884700	-0.60739100
C	0	-4.80200800	-0.89644900	-2.01979100
C	0	-5.99757000	-0.64684200	-1.34282900
C	0	-7.11106000	-1.44530400	-1.58162800
C	0	-7.02746400	-2.51408600	-2.47469400
C	0	-5.83214100	-2.77008500	-3.14243700
C	0	-4.72017900	-1.95636400	-2.92561100
C	0	0.88302200	-2.34051500	1.01439800
C	0	0.29353300	-2.18084600	2.27266100
C	0	1.05554200	-2.38611100	3.42192100
C	0	2.39613700	-2.75213700	3.32864800
C	0	2.97828200	-2.91056400	2.07120900
C	0	2.22973200	-2.70368400	0.91532300
H	0	-6.05455600	0.15664500	-0.62267300
H	0	-8.03801400	-1.24179600	-1.05852500
H	0	-7.89107000	-3.14620000	-2.64463500
H	0	-5.76462300	-3.59651500	-3.84074800
H	0	-3.79342100	-2.14379700	-3.45477200
H	0	-0.75087600	-1.90824800	2.35250200
H	0	0.58848800	-2.26953800	4.39313600
H	0	2.98212500	-2.91504400	4.22541900
H	0	4.02130400	-3.19335300	1.98674700
H	0	2.69213100	-2.82307600	-0.05795000

Table S13: Cluster 2 at 46.2 ns; Optimized coordinates at SMD(water)-D3BJ-B3LYP/6-311G(d,p),ccPVTZ. Constrained atoms (*first column* = *-1*): 1,2,3,4,5,6,7,8,9,10,22,23,24,25,26,27,28,29,30,31,32,33,34,35,36,37,38,39,51,52,53,54,55,56,57,58,59,60,61,62,63,64,65,66,67,68,74,75,76,77,78,79,80, 81,82,83,84,85,86,87,88,89,90,91,103,104,105,106,107,108,109,110

A) **RC**
E= -8828.0042300 Ha
G= -8827.5834940 Ha

H	-1	1.07545100	2.12946500	-6.67512000
C	-1	0.03218300	2.31831300	-6.92796800
H	-1	0.02140800	3.34866000	-7.28535600
H	-1	-0.29338600	1.63851800	-7.71539600
C	-1	-0.97993900	2.14241900	-5.80487400
O	-1	-1.93174900	2.94503800	-5.68497000
N	-1	-0.79447700	1.24943800	-4.85207400
H	-1	-0.03258200	0.59797800	-4.97436900
C	-1	-1.73821300	1.04641800	-3.80776500
H	-1	-2.74445400	0.90958200	-4.20445400
C	0	-1.40389500	-0.20116900	-2.96637200
H	0	-2.20221900	-0.30700400	-2.23173100
H	0	-1.44723700	-1.07322700	-3.62338000
C	0	-0.09115200	-0.16059000	-2.25770800
N	0	1.07829400	-0.67978100	-2.78652000
H	0	1.16863900	-1.13254500	-3.68638100
C	0	2.07189200	-0.49381100	-1.87976000
H	0	3.07886100	-0.83977000	-2.03908900
N	0	1.61718900	0.10988300	-0.79595600
C	0	0.26920300	0.32726600	-1.02644000
H	0	-0.36402000	0.80686600	-0.29840400
C	-1	-1.98346800	2.25230000	-2.93026100
O	-1	-3.02968900	2.28483000	-2.27664800
N	-1	-1.14341500	3.29999700	-2.95962300
H	-1	-0.33060800	3.13308800	-3.53494900
C	-1	-1.28843700	4.62920500	-2.31368500
H	-1	-1.07805100	4.58007600	-1.24560200
H	-1	-0.52420200	5.31233600	-2.68414200
H	-1	-2.22265300	5.12581800	-2.57568500
H	-1	-11.29180700	1.96890900	0.51883800
C	-1	-11.19828400	1.64200400	-0.51677500
H	-1	-12.16435900	1.72072700	-1.01356700
H	-1	-10.59391700	2.37359500	-1.05298700
C	-1	-10.77303000	0.14848500	-0.68113900
O	-1	-10.98705100	-0.50780300	-1.68024300
N	-1	-10.02829100	-0.30911800	0.39539100

H	-1	-9.77864700	0.12524600	1.27314100
C	-1	-9.59563900	-1.74873600	0.26603500
H	-1	-9.01908500	-2.01671100	-0.61955300
C	0	-8.62866000	-2.05101300	1.43427400
H	0	-9.05607200	-1.68517300	2.37276000
H	0	-8.51865300	-3.13305400	1.52226800
C	0	-7.25570300	-1.42809600	1.17518500
H	0	-6.80747900	-1.88355600	0.28929000
H	0	-7.32968900	-0.35457100	0.98868900
S	0	-6.04855300	-1.66784600	2.52412800
C	0	-6.54421500	-0.31173100	3.63130800
H	0	-6.41583800	0.65037500	3.13257900
H	0	-7.57842400	-0.42924400	3.95737900
H	0	-5.89045000	-0.35183400	4.50358800
C	-1	-10.74589100	-2.82886100	0.28026400
O	-1	-10.58633600	-4.02143900	-0.04410700
N	-1	-12.02084600	-2.43146000	0.52560600
H	-1	-12.30519000	-1.47744400	0.69674200
C	-1	-13.24235700	-3.28487300	0.39785600
H	-1	-12.97413100	-4.31388000	0.63776300
H	-1	-13.89681100	-2.92301600	1.19085000
H	-1	-13.65756300	-3.25622500	-0.60957000
H	-1	7.26842100	1.36912600	4.30733600
C	-1	8.22905500	1.62649000	3.86127300
H	-1	8.50753000	2.63284000	4.17144900
H	-1	8.92147500	0.82974600	4.13301100
C	-1	8.16123200	1.59740700	2.32566100
O	-1	9.17091500	1.62589900	1.70293100
N	-1	7.00431600	1.51197100	1.72155200
H	-1	6.25524700	1.45544500	2.39668500
C	-1	6.83834900	1.27660000	0.30935400
H	-1	7.42925100	0.37938800	0.12507900
C	0	5.35053900	1.01216700	-0.02930100
H	0	4.84707200	1.95526200	-0.23187600
H	0	5.30580100	0.38703000	-0.91957600
S	0	4.42346400	0.18770700	1.33225700

H	0	3.25050800	0.15022900	0.64923200
C	-1	7.27253600	2.47969300	-0.53247500
O	-1	7.07323800	3.66449500	-0.12847900
N	-1	7.95737700	2.22864800	-1.64408400
H	-1	8.15303000	1.25568200	-1.82823300
C	-1	8.74656800	3.20221900	-2.34022600
H	-1	8.80948600	3.08525300	-3.42156300
H	-1	8.20770300	4.15043900	-2.34519700
H	-1	9.77354700	3.41581200	-2.04405500
H	-1	6.70721200	-2.66714300	-2.73997700
C	-1	5.62388500	-2.58850200	-2.64822100
H	-1	5.32229400	-1.54274500	-2.72497400
H	-1	5.07166900	-3.03044000	-3.47766500
C	-1	5.01567700	-3.09882500	-1.30802000
O	-1	4.27238500	-2.39542300	-0.72949900
N	-1	5.33604200	-4.28098000	-0.77955300
H	-1	6.07187500	-4.83577800	-1.19320300
C	-1	4.70895800	-4.78655100	0.45245800
H	-1	4.55284200	-3.94713000	1.12947700
C	0	3.29183300	-5.34406000	0.15928100
H	0	2.77539200	-5.46470800	1.11676000
H	0	2.74045300	-4.58052600	-0.39176200
C	0	3.20858700	-6.68656200	-0.56654900
H	0	3.77416200	-7.45700900	-0.03828400
H	0	2.16424700	-7.00724100	-0.58729300
S	0	3.83805300	-6.75426400	-2.28653300
C	0	2.80470600	-5.48228800	-3.07302900
H	0	1.74519300	-5.69977600	-2.92397500
H	0	3.03198000	-4.48688300	-2.69149700
H	0	3.02573200	-5.51002800	-4.14089400
C	-1	5.46928600	-5.78736100	1.29038100
O	-1	6.27073900	-6.49107700	0.64721300
N	-1	5.26118300	-5.79248100	2.56816300
H	-1	4.72782800	-5.05117800	3.00114100
C	-1	5.79562000	-6.86908900	3.37536200
H	-1	6.73567300	-7.18600800	2.92328600

H	-1	5.98810400	-6.51307400	4.38744300
H	-1	5.09301700	-7.70151900	3.41513900
Se	0	0.51702200	3.13189100	3.27561700
Se	0	-0.66298300	1.24782400	2.55244700
C	0	1.64634600	3.52313500	1.75124100
C	0	2.97081200	3.88051100	2.01200100
C	0	3.81369000	4.22397700	0.95725300
C	0	3.34323100	4.19502900	-0.35456500
C	0	2.02181800	3.82783100	-0.60915500
C	0	1.16800400	3.49770000	0.44118600
C	0	-2.26969600	1.98614700	1.75145600
C	0	-3.31519500	1.08371000	1.53460600
C	0	-4.49604100	1.52441500	0.94106300
C	0	-4.64317800	2.86095600	0.57314900
C	0	-3.59537500	3.75331200	0.79014100
C	0	-2.40638500	3.32085700	1.37547100
H	0	3.34674200	3.87736100	3.02805300
H	0	4.84703300	4.47677300	1.15806400
H	0	4.00873700	4.44341800	-1.17274400
H	0	1.65025000	3.79400100	-1.62668600
H	0	0.14744300	3.20398900	0.23827900
H	0	-3.21903200	0.04579200	1.83260400
H	0	-5.30052200	0.81803800	0.77629100
H	0	-5.56205800	3.20094500	0.11111900
H	0	-3.69483300	4.79171500	0.49643800
H	0	-1.59878900	4.02140300	1.54490500

B) **TS1 (proton transfer Cys145-His41) img= -1202.5167 cm^{-1}**

E= -8827.995869 Ha

G= -8827.575083 Ha

H	1.008936000	-2.307137000	6.093371000
C	-0.017107000	-2.580313000	6.339571000
H	0.037549000	-3.639034000	6.595653000
H	-0.366917000	-1.999480000	7.193049000
C	-1.057612000	-2.357237000	5.251337000

O	-1.963873000	-3.199183000	5.066008000
N	-0.940740000	-1.367617000	4.387287000
H	-0.215251000	-0.687334000	4.562581000
C	-1.912907000	-1.121305000	3.378939000
H	-2.918181000	-1.082333000	3.799644000
C	-1.555052000	0.171987000	2.603438000
H	-2.302541000	0.304598000	1.822183000
H	-1.630961000	1.022789000	3.283940000
C	-0.184742000	0.148370000	1.984571000
N	0.931827000	0.687825000	2.612327000
H	0.932071000	1.160664000	3.508080000
C	2.014060000	0.493765000	1.835963000
H	3.008432000	0.812041000	2.093130000
N	1.654576000	-0.131380000	0.725967000
C	0.291449000	-0.363537000	0.803926000
H	-0.246815000	-0.870369000	0.022872000
C	-2.104581000	-2.249679000	2.392174000
O	-3.158692000	-2.280723000	1.751354000
N	-1.205571000	-3.244164000	2.309587000
H	-0.393361000	-3.085778000	2.888340000
C	-1.285293000	-4.512069000	1.539851000
H	-1.097889000	-4.347458000	0.479106000
H	-0.476976000	-5.180878000	1.833058000
H	-2.185117000	-5.084877000	1.764054000
H	-11.474099000	-2.183937000	-0.898522000
C	-11.380915000	-1.952946000	0.162641000
H	-12.331841000	-2.135559000	0.661332000
H	-10.726133000	-2.695831000	0.618042000
C	-11.039024000	-0.459691000	0.465552000
O	-11.272344000	0.084031000	1.526051000
N	-10.341138000	0.142011000	-0.570711000
H	-10.082758000	-0.190753000	-1.489385000
C	-9.989300000	1.585474000	-0.307958000
H	-9.413360000	1.800695000	0.592269000
C	-9.083919000	2.064597000	-1.465049000
H	-9.568916000	1.841484000	-2.420013000

H	-8.981301000	3.148553000	-1.393226000
C	-7.699044000	1.422004000	-1.392665000
H	-7.198869000	1.719273000	-0.467960000
H	-7.758385000	0.331619000	-1.398191000
S	-6.570569000	1.919419000	-2.739888000
C	-7.288457000	0.974056000	-4.120445000
H	-7.282226000	-0.093269000	-3.892686000
H	-8.305008000	1.302014000	-4.339415000
H	-6.661635000	1.156657000	-4.994251000
C	-11.199679000	2.592558000	-0.203378000
O	-11.102890000	3.755845000	0.232886000
N	-12.453960000	2.146346000	-0.470358000
H	-12.686220000	1.198095000	-0.729512000
C	-13.719859000	2.910346000	-0.245568000
H	-13.515347000	3.971588000	-0.388028000
H	-14.366615000	2.588409000	-1.061750000
H	-14.114630000	2.760865000	0.759388000
H	6.950467000	-0.134492000	-4.839294000
C	7.932100000	-0.376588000	-4.432093000
H	8.262153000	-1.330447000	-4.841627000
H	8.572774000	0.481832000	-4.633934000
C	7.890241000	-0.498960000	-2.900160000
O	8.910892000	-0.527704000	-2.295585000
N	6.741352000	-0.539952000	-2.276425000
H	5.978287000	-0.463045000	-2.933647000
C	6.587502000	-0.451332000	-0.846192000
H	7.129233000	0.457270000	-0.583176000
C	5.136637000	-0.070963000	-0.445966000
H	5.092544000	-0.110372000	0.644694000
H	5.003579000	0.972936000	-0.731416000
S	3.762828000	-1.064406000	-1.155816000
H	2.544778000	-0.542846000	-0.133316000
C	7.104891000	-1.701977000	-0.130114000
O	6.966563000	-2.852229000	-0.644431000
N	7.794005000	-1.518939000	0.992057000
H	7.936894000	-0.558317000	1.267091000

C	8.650014000	-2.506752000	1.580920000
H	8.725491000	-2.490551000	2.667644000
H	8.166516000	-3.481081000	1.500715000
H	9.682070000	-2.630265000	1.252849000
H	6.285349000	3.167250000	2.571954000
C	5.206860000	3.034283000	2.486193000
H	4.966940000	1.970122000	2.465596000
H	4.645193000	3.361418000	3.361477000
C	4.546532000	3.634141000	1.209471000
O	3.834386000	2.947112000	0.574725000
N	4.789110000	4.878349000	0.794003000
H	5.499240000	5.433176000	1.250337000
C	4.112119000	5.462059000	-0.375465000
H	3.992235000	4.683730000	-1.128588000
C	2.671434000	5.906803000	-0.011982000
H	2.130245000	6.081466000	-0.947141000
H	2.175347000	5.066839000	0.477026000
C	2.527789000	7.172780000	0.832124000
H	3.029297000	8.020434000	0.360385000
H	1.466871000	7.423629000	0.906439000
S	3.204716000	7.128407000	2.534320000
C	2.274388000	5.730203000	3.229656000
H	1.199456000	5.897851000	3.137751000
H	2.542156000	4.790672000	2.746214000
H	2.535248000	5.670314000	4.287170000
C	4.798753000	6.581544000	-1.121875000
O	5.569981000	7.266175000	-0.423523000
N	4.567844000	6.696969000	-2.390504000
H	4.070149000	5.970575000	-2.886607000
C	5.025198000	7.875559000	-3.096217000
H	5.953504000	8.202317000	-2.627190000
H	5.219596000	7.630183000	-4.140297000
H	4.275474000	8.665257000	-3.046581000
Se	1.433598000	-4.849399000	-1.720292000
Se	0.858280000	-2.760181000	-2.629319000
C	2.347098000	-4.336012000	-0.092610000

C	3.605254000	-4.884551000	0.159825000
C	4.272512000	-4.559079000	1.340306000
C	3.688761000	-3.688839000	2.260451000
C	2.434549000	-3.138957000	1.995499000
C	1.763933000	-3.462254000	0.820499000
C	-1.006964000	-2.522112000	-2.158314000
C	-1.472802000	-1.203648000	-2.150852000
C	-2.812600000	-0.943846000	-1.867994000
C	-3.685664000	-1.991653000	-1.582165000
C	-3.214629000	-3.303503000	-1.593336000
C	-1.880777000	-3.574544000	-1.892282000
H	4.070333000	-5.542249000	-0.564367000
H	5.254282000	-4.976062000	1.531264000
H	4.213987000	-3.432104000	3.172886000
H	1.978134000	-2.450482000	2.697460000
H	0.802867000	-3.021318000	0.605723000
H	-0.792485000	-0.383888000	-2.352069000
H	-3.168102000	0.080040000	-1.858819000
H	-4.722522000	-1.786107000	-1.345230000
H	-3.886025000	-4.123639000	-1.367500000
H	-1.522843000	-4.596181000	-1.905984000

C) **Ion-pair (Zwit)**

E= -8828.0005060 Ha

G= -8827.5734140 Ha

H	0.917259000	-2.586109000	6.166408000
C	-0.116830000	-2.838889000	6.400430000
H	-0.087079000	-3.899778000	6.651598000
H	-0.463408000	-2.255065000	7.253186000
C	-1.140858000	-2.588630000	5.302521000
O	-2.062546000	-3.410430000	5.103361000
N	-0.994142000	-1.597423000	4.444867000
H	-0.256456000	-0.933402000	4.631265000
C	-1.950209000	-1.325763000	3.427675000
H	-2.958842000	-1.267767000	3.838020000

C	-1.612319000	-0.010264000	2.683742000
H	-2.396288000	0.150415000	1.944878000
H	-1.659910000	0.813306000	3.399610000
C	-0.285882000	0.026790000	1.979357000
N	0.888665000	0.468007000	2.584338000
H	0.971226000	0.794736000	3.540105000
C	1.899238000	0.427314000	1.712740000
H	2.913571000	0.728462000	1.904826000
N	1.419341000	-0.008736000	0.553500000
C	0.066857000	-0.273600000	0.692414000
H	-0.533940000	-0.642258000	-0.118498000
C	-2.155026000	-2.444969000	2.433140000
O	-3.202715000	-2.450698000	1.781159000
N	-1.276224000	-3.457696000	2.354840000
H	-0.467044000	-3.319268000	2.942882000
C	-1.374339000	-4.719825000	1.577772000
H	-1.172312000	-4.553943000	0.519913000
H	-0.583359000	-5.406905000	1.875972000
H	-2.288288000	-5.274710000	1.789573000
H	-11.485759000	-2.166184000	-0.955159000
C	-11.398992000	-1.942451000	0.108102000
H	-12.358760000	-2.107516000	0.595852000
H	-10.764773000	-2.701167000	0.566494000
C	-11.029118000	-0.458222000	0.422288000
O	-11.262207000	0.085029000	1.483079000
N	-10.307852000	0.133806000	-0.603484000
H	-10.046792000	-0.199764000	-1.521107000
C	-9.928652000	1.568244000	-0.329606000
H	-9.357889000	1.766867000	0.577706000
C	-8.971544000	2.019903000	-1.458629000
H	-9.368998000	1.709318000	-2.429644000
H	-8.920827000	3.109907000	-1.458038000
C	-7.568541000	1.452725000	-1.228359000
H	-7.141308000	1.890758000	-0.323622000
H	-7.591042000	0.370106000	-1.087763000
S	-6.375755000	1.800361000	-2.567397000

C	-6.765693000	0.426507000	-3.695986000
H	-6.560354000	-0.530647000	-3.213581000
H	-7.807538000	0.467626000	-4.016571000
H	-6.120871000	0.532317000	-4.569330000
C	-11.118706000	2.599998000	-0.232512000
O	-11.002190000	3.758824000	0.210746000
N	-12.379164000	2.181554000	-0.514920000
H	-12.628483000	1.239683000	-0.781389000
C	-13.631088000	2.970852000	-0.299462000
H	-13.402892000	4.028256000	-0.434287000
H	-14.275772000	2.666609000	-1.124028000
H	-14.039517000	2.824727000	0.700517000
H	7.018373000	-0.484697000	-4.692036000
C	7.990354000	-0.749367000	-4.275829000
H	8.304667000	-1.707911000	-4.686804000
H	8.650971000	0.096395000	-4.466509000
C	7.929730000	-0.878407000	-2.745071000
O	8.943099000	-0.931571000	-2.129992000
N	6.773697000	-0.898344000	-2.133628000
H	6.019409000	-0.802178000	-2.798402000
C	6.606610000	-0.813586000	-0.704646000
H	7.164443000	0.082126000	-0.431279000
C	5.086713000	-0.626176000	-0.303268000
H	4.756271000	-1.544047000	0.183992000
H	5.063476000	0.162653000	0.449672000
S	3.875277000	-0.243142000	-1.636730000
H	2.046126000	-0.164025000	-0.277466000
C	7.090077000	-2.078347000	0.010343000
O	6.933133000	-3.222883000	-0.511330000
N	7.770960000	-1.915374000	1.140603000
H	7.931023000	-0.959337000	1.422078000
C	8.599807000	-2.923848000	1.733291000
H	8.664101000	-2.914610000	2.820816000
H	8.096879000	-3.887399000	1.642993000
H	9.632463000	-3.067386000	1.415418000
H	6.344173000	2.793597000	2.728794000

C	5.264103000	2.683736000	2.631052000
H	5.002173000	1.624964000	2.602446000
H	4.700180000	3.018264000	3.502082000
C	4.630035000	3.303637000	1.350598000
O	3.910410000	2.634864000	0.704885000
N	4.903009000	4.544515000	0.944127000
H	5.619737000	5.082041000	1.410736000
C	4.250810000	5.148091000	-0.229339000
H	4.122624000	4.376185000	-0.987687000
C	2.815859000	5.620921000	0.120957000
H	2.287908000	5.810198000	-0.818952000
H	2.297569000	4.789422000	0.601293000
C	2.689211000	6.886196000	0.968752000
H	3.212018000	7.725491000	0.505271000
H	1.632854000	7.157959000	1.034118000
S	3.348668000	6.821779000	2.677119000
C	2.382556000	5.441116000	3.358564000
H	1.312326000	5.629753000	3.253162000
H	2.638346000	4.497089000	2.877350000
H	2.628516000	5.374966000	4.419282000
C	4.968607000	6.256585000	-0.962720000
O	5.746574000	6.921405000	-0.252799000
N	4.753606000	6.383106000	-2.233086000
H	4.246081000	5.669787000	-2.738122000
C	5.242994000	7.555306000	-2.927870000
H	6.172926000	7.860171000	-2.447461000
H	5.443247000	7.311067000	-3.971110000
H	4.509495000	8.360321000	-2.882013000
Se	1.560096000	-4.039339000	-2.174748000
Se	0.662091000	-1.922407000	-2.632326000
C	2.300815000	-3.786754000	-0.403271000
C	3.611470000	-4.208002000	-0.171623000
C	4.160684000	-4.078104000	1.103605000
C	3.419808000	-3.499712000	2.131899000
C	2.116890000	-3.067064000	1.887542000
C	1.548186000	-3.227361000	0.626833000

C	-1.228903000	-2.088777000	-2.220056000
C	-1.972602000	-0.908659000	-2.335597000
C	-3.333985000	-0.914220000	-2.037675000
C	-3.956125000	-2.089889000	-1.618649000
C	-3.209284000	-3.261205000	-1.507904000
C	-1.848632000	-3.266707000	-1.812380000
H	4.206756000	-4.613370000	-0.980266000
H	5.179882000	-4.399159000	1.278276000
H	3.857864000	-3.377264000	3.115279000
H	1.542807000	-2.604166000	2.681234000
H	0.535575000	-2.901987000	0.440521000
H	-1.491468000	0.011692000	-2.648813000
H	-3.908070000	0.001924000	-2.127121000
H	-5.011618000	-2.088868000	-1.374433000
H	-3.681758000	-4.178074000	-1.175604000
H	-1.273534000	-4.179754000	-1.725865000

D) **TCI**

E= -8828.007695 Ha

G= -8827.580764 Ha

H	0.675276000	-2.968140000	5.505010000
C	-0.327634000	-3.346716000	5.702127000
H	-0.193939000	-4.422882000	5.818090000
H	-0.720988000	-2.910545000	6.620379000
C	-1.380758000	-3.060622000	4.641114000
O	-2.221062000	-3.935917000	4.337344000
N	-1.337612000	-1.960234000	3.915329000
H	-0.665371000	-1.256846000	4.185979000
C	-2.324110000	-1.656463000	2.937336000
H	-3.330231000	-1.747941000	3.348341000
C	-2.121833000	-0.226814000	2.386349000
H	-2.910216000	-0.041083000	1.658276000
H	-2.250383000	0.483537000	3.205211000
C	-0.795695000	-0.000640000	1.730260000
N	0.322134000	0.485371000	2.403034000
H	0.347850000	0.758942000	3.379185000

C	1.364212000	0.559871000	1.573640000
H	2.348428000	0.916162000	1.819966000
N	0.954167000	0.140030000	0.380473000
C	-0.381498000	-0.211770000	0.446876000
H	-0.922887000	-0.569032000	-0.409458000
C	-2.429754000	-2.656719000	1.809315000
O	-3.477826000	-2.681568000	1.158444000
N	-1.458534000	-3.562248000	1.607202000
H	-0.661120000	-3.421302000	2.210526000
C	-1.441999000	-4.720505000	0.677370000
H	-1.266325000	-4.404615000	-0.350612000
H	-0.586081000	-5.360118000	0.889384000
H	-2.296532000	-5.383024000	0.814864000
H	-11.774155000	-2.855666000	-1.546721000
C	-11.699730000	-2.759695000	-0.463504000
H	-12.634810000	-3.076310000	-0.003414000
H	-10.991529000	-3.505230000	-0.102070000
C	-11.471233000	-1.297889000	0.035864000
O	-11.745858000	-0.916931000	1.155768000
N	-10.819357000	-0.515205000	-0.905093000
H	-10.535706000	-0.704385000	-1.856513000
C	-10.577069000	0.903377000	-0.451898000
H	-10.019977000	1.040751000	0.474968000
C	-9.731316000	1.608059000	-1.536021000
H	-10.225196000	1.493098000	-2.505504000
H	-9.704667000	2.674380000	-1.305962000
C	-8.302546000	1.072000000	-1.590536000
H	-7.796101000	1.257530000	-0.640308000
H	-8.279509000	-0.004552000	-1.771926000
S	-7.262376000	1.871050000	-2.862276000
C	-7.991855000	1.153499000	-4.368419000
H	-7.944200000	0.063705000	-4.330671000
H	-9.024575000	1.475947000	-4.503003000
H	-7.397753000	1.507784000	-5.211856000
C	-11.859818000	1.795314000	-0.229756000
O	-11.851095000	2.895214000	0.355995000

N	-13.076767000	1.296102000	-0.566499000
H	-13.236910000	0.375465000	-0.949993000
C	-14.396689000	1.927831000	-0.257542000
H	-14.272261000	3.010823000	-0.257703000
H	-15.016564000	1.668680000	-1.115847000
H	-14.780221000	1.618819000	0.714847000
H	6.448877000	1.056070000	-4.983870000
C	7.445468000	0.836199000	-4.601133000
H	7.846637000	-0.028476000	-5.128296000
H	8.020117000	1.758878000	-4.681926000
C	7.411243000	0.511011000	-3.098923000
O	8.430511000	0.479048000	-2.492182000
N	6.267989000	0.303291000	-2.498508000
H	5.502023000	0.408914000	-3.148323000
C	6.106402000	0.191734000	-1.070768000
H	6.578078000	1.095632000	-0.685202000
C	4.599911000	0.341846000	-0.656509000
H	4.393208000	-0.307842000	0.195784000
H	4.440199000	1.367684000	-0.328858000
S	3.321377000	0.045091000	-1.933737000
H	1.572695000	0.087967000	-0.447232000
C	6.715387000	-1.100183000	-0.518798000
O	6.664355000	-2.180041000	-1.180662000
N	7.387597000	-1.015368000	0.625127000
H	7.457674000	-0.091227000	1.025010000
C	8.314698000	-2.005604000	1.089027000
H	8.387560000	-2.126637000	2.169292000
H	7.905789000	-2.994169000	0.876771000
H	9.353470000	-2.007985000	0.758921000
H	5.529707000	3.297765000	2.787930000
C	4.464351000	3.097497000	2.673569000
H	4.304783000	2.030210000	2.511455000
H	3.878748000	3.264257000	3.578063000
C	3.762204000	3.809054000	1.479280000
O	3.104362000	3.160342000	0.752412000
N	3.911192000	5.111556000	1.232818000

H	4.577170000	5.652876000	1.765549000
C	3.193568000	5.791823000	0.142432000
H	3.133268000	5.112356000	-0.707303000
C	1.723576000	6.075968000	0.547324000
H	1.168551000	6.326176000	-0.362188000
H	1.295159000	5.144781000	0.921525000
C	1.483912000	7.208925000	1.544764000
H	1.922942000	8.144681000	1.192602000
H	0.407088000	7.370169000	1.636914000
S	2.155593000	6.995684000	3.236229000
C	1.327583000	5.456236000	3.734789000
H	0.243972000	5.553580000	3.643225000
H	1.671340000	4.606970000	3.144258000
H	1.580919000	5.284252000	4.781800000
C	3.795044000	7.047481000	-0.443562000
O	4.511943000	7.689888000	0.346739000
N	3.557507000	7.310968000	-1.688638000
H	3.116284000	6.621042000	-2.280914000
C	3.925870000	8.602683000	-2.229062000
H	4.826517000	8.933081000	-1.711200000
H	4.139276000	8.511609000	-3.294081000
H	3.118929000	9.321148000	-2.084744000
Se	3.370008000	-5.089237000	-0.882957000
Se	3.357278000	-2.412169000	-1.761861000
C	3.220569000	-4.395020000	0.908476000
C	4.151144000	-3.465328000	1.396796000
C	3.972348000	-2.874327000	2.646096000
C	2.870999000	-3.207762000	3.436112000
C	1.960433000	-4.156359000	2.970997000
C	2.135776000	-4.746005000	1.720051000
C	1.590303000	-2.482381000	-2.546552000
C	1.435868000	-2.299495000	-3.922723000
C	0.159047000	-2.266168000	-4.481976000
C	-0.964028000	-2.414689000	-3.670460000
C	-0.808379000	-2.608098000	-2.298105000
C	0.466248000	-2.648310000	-1.735397000

H	5.005283000	-3.193040000	0.789690000
H	4.696940000	-2.148581000	2.999031000
H	2.729052000	-2.741008000	4.403733000
H	1.106052000	-4.438273000	3.575227000
H	1.420997000	-5.476543000	1.365796000
H	2.308694000	-2.176944000	-4.551922000
H	0.044452000	-2.121027000	-5.549960000
H	-1.956705000	-2.379896000	-4.103485000
H	-1.680889000	-2.712592000	-1.663335000
H	0.588380000	-2.798436000	-0.670380000

E) **Pinhb**
E= -8828.011884 Ha
G= -8827.585001 Ha

H	0.584696000	-3.806348000	5.304238000
C	-0.435165000	-4.150652000	5.475531000
H	-0.350332000	-5.236768000	5.526613000
H	-0.813481000	-3.753346000	6.417436000
C	-1.468799000	-3.754140000	4.430996000
O	-2.345790000	-4.570879000	4.072755000
N	-1.372678000	-2.615068000	3.773151000
H	-0.671104000	-1.960192000	4.087543000
C	-2.339587000	-2.209056000	2.812774000
H	-3.350758000	-2.279638000	3.214944000
C	-2.156578000	-0.736146000	2.392191000
H	-2.822828000	-0.559548000	1.546339000
H	-2.510843000	-0.110384000	3.213076000
C	-0.774377000	-0.284835000	2.041372000
N	-0.144937000	0.715069000	2.775964000
H	-0.531438000	1.160938000	3.600239000
C	1.029703000	1.024262000	2.231353000
H	1.716341000	1.771108000	2.579889000
N	1.184906000	0.243300000	1.164396000
C	0.086408000	-0.582786000	1.023674000
H	0.015039000	-1.301786000	0.231248000
C	-2.483686000	-3.133579000	1.626040000

O	-3.528382000	-3.072121000	0.972208000
N	-1.552944000	-4.068082000	1.372164000
H	-0.753326000	-3.999737000	1.984989000
C	-1.583291000	-5.167718000	0.374068000
H	-1.387874000	-4.798851000	-0.632449000
H	-0.757900000	-5.856858000	0.549316000
H	-2.467221000	-5.798342000	0.469102000
H	-11.809758000	-2.709899000	-1.759983000
C	-11.736852000	-2.682793000	-0.672757000
H	-12.687573000	-2.984200000	-0.235054000
H	-11.064612000	-3.479820000	-0.355192000
C	-11.445855000	-1.265480000	-0.085391000
O	-11.709121000	-0.940705000	1.054758000
N	-10.754703000	-0.457612000	-0.975678000
H	-10.474804000	-0.601684000	-1.936059000
C	-10.451506000	0.918995000	-0.436826000
H	-9.893870000	0.974793000	0.497942000
C	-9.563703000	1.647315000	-1.470327000
H	-10.053185000	1.618770000	-2.448201000
H	-9.486941000	2.694293000	-1.172380000
C	-8.162202000	1.045283000	-1.548404000
H	-7.664606000	1.129946000	-0.579341000
H	-8.188893000	-0.013530000	-1.814047000
S	-7.059193000	1.887507000	-2.736308000
C	-7.790504000	1.326729000	-4.306737000
H	-7.798429000	0.236295000	-4.353523000
H	-8.802549000	1.711425000	-4.434500000
H	-7.160787000	1.713705000	-5.108911000
C	-11.694490000	1.852341000	-0.164813000
O	-11.639599000	2.913543000	0.486510000
N	-12.930626000	1.429636000	-0.534122000
H	-13.129738000	0.541891000	-0.972928000
C	-14.222606000	2.100283000	-0.190960000
H	-14.049887000	3.174629000	-0.125374000
H	-14.848894000	1.921425000	-1.064954000
H	-14.624711000	1.750809000	0.759989000

H	6.587870000	0.578794000	-4.907411000
C	7.571596000	0.291699000	-4.536080000
H	7.936493000	-0.556807000	-5.113479000
H	8.187340000	1.190808000	-4.559499000
C	7.514914000	-0.121485000	-3.056348000
O	8.528511000	-0.235747000	-2.450018000
N	6.360374000	-0.313413000	-2.472535000
H	5.603329000	-0.134546000	-3.116767000
C	6.186282000	-0.503502000	-1.054615000
H	6.695959000	0.353503000	-0.613885000
C	4.684367000	-0.524489000	-0.667818000
H	4.256106000	-1.499878000	-0.894269000
H	4.602219000	-0.355520000	0.406871000
S	3.599996000	0.728793000	-1.467792000
H	1.974142000	0.308287000	0.522162000
C	6.734115000	-1.852273000	-0.580066000
O	6.638336000	-2.886936000	-1.306110000
N	7.403316000	-1.866841000	0.568643000
H	7.512546000	-0.972545000	1.023772000
C	8.282609000	-2.923769000	0.974118000
H	8.344514000	-3.112951000	2.045390000
H	7.831374000	-3.878576000	0.701627000
H	9.322152000	-2.953078000	0.647317000
H	5.728711000	2.387322000	2.983221000
C	4.656037000	2.242370000	2.854363000
H	4.449945000	1.195153000	2.627420000
H	4.073742000	2.380517000	3.765609000
C	3.992841000	3.055327000	1.703312000
O	3.310491000	2.482051000	0.936703000
N	4.201214000	4.362422000	1.536326000
H	4.887817000	4.840176000	2.102544000
C	3.520426000	5.138590000	0.487210000
H	3.434347000	4.515049000	-0.402211000
C	2.061496000	5.461186000	0.904010000
H	1.521051000	5.780469000	0.007375000
H	1.594200000	4.529635000	1.228419000

C	1.863661000	6.550997000	1.957633000
H	2.349696000	7.482063000	1.658637000
H	0.794542000	6.757983000	2.048497000
S	2.502739000	6.220016000	3.642730000
C	1.578403000	4.711147000	4.057114000
H	0.504341000	4.872712000	3.945818000
H	1.890455000	3.872679000	3.435445000
H	1.797077000	4.478486000	5.100078000
C	4.180524000	6.399056000	-0.020327000
O	4.921249000	6.959817000	0.809204000
N	3.961634000	6.747455000	-1.247815000
H	3.493138000	6.114993000	-1.881824000
C	4.390220000	8.051522000	-1.708259000
H	5.301983000	8.309312000	-1.169056000
H	4.604976000	8.015264000	-2.776279000
H	3.615453000	8.795529000	-1.522887000
Se	3.212159000	-4.791500000	-1.475265000
Se	2.683896000	-0.370367000	-3.185656000
C	3.216456000	-4.007077000	0.285910000
C	4.406380000	-3.893305000	1.025138000
C	4.410836000	-3.329796000	2.299022000
C	3.225266000	-2.873044000	2.876194000
C	2.036796000	-2.986107000	2.155402000
C	2.031123000	-3.537143000	0.874872000
C	0.885599000	-0.656557000	-2.525094000
C	0.041171000	0.429298000	-2.275236000
C	-1.278008000	0.203837000	-1.889014000
C	-1.765626000	-1.099319000	-1.779477000
C	-0.923735000	-2.177269000	-2.042938000
C	0.407962000	-1.962475000	-2.401541000
H	5.335688000	-4.242017000	0.593976000
H	5.346773000	-3.253143000	2.842101000
H	3.225517000	-2.439974000	3.869622000
H	1.106150000	-2.640550000	2.588033000
H	1.099033000	-3.608822000	0.327707000
H	0.410349000	1.441920000	-2.380824000

H	-1.926881000	1.046463000	-1.681410000
H	-2.791291000	-1.275384000	-1.478125000
H	-1.297712000	-3.189777000	-1.961102000
H	1.068419000	-2.803109000	-2.576555000
